# Advances in the structures, mechanisms and targeting of molecular chaperones

**DOI:** 10.1038/s41392-025-02166-2

**Published:** 2025-03-12

**Authors:** Jinying Gu, Yanyi He, Chenxi He, Qiuyue Zhang, Qifei Huang, Shangjun Bai, Ruoning Wang, Qidong You, Lei Wang

**Affiliations:** 1 State Key Laboratory of Natural Medicines and Jiangsu Key Laboratory of Drug Design and Optimization, China Pharmaceutical University, Nanjing, China; 2 Department of Medicinal Chemistry, School of Pharmacy, China Pharmaceutical University, Nanjing, China; 3 School of Pharmacy, Nanjing University of Chinese Medicine, Nanjing, China; 4 Jiangsu Provincial TCM Engineering Technology Research Center of Highly Efficient Drug Delivery Systems (DDSs), Nanjing, China

**Keywords:** Medicinal chemistry, Target identification

## Abstract

Molecular chaperones, a class of complex client regulatory systems, play significant roles in the prevention of protein misfolding and abnormal aggregation, the modulation of protein homeostasis, and the protection of cells from damage under constantly changing environmental conditions. As the understanding of the biological mechanisms of molecular chaperones has increased, their link with the occurrence and progression of disease has suggested that these proteins are promising targets for therapeutic intervention, drawing intensive interest. Here, we review recent advances in determining the structures of molecular chaperones and heat shock protein 90 (HSP90) chaperone system complexes. We also describe the features of molecular chaperones and shed light on the complicated regulatory mechanism that operates through interactions with various co-chaperones in molecular chaperone cycles. In addition, how molecular chaperones affect diseases by regulating pathogenic proteins has been thoroughly analyzed. Furthermore, we focus on molecular chaperones to systematically discuss recent clinical advances and various drug design strategies in the preclinical stage. Recent studies have identified a variety of novel regulatory strategies targeting molecular chaperone systems with compounds that act through different mechanisms from those of traditional inhibitors. Therefore, as more novel design strategies are developed, targeting molecular chaperones will significantly contribute to the discovery of new potential drugs.

## Introduction

Molecular chaperones, mainly referred to as heat shock proteins (HSPs), are defined as proteins that interact with, stabilize, or assist another protein in acquiring its native functional conformation.^1–3^ When cells are exposed to stress, HSPs help proteins folding and maturation, and control the quality and degradation of proteins. These functions are essential for the modulation of protein homeostasis.^4,5^ Since HSPs were initially identified in *Drosophila*,^6^ great progress has been made toward understanding the structure and function of HSPs. Researchers have reported that HSPs are prevalent in various species ranging from bacteria to humans, and increasing numbers of HSP members have been identified. HSPs from mammals are classified on the basis of their molecular sizes into several families of proteins, including large HSPs (HSP110, GRP170), HSP90, HSP70, HSP40, small HSPs (sHSPs), and the chaperonin family proteins HSP60/HSP10. Apart from sHSPs, most other HSPs function in an ATP-dependent manner.^7^ Different members of the HSP family are located in different cellular structures. For example, major isoforms of mammalian HSP90 include cytosolic HSP90α and HSP90β, endoplasmic reticulum (ER)-resident glucose-regulated protein 94 (GRP94), and mitochondrial TNF receptor-associated protein 1 (TRAP-1).^8–10^ These isoforms differ in their functions and regulatory mechanisms within the cell, contributing in various way to the cellular stress response and protein homeostasis. Furthermore, the modulation of protein homeostasis by HSPs requires the assistance of other proteins, defined as co-chaperones to perform these diverse tasks. For example, HSP40 and large HSPs primarily function as co-chaperones of HSP70.^11,12^ The protein-protein interactions (PPIs) between HSPs and co-chaperones precisely regulate the quality control processes of client proteins, including protein phosphorylation, ubiquitination, and other post-translational modifications (PTMs).^13,14^ In summary, HSP family members and co-chaperones form complex molecular chaperone networks that regulate client proteins, maintaining overall protein homeostasis.

Abnormal expression or dysfunction of HSPs is closely related to the occurrence, development, and treatment response of various diseases.^15^ To date, many types of diseases have been reported to be related to HSPs, including cancers, neurodegenerative diseases, cardiovascular diseases, inflammatory diseases, metabolic diseases, infectious diseases, ocular diseases, skin diseases, and even rare diseases caused by genetic mutations.^16–18^ In a diseased state, HSPs expression and regulatory networks may be significantly altered, representing both potential targets for diseases treatment and biomarkers for disease diagnosis. In cancer patients, the expression of HSPs is usually elevated, which further contributes to cancer cells to survive, proliferate and invade.^19^ Among HSPs, HSP90 was found to have high expression in various cancers and confirmed as a potential drug target.^20^ In particular, the expression of HSP90 is strongly correlated with the occurrence and progression of liver cancer. Therefore, HSP90 can be used as a potential biomarker to diagnose liver cancer in early stage.^21,22^ The clinical characteristics of HSPs in different diseases are gradually becoming clear, providing hope for discovering strategies to diagnose and treat many diseases.^23–25^

HSPs regulate disease in important ways, making them important targets for drug development. To date, therapeutic strategies targeting HSPs have primarily focused on designing small molecule inhibitors to suppress HSPs.^26^ However, the precise mechanisms by which HSPs exert their effects are still unknown. Therefore, it is challenging to discovery more potent small molecule drugs targeting HSPs. Here, we summarize the structure and functions of different HSPs and the biological mechanisms of molecular chaperone cycles. We further analyze the precise regulatory mechanisms of PPIs between HSPs and co-chaperones in HSP molecular chaperone systems. Finally, we review current inhibitory strategies targeting HSPs and diseases regulated by HSPs. Among HSPs, HSP90 and HSP70 have been the focus of extensive research. Overall, we classify targeting strategies into four development stages: stage 1, which targets pan-isoforms of the HSP families (from 1990s); stage 2, which targets isoforms of the HSP families with high selectivity (from 2000s); stage 3, which targets PPIs between HSPs and co-chaperones (from 2010s); and stage 4, which involves the design of multi-specific molecules based on HSPs (from 2020s). By analyzing clinical research advances and existing strategies targeting the regulation of HSPs, we hope to provide insights into more potential drugs for clinical application in the future.

## Structural advances in molecular chaperones

**Fig. 1:**
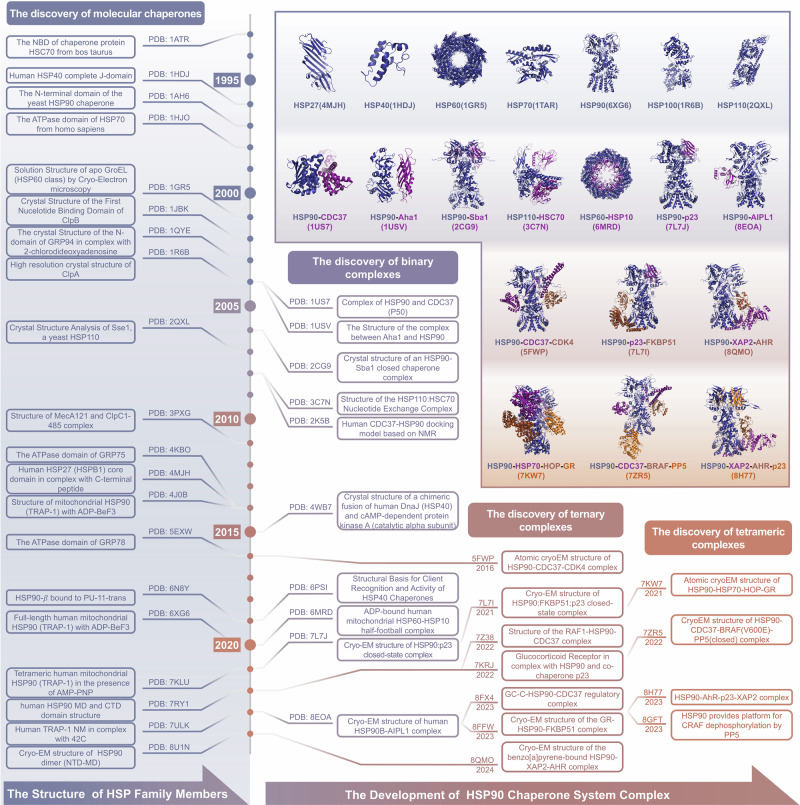
Historical timeline of the determination of the structures of molecular chaperones. The study of the structures of molecular chaperones can be traced back to the 1990s. In 1993, the crystal structure of HSC70, a member of the HSP70 family, was first obtained and resolved (PDB ID: 1ATR). The J domain of HSP40 (PDB ID: 1HDJ) and the N-terminal ATPase domain of HSP90 (PDB ID: 1AH6) were subsequently reported in 1996 and 1997, respectively. In the following years, the structure of more HSP members was gradually revealed. Besides, the complex structures of the PPIs related to the HSP molecular chaperone systems have gradually been obtained, which includes the discovery of binary complex structures, ternary complex structures and ternary complex structures. These discoveries provide more insights for understanding the mechanisms of molecular chaperone-dependent client protein regulation

Heat shock genes were first described in the *Drosophila* genome in 1960s and subsequently were identified.^27–29^ Subsequently, heat shock peptides were successfully expressed and isolated in vitro, marking the beginning of research on HSPs.^30,31^ With the discovery of more HSP members and their biological functions, understanding their three-dimensional structures has become increasingly intriguing. The first resolved crystal structure from the HSP family was that of HSC70 (PDB ID: 1ATR) in 1993, belonging to the HSP70 family. The J domain of HSP40 (PDB ID: 1HDJ) and the N-terminal ATPase domain of HSP90 (PDB ID: 1AH6) were subsequently revealed,^32,33^ opening the research field regarding the structure of HSPs. Importantly, the structures of more HSP members have gradually been obtained, including three other isoforms of HSP90, GRP94 in 2003,^34^ TRAP-1 in 2014,^35^ and HSP90βin 2019 (Fig. 1).^36^ As the structures of other isoforms were obtained, the degree of structural similarity between different isoforms of HSPs was gradually identified.

Protein engineering and crystal structure determination have made significant progress.^37,38^ Consequently, the structures of more HSP members and their isoforms have been revealed.^39–43^ The complex structures of the PPIs involving the HSP molecular chaperone systems have also been gradually obtained,^44,45^ elucidating the biological mechanisms of the molecular chaperone cycles. As the molecular chaperone cycle progresses, different complex structures are formed. The recently reported HSP90-kinase cycle is presented here as an example. When CDC37 recruits a kinase to HSP90 for folding, an HSP90-CDC37-kinase ternary complex is formed. For further modification of the kinase, the co-chaperone PP5 also binds to HSP90 to form a tetrameric complex. After the dephosphorylation modifications and folding of the kinase by HSP90, the kinase and PP5 are released from HSP90, whereas CDC37 remains bound to HSP90 in a binary complex. Similarly, different complex structures form in other HSP90-mediated molecular chaperone cycles.^46^ Overall, the development of complex structures includes the discovery of binary, ternary, and tetrameric complexes. Initially, most of the discoveries were binary complex structures. The structures of more binary complexes among molecular chaperone systems were determined, following the first HSP90-CDC37 (cell division cycle 37) binary complex structure in 2004.^47^ With the structure of the HSP90-CDC37-CDK4 (cyclin-dependent kinase 4) complex in 2016,^48^ research entered the stage of ternary complex discovery, which has revealed more complicated PPIs among molecular chaperone systems. Currently, structural research on molecular chaperone complexes has revealed the structures of even more complicated tetrameric complexes (Fig. 1), including the HSP90-HSP70-HOP-GR complex, the HSP90-CDC37-BRAF/CRAF-PP5 complex and the HSP90-AhR-p23-XAP2 complex.^46,49–51^ Their discoveries represent a new milestone in the field of molecular chaperone research, revealing the complete molecular mechanisms of chaperone-dependent client protein regulation (Table 1).

**Table 1:** Summary of characteristics, function, and representative structures of molecular chaperone

Family	Characteristics	Function	PDB ID
Small HSPs (HSPB1~HSPB10)	ATP-independent	Prevent proteins aggregation; Sequestrate misfolded proteins	4MJH, 5LUM, 6F2R 6BP9, 6DV5, 3J07, 6T1R
HSP40 (DnaJA/B/C)	Contain J domain	HSP70 co-chaperone; Accelerate ATP hydrolysis	1HDJ, 2CTQ, 2CTR 2M6Y, 4WB7, 7NDX
HSP60	ATP-dependent	Protein foldase; Prevent proteins aggregation	1GR5, 4PJ1 6MRC, 7AZP, 8G7O
HSP70 (HSP70i, HSC70, GRP75, GRP78)	ATP-dependent	Work as holdase and foldase; Promote polypeptides folding	1ATR, 1HJO, 1XQS, 2LMG 4JNE, 4PO2, 4KBO, 5EXW
HSP90 (HSP90α, HSP90β, GRP94, TRAP1)	ATP-dependent	Promote clients folding; Refold misfolded proteins; Help clients PTMs	2K5B, 1AH6, 1QYE, 1US7 1HK7, 1USV, 2CG9, 2O1V 4J0B, 4O04, 6N8Y, 6XG6, 7RY0, 8X2R, 5FWP, 7L7I, 7KW7, 7KRJ, 7Z38, 7ZR5, 8H77, 8GFT, 8FX4, 8QMO
HSP100 (ClpA/B/C/X)	ATP-dependent	Solublize proteins aggregates; Degradate misfolded proteins	1JBK, 1R6B, 3PXG 6W21, 6SFW
Large HSPs (HSP110, CRP170)	HSP70 superfamily	Co-chaperone of HSP70; Promote ATP-ADP exchange	2QXL, 3C7N, 6GFA

Data source: RCSB Protein Data Bank (https://www.rcsb.org/). If a same structure was released on both RCSB Protein Data Bank, only PDB ID was provided for space saving. Data were last updated on July 25th, 2024

### Discovery of the structures of molecular chaperone family members

#### Small heat shock proteins (sHSPs)

Small heat shock proteins (sHSPs) represent the first line of defense in protein homeostasis within the cell.^52–54^ To date, a total of 10 sHSPs (HSPB1-HSPB10) have been identified in mammals, ranging in size from 12 to 43 kDa.^55^ The structures of five sHSP family members, including HSPB1 (also known as HSP27),^56^ HSPB2, HSPB3,^57^ HSPB5,^58^ and HSPB6 (also known as HSP20),^59^ have been reported (Fig. 1). Three-dimensionally, sHSPs can be classified into three functional parts.^60^ A variable N-terminal sequence is followed by a conserved α-crystallin domain and a short C-terminal sequence.^39,61^ In particular, the α-crystallin domains of sHSPs have highly similar amino acid sequences.^62^ The α-crystallin is the core domain, representing a β-sheet sandwich consisting of eight antiparallel strands attached to an interdomain loop.^63,64^ As more high-resolution structures of sHSPs have been revealed, α-crystallin domains have been demonstrated to form dimers or tetramers, which are the building blocks of higher oligomers.^57,59,65^ The variable N- and C-terminal motifs are indispensable for sHSP subunit binding with higher oligomers, which represents a pivotal attribute of sHSPs. For example, a 24-mer of HSP27 is constituted by interactions within omnipresent and flexible N-terminal sequences, which is vital for the refolding and degradation of misfolded proteins.^61,66^

#### HSP40

HSP40s, also referred to as DnaJs, include three classes of members: DnaJA, DnaJB, and DnaJC.^67^ As an important class of co-chaperones, HSP40s are critical elements of the cellular chaperone network.^68–70^ In 1990, Ohtsuka et al. attempted to identify novel HSPs in mammalian cells and found a novel 40 kDa heat shock protein (HSP40, Fig. 1). HSP40 expression is triggered by a variety of stimulation and has been observed in multiple mammalian and avian cell types.^71^ Structurally, HSP40 includes three domains: a J domain, a glycine and phenylalanine (G/F)-rich region and a C domain consisting of four repeat motifs.^72^ Among the structural domains of HSP40, the J domain is highly conserved and functionally critical. As a co-chaperone of HSP70, the J domain of HSP40 mediates the interactions with HSP70 and activates HSP70 ATPase activity, thereby stabilizing interaction between HSP70 and nonnative proteins.^73,74^ The J domain is often observed in near the N-terminal regions of proteins.^75,76^ On the basis of differences in these regions, the HSP40 family has been divided into three distinct categories. Type I proteins have all three domains; type II proteins possess the J domain and G/F-rich region but not the C domain; and type III proteins contain the J domain alone.^77,78^ Human HSP40 is a type II protein. Therefore, HSP40, as a vital co-chaperone, can interact with HSP70 to accelerate the hydrolysis of ATP and facilitate co-translational folding of nascent polypeptides.^79^

#### HSP60

In addition to mammals, HSP60 is present in many other organisms, including fungi, plants and bacteria.^80,81^ Almost 50% sequence identity is shared between bacterial and mammalian HSP60.^82^ Therefore, most of the original knowledge about biological structures was derived from previous research on *Escherichia coli* GroEL and GroES, the bacterial homologs of HSP60 and HSP10.^83–85^ In 2015, the structure of the first human HSP60 was revealed, indicating that HSP60 monomers contain three domains, the equatorial, intermediate, and apical domains (Fig. 1).^86^ The equatorial domain contains nucleotide and substrate binding sites, the apical domain binds to HSP10, and the intermediate domain acts as a linker hinge.^87^ Among these domains, the equatorial domain of HSP60 is conserved throughout evolution. Functional region mutation studies reported that the residues of the ATP/ADP-binding site are the most evolutionarily conserved because of their importance for HSP60. Besides, some hydrophobic residues in the equatorial domain are also significantly conserved, contributing to binding of substrate proteins.^88,89^ Unlike GroEL and GroES, HSP60 and HSP10, respectively, form a stable heptameric single ring by self-assembly in the absence of ATP.^90^ When ATP binds, a double-ring structure is formed when HSP60 interacts with HSP10,^91,92^ which maintains their functional activity.

#### HSP70

Among the most ubiquitous molecular chaperones, multiple HSP70s are encoded by many species ranging from bacteria to humans.^93,94^ To date, over 20 members of the HSP70 family have been reported.^95,96^ Although the structure of ATPase domain from HSP70 family was solved as early as 1990,^97,98^ more information is needed to understand the structural composition and biological mechanism of HSP70 (Fig. 1). To date, more than 1000 crystal structures of HSP70 and related proteins have been described, and the sequences and structures of the HSP70 family proteins are highly conserved across all the species examined.^99^ Different HSP70 isoforms, including inducible HSP70 (HSP70i), heat shock cognate protein 70 (HSC70), glucose-regulated protein 78 (GRP78) and mitochondrial mortalin (GRP75),^100–102^ have been identified. These findings have facilitated a more comprehensive understanding of the biological functions of the HSP70 family. HSP70 contain two domains: a nucleotide-binding domain (NBD) at the N-terminus and a substrate-binding domain (SBD) at the C-terminus.^103^ Besides, the NBD and SBD are connected by a flexible linker that is important for allosterically regulation of HSP70.^104^ The NBD, weighing approximately 44 kDa, is the energy part. This domain provides energy for chaperone activity by binding ATP and hydrolyzing it to ADP. The NBD comprises two large lobes that bind the nucleotide. The bound nucleotide stabilizes this unique ADP-bound conformation for the NBD by forming extensive contacts with all subdomains.^105,106^ The SBD is also composed of two subdomains: a substrate binding domain (SBDβ) and a helical lid domain (SBDα), in which a client peptide-binding cavity is formed.^43^ In the nucleotide-free and ADP-bound states, SBDα docks onto SBDβ to fully enclose this cavity. This domain recognizes and binds polypeptide substrates so that HSP70s can assist in their folding. Among HSP70 family members, the N-terminal NBD functions by highly conserved mechanisms, whereas the C-terminal SBDs contributes to isoform specificity.^107^ The structures of full-length HSP70 are highly desirable for understanding the molecular mechanism of HSP70, although the isolated domain structures have been published.^100,108^

#### HSP90

HSP90 is one of the most prominent molecular chaperones and regulates many cellular processes under both physiological and stress conditions. The HSP90-encoding gene was first isolated from the *yeast Saccharomyces cerevisiae* in 1983^109^ and encodes a heat shock-inducible protein of 90 kDa, which was first used to quantify the heat shock response (HSR) in a eukaryotic organism.^110^ HSP90 family conserved from bacteria to humans and has more than 50% sequence identity between different members, implying that it is part of a fundamental biological process.^111^ In 1997, the structure of the N-terminal domain (NTD) of *yeast* HSP90 was first confirmed.^112,113^ Subsequently, the function of HSP90 have been gradually elucidated (Fig. 1). The major isoforms of HSP90 include HSP90α, HSP90β, GRP94, and TRAP-1, which share high sequence homology in the N‐terminal ATP‐binding site.^114^ HSP90 consists of three domains: the NTD, the C-terminal domain (CTD), and the middle domain (MD).^115^ Among them, the NTD that contains ATP-binding site, is highly conservative between HSP90 members. For example, the N-terminal ATP binding site of HSP90α and HSP90β are 95% identical, differing in only two amino acid residues.^116^ In eukaryotes, a flexible linker domain connects the NTD and MD of HSP90 (HSP90 MD).^117^ The deletion and truncation of the linker interferes with client protein activation.^118,119^ The NTD is rich in β-strands and forms a nucleotide-binding pocket, mediating the interaction of ATP with HSP90 and the consequent conformation change of HSP90. The ATP-binding site in the NTD is required for the chaperone cycle of HSP90.^120,121^ The HSP90 MD is essential for binding clients and ATP hydrolysis, which occurs only after the ATP-binding site in the NTD interacts with the MD.^122^ The CTD of HSP90 contains three key motifs: a calmodulin-binding site, an HSP90 homodimerization motif and a nucleotide-binding site. The calmodulin binding site may regulate the conformation of HSP90 by disrupting the intramolecular interaction.^123^ The homodimerization motif allows HSP90 to constitutively dimerize through two carboxy-terminal helices and form a four-helix bundle.^124^ Compared with the ATP-binding site in the NTD, the nucleotide-binding site in the CTD has different ligand specificities and functions as an allosteric modulator of N-terminal ATPase activity.^125^

#### HSP100

HSP100 chaperones belong to the AAA^+^ (ATPases associated with diverse cellular activities) superfamily.^126^ HSP100 consists of three main domains: the NTD, regulatory MD, and NBDs (Fig. 1).^16^ HSP100 members have two classes. Class 1 proteins have two NBDs and are present in plants and microbes (except viruses and *Archaea*), such as ClpA, ClpB, and ClpC, whereas class 2 proteins are characterized by a single NBD, including ClpX. These different NBDs share a small amount of sequence homology.^127–129^ In 2002, the first structure of the NBD of ClpB was revealed (Fig. 1), and a “see-saw” model was proposed to explain the mechanisms involved in its ATPase activities for chaperone functions.^130^ With reports of additional high-resolution structures, including ClpA^131^ and ClpB^132^ in complex with ClpP, the mechanisms and structures of the HSP100 chaperones are better understood. HSP100 chaperones typically form homohexamer rings containing substrate binding sites. HSP100 chaperones also possess highly mobile NTDs that may be involved in delivering substrates to the central channel or interacting with cofactors.^133^

#### Large HSPs

Large heat shock proteins (large HSPs) are highly expressed when stimulated by cytotoxic or proteotoxic stresses. HSP110 is a large HSP that has been well characterized in mammals. As an important co-chaperone of HSP70, HSP110 cooperates with HSP70 to restore protein folding and promote cell survival. The number of studies on HSP110 greatly increased after its cDNA sequence was cloned in the early 1990s.^134^ HSP110 is structurally similar to HSP70 (Fig. 1). The general structure of human HSP110 also contains N-terminal NBD, the SBDα region with acidic insertions and the β region (SBDβ). The HSP110 sequence shares 30-33% identity with other HSP70 members. Among the similar sequences, the N-terminal NBD is highly conserved between these molecules, which mediates the binding of ATP and determines their chaperone activity.^134^ The unique acidic insertion motifs have varying degrees of expansion, which results in much larger molecular weights for HSP110 and GRP170 than for HSP70.^135^ The SBDs of HSP110 are responsible for recruiting substrates harboring aromatic residues, and the distinct recognition motifs regulate substrate binding specificity.^136^ GRP170, another important large HSP, was first identified as a 170 kDa molecule that can be induced by glucose starvation.^137^ GRP170 has high sequence homology to HSP110. In contrast to HSP110, GRP170 has a C-terminal ER retention moiety that localizes GRP170 to ER. Consequently, GRP170 also represents a particular class of ER-located oncogenic proteins.

### Dissection of complex structures related to the HSP90 molecular chaperone systems

#### Binary complex structures

##### HSP90-CDC37 interaction

CDC37, a 50 kDa protein, was first discovered to be particularly associated with HSP90-dependent protein kinases in mammalian cells.^138^ CDC37 is an important co-chaperone of HSP90, regulating the HSP90 chaperone cycle.^139^ The functional dissection of CDC37 revealed a kinase-binding region at the N-terminus, whereas the rest of the protein was in interaction with HSP90.^140,141^ In 2004, the interaction between CDC37 MD (CDC37M) and the HSP90 N-domain (HSP90N) from *yeast* was mapped, and the core structure of HSP90-CDC37 complex was determined (Fig. 1). The structure revealed the mechanism by which CDC37 halts the HSP90 chaperone cycle, trapping the “lid” of the nucleotide binding site in an open conformation and keeping the “jaws” of the HSP90 molecular clamp apart to promote clients loading.^47^ The structure of HSP90N-CDC37M complex was subsequently obtained on the basis of the NMR backbone assignments of CDC37M and HSP90N.^142^ The NMR structure revealed that leu205 in CDC37 is key to allow complex formation, which can be used to investigate the differences in interactions among homologous proteins and provide insights for the discovery of anticancer inhibitors.

##### HSP90-Aha1 interaction

HSP90 chaperone activity is completely reliant on the binding and hydrolysis of ATP. Many co-chaperones can modulate HSP90 ATPase activity. Aha1, a novel co-chaperone, was identified and shown to be required for HSP90 activation, and it bound directly to HSP90, enhancing its weak intrinsic ATPase activity.^143^ However, the activation mechanism of the HSP90 ATPase cycle is poorly understood. To understand the interaction of HSP90 with Aha1 and activation of HSP90 ATPase, the complex structure of HSP90 MD and Aha1N- domain (Aha1N) was determined (Fig. 1).^144^ The structure reveals an extended interface with HSP90, which involves three subdomains of HSP90 MD. The interactions are concentrated between the hydrophobic side chains of Leu 315, Ile 388 and Val 391 from HSP90 MD and Ile 64, Leu 66 and Phe100 of Aha1. Importantly, Aha1N binding induces conformational changes in the catalytic loop of the HSP90 MD, suggesting more details of HSP90 ATPase activation.

##### HSP90-p23/Sba1 interaction

The p23 and its *S. cerevisiae* homolog Sba1 prefer to bind HSP90 in the presence of ATP, which stabilizes the state required for client-protein activation of HSP90.^145^ This regulatory property of p23/Sba1 increases the efficiency of the ATPase-dependent HSP90 cycle.^146^ The mechanism of this stabilization process remained unclear until the structure of the HSP90-p23/Sba1 complex was reported (Fig. 1). Pearl et al. obtained the structure of full-length HSP90 with p23/Sba1, which revealed the complex architecture of the closed state of the HSP90 chaperone stabilized by p23/Sba1.^124^ The structure contains HSP90 dimer with p23/Sba1 molecules arranged in a symmetrical fashion on both sides. p23/Sba1 lies in a depression at the junction of the two N domains of HSP90, forming two different surfaces. Residues 31-37 and 85-91 of p23/Sba1 and residues 12-21 and 151-155 of one HSP90N form a smaller interface. Residues 13-16 of p23/Sba1 N-terminal strand are related to a larger interface. Besides, a parallel arrangement occurs in the NTD of HSP90 that accompanies ATP binding, locking HSP90 into the closed conformation and slowing ATP turnover. Therefore, the binding of p23/Sba1 can stabilize the ATP-bound state of HSP90. Overall, the structure offers the first insight into HSP90 bound to ATP and confirms the ATPase-coupled molecular clamp mechanism.

##### HSC70-HSP110 interaction

In addition to the HSP90-related binary complexes that have been reported, the complex structures of other molecular chaperone family members have gradually been revealed. HSP70, another important molecular chaperone, also requires co-chaperone proteins to complete client folding and regulation.^147^ HSP110 is a nucleotide exchange factor (NEF) that exchanges ADP for ATP from the NBD of HSP70.^148^ To clarify the relation between HSP110 expression and HSP70, *yeast* HSP110 (Sse1)-bovine HSC70 (an HSP70 isoform) complex structure was determined (Fig. 1).^149^ Specifically, residues 568-579 of HSP110 SBDα interact with residues 278-279 and residues 289-304/307 of HSC70 NBD, which forms four charge pairs and eight hydrogen bonds. The critical linkers located between NBDs and SBDs connect each other, forming a short two-stranded antiparallel β sheet between HSP110 and HSC70. The complex features an electropositive pore that allows nucleotides to bind. In addition, more interactions between the NBD of HSP110 and HSC70 promote nucleotides exchange. When the HSP110 NBD is closed and the HSC70 NBD is open, the interaction with HSP70 and nucleotides become weak, representing a pre-release state. These results help to clarify the mechanism of nucleotides exchange of HSP70.

#### Ternary complex structures

##### HSP90-CDC37-CDK4 interaction

HSP90 assists in the folding, maturation and posttranslational modification of kinases. More than half of the proteins in the human kinome interact with HSP90 as client proteins through its co-chaperone CDC37.^150^ Owing to the dynamic properties of HSP90-client interactions, many efforts to obtain HSP90-CDC37-kinase complex structures were failed. In 2016, however, the HSP90-CDC37-CDK4 interaction complex was identified, beginning the discovery of more complex structures of molecular chaperone systems (Fig. 1).^48^ CDC37 is separated into two domains and surrounds HSP90 in this complex. In addition, CDC37M and the C-domain interact with the HSP90 MD in the closed state of HSP90, whereas CDC37 binding to the surface of the HSP90 NTD is accessible only in the open state. CDK4 also assumes a unique conformation in this ternary complex. The hinge region of CDK4 is fully unfolded, with the N lobe and C lobe completely separated and stabilized by novel interactions with HSP90 and CDC37. HSP90 protects the kinase in a trapped unfolded state by interacting with the exposed N- and C-lobe interfaces. CDC37 mimics part of the kinase N-lobe, which also confirms that phosphorylating CDC37 help to maintain kinase-bound conformation. The first ternary complex related to molecular chaperone systems successfully provides an integrated model of chaperone-kinase interactions.

##### HSP90-CDC37-RAF1 interaction

Rapidly accelerated fibrosarcoma (RAF) are a class of serine/threonine kinases, including ARAF, BRAF, and CRAF (also known as RAF1). RAF kinases can activate the mitogen-activated protein kinase (MAPK) pathway.^151^ RAF1, a component of the HSP90 and CDC37 complex, was previously identified in both the cytosolic and membrane fractions of cells.^152^ Generally, the HSP90-CDC37-bound RAF1 structure resembles the previously reported CDK4-HSP90-CDC37 structure. HSP90 dimer adopts a conformation similar to its closed state, and CDC37 is divided into two domains, which wrap around the HSP90 dimer. In addition, CDC37 stabilizes the open kinase domains by interacting with its loop in the NTD and the C-lobes of RAF1, similar to CDK4. Particularly, the C-lobes of CDK4 and RAF1 have unique conformations, which suggests that the specific kinase determines the stabilization of its C-lobe. The structural comparison also revealed that conformation of the N-lobe is different between CDK4 and RAF1, which depends on the secondary structure and interaction with HSP90 and CDC37 complex. The distinctions could have an impact on the kinase folding, suggesting the existence of other possible regulatory mechanisms and the need for further studies on the HSP90 folding efficiency for various kinases.

##### HSP90-FKBP51-p23 interaction

FK506-binding protein 51 (FKBP51), belonging to the immunophilin family, binds HSP90 and catalyzes the peptidyl prolyl isomerization of client proteins.^153^ FKBP51 was revealed in HSP90-p23 maturation complex.^154^ Many studies have shown that the tetratricopeptide repeat (TPR) of FKBP51 can interact with the C-terminal EEVD region of HSP90.^155^ However, it is unclear what structural basis they share beyond binding TPR-EEVD. The mechanism of FKBP51 recognizing and acting on HSP90-bound client proteins is also unknown. In the most recently reported ternary complex structure of the HP90-FKBP51-p23 interaction (Fig. 1), p23 interacts with the HSP90 NTDs,^156^ which is consistent with previous reports. p23 does not directly interact with FKBP51, which binds to the opposite HSP90 dimer interface. FKBP51 preferentially binds the closed HSP90 with ATP, further stabilized by the binding of p23. The majority of interactions between FKBP51 and HSP90 occur through the TPR domain of FKBP51 and the CTD of HSP90. Notably, these interactions enable FKBP51 to extend to the position adjacent to client binding loops in the HSP90 MD, potentially ensuring its peptidyl-prolyl isomerase activity for client proteins. In summary, the HSP90-FKBP51-p23 complex provides the details for co-chaperone interacting with HSP90.

##### HSP90-CDC37-GC-C interaction

Guanylyl cyclases (GCs) are a class of membrane receptors that determine the production of second messengers for signaling in mammalian physiology.^157^ GCs can be regulated by HSP90 and CDC37 via the kinase homology domain of these receptors.^158^ However, the structural mechanisms and physiological processes involved are still unclear. Garcia KC et al. presented the structure of GC-C (a member of the GC family) complexed to HSP90 and CDC37 in 2023 (Fig. 1), helping to study the details of GC-C interacting with HSP90 mediated by CDC37.^159^ As observed with most HSP90-CDC37 structures, HSP90 forms a canonical closed dimer conformation. The characteristic long α-hairpin of CDC37 extends outside from one edge of this dimeric core. Conversely, CDC37 interacts with the pseudo-kinase (PK) domain of GC-C. The C-lobe of the PK domain wraps around the CDC37N on one side of the dimeric HSP90 core, and the N-lobe interacts with the CDC37M on the opposite face, which transports the GC-C into the groove formed by HSP90 dimer. The first solved structure of a non-kinase client complexed with HSP90 and CDC37 shows that CDC37 can recruit various client proteins with considerable sequence variation.

#### Tetrameric complex structures

##### HSP90-HSP70-HOP-GR interaction

The interactive details of the molecular chaperone systems have gradually been revealed by many binary and ternary complex structures. However, the regulation of client folding and modifications requires the participation of more components. Therefore, more direct structural evidence is necessary for clarification details about molecular chaperone complex systems. The structure of HSP90-HSP70-HOP-GR is the first solved tetrameric complex structure related to molecular chaperone systems (Fig. 1).^49^ As a co-chaperone of HSP90, HSP70 functions early in protein folding and detects unfolded or misfolded proteins.^95^ The co-chaperone HOP (HSP90-HSP70 organizing protein) promotes client proteins to interact with HSP90.^49^ The glucocorticoid receptor (GR) is a steroid hormone-activated transcription factor.^160^ In the architecture of the tetrameric complex, the HSP90 dimer (HSP90α/β) is enclosed by HOP, GR and two HSP70s. Two HSP70s play crucial roles: one delivers GR, and the second is the scaffold for HOP. TPR2A and TPR2B domains of HOP respectively bind to EEVD terminals of HSP90 and HSP70, which is consistent with reports in the literature. Notably, HSP90 takes on a “semi-enclosed” state, rather than the fully closed ATP-bound state. The interactions between HOP, the HSP90α CTD and HSP90β MD maintain the special state. The high-resolution map also reveals the process of GR loading, where HSP70 firstly obstacles GR by catching GR pre-Helix 1 and then loads it onto HSP90-HOP. The structure presents a clear structural basis of chaperone-dependent client remodeling and establishes fundamental concepts of client recognition.

##### HSP90-CDC37-RAF-PP5 interaction

CDC37 functions as a co-chaperone that can recruit kinases to the HSP90 molecular chaperone system, which has been well characterized in structural biology. However, the posttranslational regulation of recruited kinases on the basis of the complex network structure of HSP90 remains unclear. Serine/threonine-protein phosphatase 5 (PP5) consists of a TPR domain that mediates its interaction with HSP90, enabling it to function as a co-chaperone of HSP90 to regulate the dephosphorylation of client proteins.^161^ Recently, two tetrameric complex structures related to the co-chaperoning function of PP5 were revealed, including the structure of HSP90-CDC37-BRAF(V600E)-PP5 reported by Laurence H. Pearl and the structure of HSP90-CDC37-CRAF-PP5 reported by David A. Agard (Fig. 1).^46,50^ In the cryo-EM structures, the TPR domain of PP5 interacts extensively with the CTD of HSP90 via an extended helix, which activates PP5 by completely separating the TPR domains and the catalytic domain of PP5. The active site in the catalytic domain of PP5 faces the RAF, resulting in efficient dephosphorylation at neighboring sites of the kinase domain. In addition, the complex structures suggest that CDC37 can be dephosphorylated only once the kinase has exited the HSP90 complex, which highlights the effect of PP5 in kinase and CDC37 dephosphorylation. Overall, the two cryo-EM structures showed that the role of HSP90 in regulating PTMs of clients.

##### HSP90-AhR-p23-XAP2 interaction

Aryl hydrocarbon receptor (AhR) is a key ligand-activated transcription factor participating to regulate a variety of biological functions.^162^ AhR was initially reported to mitigate the toxic effects of environmental pollutants.^163^ In the classic AhR signaling pathway, AhR first forms a cytoplasmic complex with HSP90, p23, and the AhR-binding protein XAP2 in its inactive state (Fig. 1). However, the key question of how AhR assembles with HSP90, p23, and XAP2 to form a cytoplasmic complex has long remained unresolved. Two structures of the HSP90-AhR-p23 complex with and without bound XAP2 were successfully obtained in 2023 (Fig. 1), and in these structures, the bridge motif and PAS-B domain of AhR form direct contacts with HSP90.^164^ The C-terminal flexible region of the AhR PAS-B structural domain tightly binds to XAP2, and there is also a potential interaction with p23. These interactions firmly fix AhR within the entire complex, helping it to exist stably in the cytoplasm for a long period awaiting ligand combination. These results give more appreciations of the AhR signaling pathway.

## Mechanisms of molecular chaperones

**Fig. 2:**
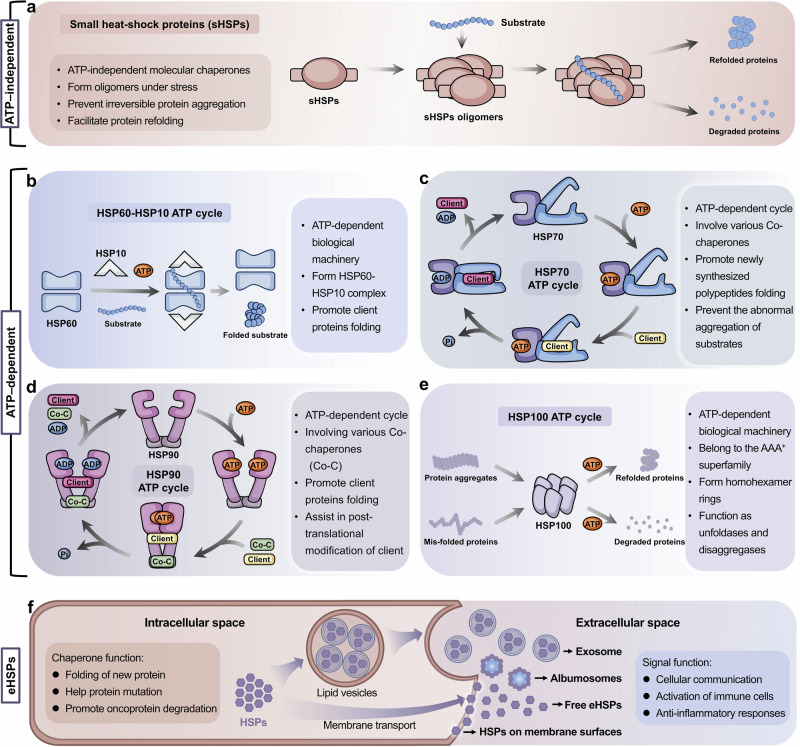
Mechanism of small heat shock proteins and ATP-dependent molecular chaperones. Molecular chaperones can be classified into two broad categories on the basis of their functional features: ATP-independent chaperones and ATP-dependent chaperones. Most HSP family members are ATP dependent, including HSP100, HSP90, HSP70, and HSP60, which are usually involved in more complex biological mechanisms. **a** Small HSPs can form oligomers through interactions with themselves or other small HSP members, working as holdases to prevent the aggregation and sequestration of misfolded proteins. **b** HSP60 can form an isolation chamber for the substrate protein through interacting with HSP10 to assist in protein folding. **c** HSP90 and HSP70 have a classic molecular chaperone cycle, respectively. HSP90 functions as a flexible dimer with an opening-closing dynamic cycle that allows interaction with client proteins and co-chaperones, promoting the correct folding and release of client proteins. **d** Unlike HSP90, HSP70 functions as a two-domain monomer. **e** HSP100 chaperone typically forms homohexamer rings containing substrate interaction sites to refold misfolded-proteins or disassemble irreversible protein aggregates. **f** The extracellular forms, functions and exocytosis pathways of eHSPs. Many HSPs are found in albumosomes, exosomes and oncosomes, and membrane surfaces, as well as free HSPs. Unlike intracellular HSPs, eHSPs are involved in cellular communication, immune cells activation, and anti-inflammatory responses

Molecular chaperones are critical for the correct folding, stabilization, and maturation of clients. Different molecular chaperones regulate substrate proteins by different biological mechanisms, which can be divided into two broad categories, including ATP-independent and ATP-dependent chaperones (Table 1). Small HSPs are representative ATP-independent chaperones, which are often regarded as passive holdases. ATP-dependent chaperones, including HSP60, HSP70, HSP90 and HSP100, require ATP hydrolysis for their activity (Fig. 2).

**Fig. 3:**
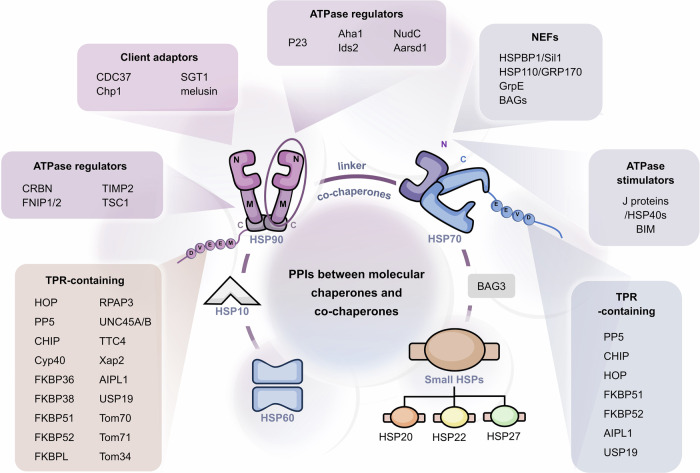
The PPIs between HSP molecular chaperones and co-chaperones are complex. Various types of co-chaperones and their respective binding sites on HSPs. On the basis of their function, the co-chaperones of HSP90 can be divided into three classes: HSP90 ATPase activity regulators that mainly bind with HSP90 NTD and MD, HSP90 client adapters that mainly bind with HSP90 NTD, and HSP90 chaperone cycle contributors containing TPR domains that bind with MEEVD motif of HSP90 CTD. The co-chaperones of HSP70 can be divided into three classes: including ATPase activity stimulators that bind with the HSP70 NBD, nucleotide exchange factors (NEFs) that also bind with the HSP70 NBD, and regulators containing the TPR domain that bind with the EEVD motif of the HSP70 CTD. Aside from HSP90 and HSP70, reported co-chaperones of other HSPs are very few. BAG3 reportedly interacts with sHSPs, including HSP20, HSP27 and HSP22. The best-studied co-chaperone of HSP60 is HSP10

Furthermore, HSPs regulate different types of clients, including kinases, transcription factors, E3 ubiquitin ligases and others. Therefore, the regulation of protein homeostasis by HSPs involves complex networks of PPIs. In some instances, HSPs can directly interact with clients to achieve their regulations,^165,166^ such as glucose transporter 1,^167^ Akt kinase^168^ and IκB kinase β.^169^ Compared with direct PPIs with clients, the PPIs of HSPs with co-chaperones tend to involve more unique functions of HSPs, which are essential for finer and more complex regulation of clients and the maintenance of protein homeostasis (Fig. 3). For example, HSP40 and large HSPs, as co-chaperones of HSP70, respectively activate ATP activity and promote nucleotide exchange in HSP70 molecular chaperone cycle. Co-chaperones, therefore, are non-client proteins that bind to HSPs and participate in the functions of HSPs. Special co-chaperones may also have chaperone activity and thus bind simultaneously to HSPs and client proteins simultaneously and prevent polypeptide aggregation. Many co-chaperones do not interact with client polypeptides and have a regulatory function in chaperone action, including catalyzing nucleotide binding, affecting the hydrolysis of ATP and physically linking molecular chaperones. In addition, co-chaperones largely determine the specificity of molecular chaperones to regulate substrates, which coordinate the cycle of binding and release to facilitate polypeptide folding, protein disassembly and posttranslational modification. Therefore, PPIs between molecular chaperones and co-chaperones constitute an important part of the molecular chaperone complex network and are critical for regulating the functionality of molecular chaperones in folding and proteostasis.^170^

### ATP-independent molecular chaperones

#### Small HSPs

Small HSPs (sHSPs) from different species are ATP-independent. They can form polydisperse and dynamic complexes with clients, acting as a first line of defense to prevent protein aggregation (Fig. 2a).^171^ The characteristic of sHSPs is their oligomerization ability. Through interactions with themselves or with other sHSPs, they can form homo or hetero-oligomers containing up to 50 subunits.^172^ Upon sensing stress, the N-terminal regions of sHSPs typically drive oligomers formation. Aromatic residues in N-terminal regions enable oligomers to interact with the solvent-exposed hydrophobic regions of nonnative clients, preventing irreversible protein aggregation and facilitating protein refolding. The oligomerization of sHSPs is a highly dynamic process. Overall, the action of sHSPs can be largely explained by the kinetic partitioning between the association rate of unfolded or misfolded client proteins, the rate of protein aggregation and clients refolding.^173^

### ATP-dependent molecular chaperones

#### HSP60

HSP60 can be classified into two different groups of chaperonins based on its cellular localization. Group I chaperonins are located primarily in the mitochondria, whereas group II chaperonins are found mainly in the cytoplasm of eukaryotes.^174,175^ With the assistance of the HSP10 co-chaperone, HSP60 exhibits chaperone activity and promotes the folding of substrates, and this process involves ATP-driven biological machinery.^176^ During their functional cycles, unfolded client proteins are stabilized through their interactions with the equatorial domain of HSP60, which opens a double-ring structure in the nucleotide-free state (Fig. 2b). HSP60 forms a double-rings structure after ATP binding to equatorial domain of HSP60. Then, HSP60 recruits HSP10 by its apical domain to form a closed double-ring football-shaped structure. ATP is subsequently hydrolyzed to ADP, and the apical domain of HSP60 then changes conformation, resulting in the release of HSP10 and ADP and the formation of a bullet-type complex. At the same time, the substrate protein is folded and released.^177–179^ In addition to assisting in the folding of client proteins, HSP60 plays a vital role in the assembly, transport and interaction of several cellular proteins, regulating tumor progression.^180^

#### HSP70

HSP70 occurs in virtually all organisms. The key to HSP70 function is its transient substrate binding and its allosteric ATP-dependent cycles between high-affinity and low-affinity states (Fig. 2c). When ATP binds, the SBD is in the open conformation, in which SBDα and SBDβ are separated and bind to different faces of the NBD. The open conformation results in low affinity and rapid exchange rates with substrate proteins. The co-chaperone HSP40 helps increase ATP hydrolysis, resulting in the production of ADP. Once they enter the ADP-bound state, SBDα and SBDβ connect closely, forming a closed HSP70 structure with high substrate affinity and consequently slow exchange rates. Overall, structural changes in HSP70 prevent the abnormal aggregation of substrate proteins and promote the formation of native proteins.^95,181^ When the folding and maturation of substrates are complete, nucleotide exchange factors (NEFs), which constitute another vital co-chaperone of HSP70, bind the NBD of HSP70 and help with the release of ADP. As a result, the substrate-binding pocket of HSP70 is opened, and the folded substrate protein is released. This process of transformation between the low-affinity ATP-bound state and the high-affinity ADP-bound state drives the cycle of interaction between HSP70 and its substrate proteins.^182^ The basis for the biological function of HSP70 is its ability to recognize specific substrates. Research has indicated that the conformation of client proteins drives the recognition of HSP70, which highlights that the conformation selection mechanism determines the specificity of HSP70 substrate regulation.^183^

#### HSP90

HSP90 is abundant in eukaryotes, regulating the activity of numerous client proteins by ATP-dependent way (Fig. 2d). HSP90 differs from other HSPs. It does not promiscuously interact with nascent or unfolded polypeptides. Instead, HSP90 specifically recognizes partially folded client on the basis of interactions with many co-chaperones. Moreover, HSP90 seizes client proteins from HSP70 that associate with newly synthesized polypeptides.^184^ HSP90 functions as a homodimer, and its mechanism involves significant conformational changes. When not bound to ATP, the HSP90 homodimer adopts a V‑shaped open conformation. In the case of ATP binding, an intermediate state is formed as the ATP lid closes over the bound nucleotide.^124^ The intermediate state specifically associates with unfolded client proteins in the substrate binding site of the HSP90 MD with the assistance of co-chaperones.^122^ Subsequently, HSP90 ATPase activity is activated by the dissociation of co-chaperones, which helps the formation of closed HSP90 homodimer and facilitates unfolded substrates to interact with HSP90 MD. Finally, ATP hydrolysis leads to substrate folding, leading to the secretion of mature clients.^124^

#### HSP100

HSP100, from bacteria, yeast, and plants, constitute a unique class of ATP-dependent molecular chaperones (Fig. 2e). Although it has not yet been found in humans and other animals, HSP100 has been confirmed as a drug target against human infectious diseases.^185,186^ HSP100 chaperones harness metabolic energy to facilitate the unfolding of misfolded polypeptides.^187^ The functions of the unfoldase and disaggregase of HSP100 include the unique ability to resolubilize and reactivate aggregated proteins.^188^ The HSP100 family includes many members, and their biological mechanisms differ structurally. ClpB, a bacterial homolog, is a well-studied member of the HSP100 family. Upon binding ATP, ClpB assembles into a hexameric complex that can specifically recognize aggregated proteins and move downward along the central translocation channel. ClpB functions as an unfoldase and disaggregase through direct contact with substrates. In addition, ClpB-mediated protein unfolding and disaggregation are linked to the activity of other chaperones, such as HSP70. The regulatory MD of ClpB interacts with HSP70 to pass along polypeptides resulting from the dissociation process for proper refolding in HSP70 chaperone systems.^189^

### Extracellular HSPs

Under physiological conditions, low concentrations of HSPs promote protein folding, maturation and degradation. The overexpression of HSPs maintains protein homeostasis when cells are under stress.^190^ Although intracellular cytoprotective functions of HSPs are the best studied, many studies have also emphasized the major roles of secreted HSPs in the extracellular space (Fig. 2f). Initially, in 1989, HighTower and Guidon found the presence of HSP70 in an extracellular medium.^191^ As additional evidence became available, interest in extracellular HSPs (eHSPs) grew. Many HSPs, including HSP90, HSP70, HSP60, small HSPs and large HSPs, have been found in albumosomes, exosomes, and membrane surfaces, as well as occurring as free HSPs, which are collectively called eHSPs.^192–194^ Although the mechanism by which HSPs are secreted from the intracellular space to the extracellular space is poorly understood, three possible mechanisms have been reported, including translocation across the plasma membrane, release associated with lipid vesicles, and passive release after cell death by necrosis.^195^ Under pathological conditions, the secretion of eHSPs increases. The primary roles of eHSPs appears to be related to cellular communication and anti-inflammatory responses. Studies reported that eHSPs, including eHSP90α, eHSP70, eHSP110, and eHSP27, can induce macrophages to polarize immunosuppressive and proangiogenic phenotypes, which is conducive to cancer development.^196–198^ Among these proteins, eHSP27 has been confirmed to contribute to myocardial functional injury, and is involved in the mediation of proinflammatory effects.^199^ Besides, eHSP90α was found to mediate house dust mite-induced human bronchial epithelial barrier dysfunction.^200^ Significantly elevated eHSP90α was also found in pediatric patients with severe sepsis.^201^ In addition, eHSPs have been associated with several diseases, including autoimmune diseases, diabetes, cancer, liver, pancreas and kidney disorders, and cachexia.^195,202^ Therefore, eHSPs may become potential therapeutic targets for the treatment of diseases.

### PPIs between molecular chaperones and co-chaperones

#### Small HSP co-chaperones

Unlike the complicated HSP70 and HSP90 molecular chaperone networks, sHSPs function only as holdases. They mainly bind and protect denatured or nonnative proteins from aggregation. Therefore, sHSPs in many different organisms constitute a large and enigmatic class within the chaperone network.^171,203,204^ Small HSPs lack enzymatic activity, which seems to be necessary for other molecular chaperones to actively remodel or refold substrates in mammalian cells. Therefore, sHSPs were reported to interact with other cellular chaperones to assist in refolding nonnative proteins.^205^ The Bcl-2-associated anthanogene (BAG) family comprises stress-inducible proteins (Fig. 3), characterized by a conserved BAG domain, that can bind to HSP70 as co-chaperones.^206^ BAG3, belonging to BAG family, contains two special motifs in its N-terminus. BAG3 was reported to bind to sHSPs, including HSP20, HSP27 and HSP22 (HSPB8).^207^ BAG3 has been found to bridge HSP22 and HSP70 simultaneously and conciliate two families to refold denatured luciferase. Therefore, BAG3, as an important co-chaperone, is the connector among both essential chaperone proteins.^208^

#### HSP60 co-chaperones

HSP60 interacts with many proteins. For example, anterior gradient 2 (AGR2), a member of a family of chaperone‐like proteins, has been reported to interact with HSP60.^209^ However, the biological mechanisms of most interaction partners, including AGR2, are still unclear. The most thoroughly co-chaperone of HSP60 is HSP10, which plays an indispensable role in assisting HSP60 in protein folding. The protein–protein interactions of the HSP60/HSP10 complex cause the formation of two heptameric double rings that are stacked back to back and consist of the large subunit of HSP60 and the small subunit of HSP10 (Fig. 3). The ring structures of HSP60 and HSP10 enclose an inner cavity that accommodates the substrate proteins.^86,210^

#### HSP90 co-chaperones

HSP90 is one of the most studied molecular chaperones, with over 50 co-chaperones described. In the HSP90 cycle, the first tier of modulation of co-chaperones includes regulating its ATPase activity, assisting in client recruitment and performing other specialized functions. Specific posttranslational modifications of co-chaperones to client proteins form a second regulatory tier in the intricate chaperone networks of HSP90, which contributes to the maturation of the client protein.^211^ Overall, co-chaperones of HSP90 includes three functional groups: ATPase activity regulators, client adapters, and HSP90 chaperone cycle contributors (Fig. 3).

##### HSP90 ATPase activity regulators

During the HSP90 ATPase cycle, HSP90 undergoes conformational transitions regulated by co-chaperones (Fig. 3).^120^ Aha1 is an important activator of HSP90 ATPase. The NTD of Aha1 interacts with the HSP90 MD, while the CTD dynamically binds to the HSP90 NTD. These interactions facilitate dimerization of the HSP90 N-terminus, significantly accelerating its ATPase activity and shifting its equilibrium toward the closed conformation of HSP90. The activity of activator of HSP90 ATPase 1 (Aha1) contributes to the folding and maturation of client proteins. The down-regulation of Aha1results in inhibition of HSP90 ATPase activity, affects the folding of cystic fibrosis transmembrane conductance regulator (CFTR).^212^ Ids2 is another co-chaperone that enhances the chaperone activity of HSP90. The interactions between the middle region of Ids2 and the HSP90 NTD stimulate the ATPase activity of HSP90, which recruits the client protein Atp3 to the HSP90 folding system. Unlike Aha1, Ids2 is a mitochondria-dominant HSP90 co-chaperone and is important for mitochondrial function.^213^ However, when ATPase activity is suppressed by co-chaperones, HSP90 is stabilized in a client-binding state.^214^ p23 is central to the inhibition of HSP90 ATPase activity and contains a folded cysteine- and histidine-rich domain (CHORD) that binds to HSP90 MD and specifically affects the NTD-MD arrangement of HSP90 in the closed state. The interactions of p23 and HSP90 led to a 50% reduction in the ATPase rate. *Yeast* p23 enters at a late stage of HSP90 cycle, stabilizing the closed and client-engaging state of HSP90 and preventing the premature dissociation of client proteins from HSP90.^215^ In addition, alanyl-tRNA synthetase domain-containing 1 (Aarsd1) is a novel HSP90 co-chaperone. Similar to p23, Aarsd1 also inhibits HSP90 ATPase activity. Notably, Aarsd1 has been identified as a muscle-specific isoform and plays its role during muscle differentiation.^216^ Besides, the p23 domain-containing protein nuclear distribution gene C (NudC) influences the ATPase activity of HSP90. Its inhibition of HSP90 chaperone function results in stabilization of the client protein LIS1.^217^ The E3 ubiquitin ligase component cereblon (CRBN) is a conserved regulator, and CRBN-based proteolysis-targeting chimeras (PROTACs) have already been developed as potential evolutionary therapeutic agents.^218^ Recent findings have revealed that CRBN, as an HSP90 co-chaperone, specifically interacts with the MD of the ATP-bound and closed states of the HSP90 dimer. The interactions of CRBN with HSP90 attenuate HSP90 ATPase activity, counteracting the negative effect of Aha1 on client protein stability.^219^ Other novel co-chaperones that decelerate but do not completely inhibit the HSP90 chaperone cycle include folliculin-interacting protein 1 (FNIP1), tissue inhibitor of metalloproteinases-2 (TIMP2) and tuberous sclerosis complex 1 (TSC1), which binds to HSP90 MD and promotes the folding of related clients.^220–222^

##### HSP90 client adapters

HSP90 can regulate a variety of substrate proteins, including kinases, transcription factors, cyclin proteins, and receptor proteins, which require specific co-chaperones to recruit and form molecular chaperone complexes (Fig. 3).^223^ CDC37 is a co-chaperone for specifically recruiting kinase proteins, which contains a unique subunit that selectively binds kinases clients. For example, the specific recruitment of RAF and cyclin-dependent kinase (CDK) by CDC37 facilitates the recognition and binding of HSP90 to these kinase clients,^48,151^ which belongs to the first tier of HSP90 modulation. SGT1 also acts as a client adapter of HSP90, which consists of an N-terminal TPR domain, a central CHORD and SGT1 domain (CS domain) and a C-terminal SGT-specific (SGS) domain. Apart from the interaction between the TPR and the C-terminal tail of HSP90, the central CS domain interacts with a distinct site on the HSP90 NTD, which recruits SGT1 to HSP90. SGT1 is a co-chaperone that links ubiquitin ligases to the substrate-specific arm of HSP90 molecular chaperone system complexes, providing multiple complementary routes for the ubiquitination of HSP90 client proteins.^224–226^ CHORD-containing protein-1 (Chp-1) and melusin, which bind to the HSP90 NTD, are two other CHORD domain-containing HSP90 co-chaperones in mammals that also interact with the co-chaperone SGT1 and provide more complexity to the dynamics of the HSP90 cycle.^227,228^ FKBPs, including FKBP36, FKBP38, FKBP51, and FKBP52, are members of the immunophilin protein superfamily and are characterized by the presence of TPR domains. The TPR domains of these proteins interact with the Met-Glu-Glu-Val-Asp motif (MEEVD) of the HSP90 CTD, representing a large group of co-chaperones that interact with HSP90 via the TPR domain.^229,230^ FKBP-like (FKBPL) is a novel FKBP protein that, as a co-chaperone of HSP90, regulates the stability of nuclear hormone receptor-based HSP90 complexes, including GR, androgen receptor (AR) and estrogen receptor (ER) signaling.^231–233^ FKBPL is also an important adapter to regulate the protein p21. Studies have suggested that FKBPL recruits newly synthesized p21 to form a trimeric complex with HSP90, which prevents its proteasomal degradation and initiates cell cycle arrest following irradiation.^234,235^ Mitochondrial preproteins contain specific targeting signals.^236^ The preprotein translocases of the mitochondrial outer membranes (Toms), including Tom70, Tom72 and Tom34. Toms are novel co-chaperones of HSP90, specifically recognizing HSP90 and facilitating the import of mitochondrial preproteins.^237,238^ The three TPR motifs in the NTD of TOMs interact with the C-terminal EEVD motif of HSP90.^239^ The interactions of HSP90-TOMs ensure the translocation of preproteins into mitochondria and protect them from aggregation in the cytosol.^240,241^ Hepatitis B virus X-associated protein 2 (XAP2) shares homology with the immunophilin FKBP52. XAP2, as a co-chaperone, has been demonstrated to exist in complexes with HSP90 and the aryl hydrocarbon receptor (AHR), which is a ligand-activated transcription factor.^242^ The structure of the HSP90-XAP2-AHR complex revealed that XAP2 also interacts directly with the EEVD motif of HSP90 via its TPR domain, indicating the client-recruiting adaptive mechanism of co-chaperones in the HSP90 cycle.^243^ Mang of the identified co-chaperones of HSP90 contain TPR domains, including UNC-45 homolog A/B (UNC45A/B, from *C. elegans*), aryl hydrocarbon receptor-interacting protein-like 1 (AIPL1) and RNA polymerase–associated protein 3 (RPAP3), which interact with the HSP90 CTD on the basis of their TPR structures and play different roles in recognizing and regulating substrates.^229,244,245^

##### HSP90 chaperone cycle contributors

In addition to the ATPase activity regulators and client adapters of HSP90 mentioned above, HSP90 chaperone cycle contributors constitute a significant class of co-chaperones that contribute to the proper function of the HSP90 chaperone cycle through their enzymatic activities and structural properties (Fig. 3). Protein phosphatase 5 (PP5) is a serine/threonine protein phosphatase that functions as a co-chaperone of HSP90 by simultaneously performing dephosphorylation.^246^ The HSP90 MEEVD motif recognizes the PP5 TPR domain, leading, in this case, to PP5 activation and completion of the dephosphorylation regulation of specific substrates.^247^ Structural studies has confirmed that PP5 interacts with HSP90 and can regulate the dephosphorylation of specific substrates, including CRAF and BRAF, which are crucial for the maturation of these client proteins.^46,50^ The C-terminus of the HSC70-interacting protein (CHIP) is a ubiquitin ligase that contains an N-terminal TPR domain, a central α-helical domain, and a C-terminal U-box ubiquitin ligase domain, which interact with HSP90 through the TPR domain and U-box domain.^248,249^ CHIP, as a co-chaperone of HSP90, can ubiquitinate clients and deliver them to the proteasome for degradation. Therefore, CHIP is a quality control regulator of the folding pathway. In addition, CHIP expression in the HSP90 chaperone system elicits the release of p23, indicating that CHIP remodels HSP90 heterocomplexes in a particular manner.^250^ In contrast to CHIP, ubiquitin-specific protease 19 (USP19) is a deubiquitinating enzyme located at the ER.^251^ Ye et al. reported that HSP90 binds the catalytic domain of USP19 to promote substrate association, which suggests that USP19 is a specific co-chaperone of HSP90.^252^ Cyclophilin 40 (CYP40) contains an N-terminal peptidylprolyl isomerase (PPIase) domain and a C-terminal TPR domain and is an immunophilin co-chaperone of HSP90.^253^ The PPIase activity of CYP40 speeds rotation around the peptidyl prolyl amide bond, resulting in more efficient folding of peptide chains in the HSP90 system.^254^ Some studies showed that specific inhibition of the PPIase activity of CYP40 excludes a role for immunophilin catalytic activity in chaperone function.^255,256^ There is also a class of special co-chaperones that function as linkers in the molecular chaperone cycle. HSP70 and HSP90 organizing protein (HOP), as a linker containing two TPR domains, mediate the interaction of HSP70 and HSP90. The TPR2B and TPR2A domain of HOP specifically recognizes the C-terminal of HSP70 and HSP90.^155,257^ The PPIs between HOP and HSP90 can stabilize an alternate HSP90 open state in which hydrophobic client-binding surfaces converge. With the binding of HSP70, client proteins are loaded from HSP70 onto HSP90. The HSP90-HOP-HSP70-client loading complex promotes the transformation and maturation of client proteins.^49,258,259^ Small glutamine-rich TPR-containing protein (SGT) is a co-chaperone that interacts with both HSP90 and HSP70. SGT also mediates the interaction between HSP90 and HSP70, which provides a platform for the loading of substrates from HSP70 to HSP90.^260,261^ Similarly, tetracopeptide repeat protein 4 (TTC4) is a nucleoplasmic protein known as a co-chaperone of HSP90 that contains a TPR domain. TTC4 forms a link between HSP90 and DNA replication, which allows HSP90 to regulate the replication of diverse herpesviruses.^262,263^

#### HSP70 co-chaperones

Like HSP90, HSP70 also requires multiple co-chaperones to assist in chaperone cycle. In the chaperone cycle, HSP70s first bind selectively to unfolded substrate polypeptides, and their activity is controlled by ATP binding and hydrolysis. ATP-ADP exchange in HSP70 determines the release rate of folded substrates. In addition, some co-chaperones are important for the regulation of HSP70 function because of their structural features and enzymatic activities. Therefore, co-chaperones involved in HSP70 regulation can be classified into three types on the basis of their roles in the HSP70 molecular cycle: ATPase activity stimulators, nucleotide exchange factors and other important regulators containing the TPR domain (Fig. 3).

##### ATPase activity stimulators

When ATP binds, HSP70 undergoes a rapid exchange of polypeptide substrates, but it generally has very low basal ATPase activity.^264^ ATP hydrolysis can induce close of the substrate-binding chamber and the locking of relevant substrates (Fig. 3), which is essential for the activity of HSP70.^265^ Therefore, ATP hydrolysis is the rate-limiting step in the ATPase cycle of HSP70. Members of the HSP40 family in eukaryotes are also known as J domain-containing proteins (JDPs), which is the largest class of HSP70 co-chaperones and primarily activates the ATPase activity of HSP70.^266^ Besides, HSP40 can stabilize the interaction between HSP70 and substrates. HSP40 contains a conserved J domain that interacts with the motif between the NBD and the SBD of HSP70, which stimulates the uncoupling of the NBD from the SBD, speeds up the hydrolysis of ATP, and changes the substrates affinity.^267^ The stimulation of ATPase activity was first recognized in the DnaJ/DnaK complex, which is a bacterial HSP40/HSP70 homolog.^268,269^ In humans, 41 HSP40 family members have been revealed through genome-wide analysis.^78^ Some studies found that the function of HSP40 is not limited to stimulating ATP hydrolysis. The conformational cycle of the HSP70-HSP40 complex also drives HSP70 to fulfill its molecular chaperone function.^270,271^ Bcl-2 interacting mediator (BIM), a Bcl-2 family member, has also been identified as a positive co-chaperone that promotes the ATPase activity of HSP70. BIM contains a distinct Bcl-2 homology 3 (BH3) domain that physically binds to the NBD of HSP70. In addition to increasing the ATPase activity of HSP70, BIM allosterically regulates the structure of HSP70 to simultaneously stabilize the binding of client proteins.^272,273^ Experimental studies showed that the formation of the HSP70-BIM-protein kinase B (AKT) complex reduces the degradation of the oncogenic client AKT.^274^

##### Nucleotide exchange factors (NEFs)

The ATPase cycle of HSP70 refers to the process in which ADP and Pi combine to form ATP after hydrolysis, while the substrate bound chamber of the SBD opens to release substrate. The starting point of the cycle is then reestablished for a new round of substrate binding. Therefore, the rate of substrate release is limited by the dissociation of ADP under physiological conditions. NEFs primarily act as co-chaperones for HSP70 by promoting the release of ADP, which accelerates ADP-ATP exchange (Fig. 3). Mechanistically, NEFs can interact with NBD of HSP70, which stimulates the opening of the interdomain cleft, releases ADP, and thereby causes the substrate to be released from the SBD of HSP70.^275^ The function of GrpE, the sole bacterial NEF, has been well studied. GrpE consists of an α-helical dimerization domain and a β-structure that inserts into the nucleotide-binding pocket of the HSP70 homolog DnaK. The PPIs force the NBD of HSP70 to open, significantly reducing in its affinity for nucleotides and accelerating of their dissociation.^276,277^ Unlike bacteria, many HSP70 NEFs have been discovered in eukaryotic cells. The most studied NEFs mainly include the HSP110/GRP170, HSP70 binding protein 1/Sil1 (HSPBP1/Sil1), and BAG families. Although the mechanism by which HSP110 and its ER GRP170 act as NEFs is unclear, the structure of *yeast* HSP110 (Sse1) in complex with bovine HSC70 provides some insights for understanding how HSP110/GRP170 induces nucleotide release from HSP70. First, the binding of ATP to Sse1 induces the closed conformation. The SBD β-structure of Sse1 subsequently interacts with the intermediary sequence between the SBD and NBD of HSC70 to induce the latter to open and facilitates nucleotide release.^108,149,278^ HSPBP1 and Sil1 respectively represent the cytosolic and ER forms of an NEF family with armadillo repeat architecture, which interacts with the NBD subdomain of HSP70 and reduces the affinity between nucleotides and HSP70.^279^ BAGs, including BAG1-6, are the most highly variable class of eukaryotic NEFs. Human BAG1 was the first confirmed eukaryotic NEF.^280,281^ Structural analysis revealed that the respective BAG domains have a conserved sequence and similarly targeted the NBD subdomain of HSP70, which induces conformational changes in HSP70 and exhibits NEF activity in BAGs.^282,283^

##### Regulators containing the TPR domain

Like HSP90, many co-chaperones containing the TPR domain play significant regulatory roles for HSP70, including CHIP, HOP, and PP5, which facilitate the recruitment of substrates and lead to specific catalytic actions on protein complexes by binding to the C-terminus of HSP70 (Fig. 3). CHIP is an E3 ubiquitin ligase related to the degradation of substrate proteins. Besides, CHIP also inhibits HSP70 ATPase activity activated by co-chaperone HSP40s.^284^ When HSP70 combines with nonrenewable proteins, CHIP uses its E3 ubiquitin enzyme activity to degrade them.^285^ Similarly, HOP couples HSP70 with HSP90, and PP5 facilitates the dephosphorylation of substrates of HSP70.^155,286^ In addition, other TPR domain-containing proteins, including FKBP51, FKBP52 and AIPL1, have also been reported to interact with HSP70, and assist HSP70 in completing the folding and maturation of substrates.^287–289^ Importantly, the key differences in the interactions of these same co-chaperones with HSP70 and HSP90 lie in their binding preferences. For example, FKBP51 and FKBP52 have a stronger preference for HSP90 than HSP70 does, whereas HOP and CHIP have a modest (approximately 2-folds) preference for HSP70.^290^

## Disease and function of molecular chaperones in humans

Protein homeostasis is fundamental to ensuring the functional integrity of the proteome. Nevertheless, under stressful conditions, proteins are prone to forming nonnative interactions. These proteins typically undergo misfolding and abnormal aggregation, which is harmful to the cell because this condition severely disrupts protein homeostasis.^291–293^ HSPs, as inherent molecular chaperone networks, regulate protein folding, aggregation, and degradation to ensure the functionality of the proteome. These proteins serve as the primary defense to maintain protein homeostasis by helping misfolded proteins to refold or degrade. HSPs exhibit significant functional diversity.^294,295^ Apart from guiding de novo folding and the refolding of misfolded proteins, they also regulate other important cellular processes, including protein trafficking, protein degradation, and the assembly of macromolecular complexes.^296^

**Fig. 4:**
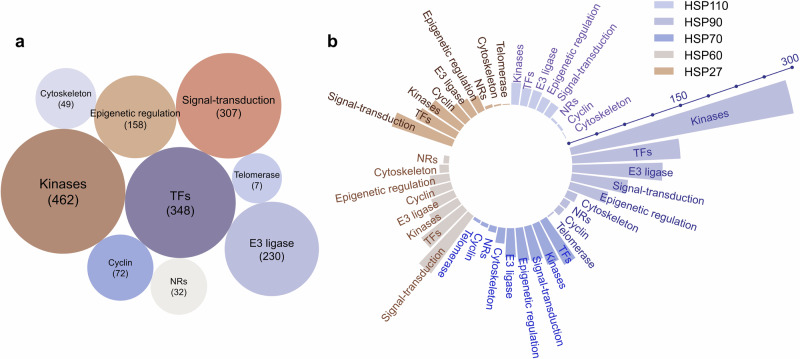
Analysis of client proteins regulated by HSPs. **a** All client proteins involved in different types, including kinases, transcription factors (TFs), E3 ubiquitin ligases (E3 ligases), nuclear hormone receptors (NRs), cytoskeletal proteins (cytoskeleton), signal-transduction proteins (signal-transduction), epigenetic regulatory proteins (epigenetic regulation), cyclin proteins (cyclin), and telomerase. **b** Analysis of client proteins regulated by different HSP family, including HSP110, HSP90, HSP70, HSP60 and HSP27. The data visual analytics of this figure used Charticulator

HSPs are widely distributed in various mammalian tissues and maintain protein homeostasis in all cellular compartments.^297,298^ While the expression levels of molecular chaperones are dynamic and may vary among tissues, they are typically found in diverse cell types, such as epithelial, muscle, and neuronal cells.^299^ Molecular chaperones can regulate a variety of client protein types. To understand the client protein spectrum of HSPs, we investigated and analyzed client proteins that can be regulated by HSPs on the basis of data from the BioGRID database (https://thebiogrid.org/). More than 1500 proteins have been reported as client proteins for HSPs. We classified different types of client proteins, including kinases, transcription factors, E3 ubiquitin ligases, nuclear hormone receptors, cytoskeletal proteins, signal-transduction proteins, epigenetic regulatory proteins, cyclin proteins, telomerase and other. Among them, kinases, transcription factors, and signal-transduction proteins have been extensively reported and studied, contributing the largest quantity of all identified client proteins (Fig. 4a). We further analyzed client proteins regulated by different HSP family members, including HSP110, HSP90, HSP70, HSP60 and HSP27 (Fig. 4b). We found that over 400 client proteins are regulated by HSP90 (also see http://www.picard.ch/downloads/HSP90interactors.pdf). Kinases constitute the most abundant group of HSP90 client interactors, followed by transcription factors and E3 ubiquitin ligases.^150^ The interaction of kinases with HSP90 has been used to determinate the specificity of kinase inhibitors in vivo.^300^ Among all the client proteins, the first HSP90 client protein identified was the viral kinase v-Src from viral sarcoma. It was subsequently revealed that HSP90 is crucial for the activation of steroid hormone receptors.^301–303^

**Fig. 5:**
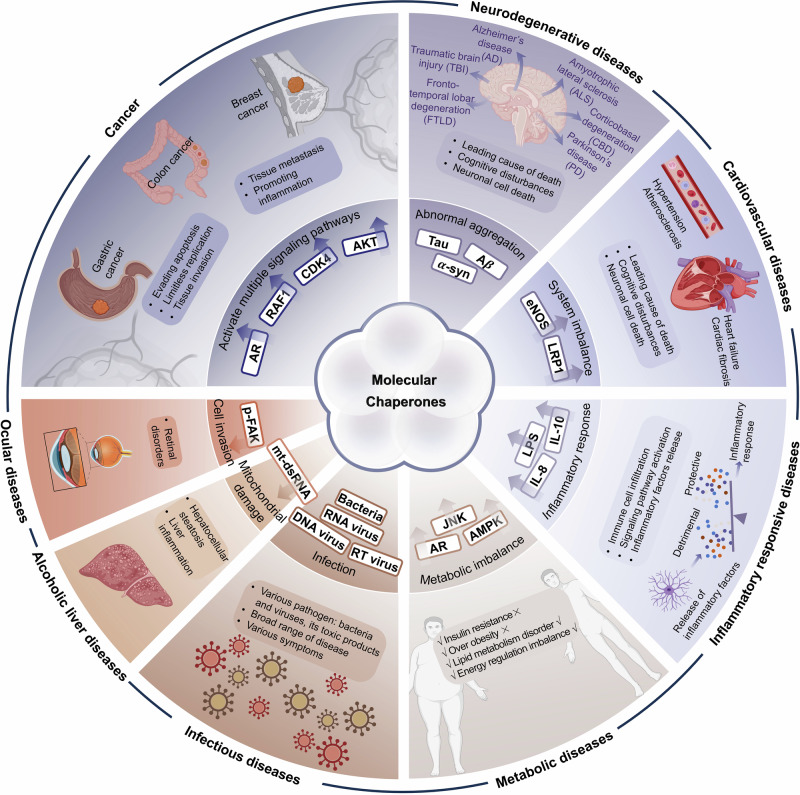
The regulatory functions of molecular chaperones in various diseases. The expression of HSPs is involved in many types of diseases. In different diseases, HSP family members play various regulatory roles. In cancer, multiple signaling pathways are activated through key substrate proteins of HSPs, such as AR, AKT, CDK4 and RAF1. In neurodegenerative diseases, tau tangles, Aβ deposits and α-synuclein appear to aggregate abnormally. HSPs can regulate the accumulation of these toxic aggregates. In cardiovascular diseases, the functions of eNOS and LRP1 can be regulated by HSPs, resulting in system imbalance and increasing the occurrence of related disease. In inflammatory responsive diseases, HSPs affect the release of inflammatory factors, such as LPS, IL-8 and IL-10, by immune cells. In metabolic diseases, HSPs are related to the insulin response and diabetic complications, resulting in metabolic imbalance through many pathways, such as AR, JNK and AMPK. In infectious diseases, HSPs facilitate (or inhibit in some cases) a wide range of viral and bacterial infectious diseases, including DNA viruses, RNA viruses and retroviruses (RT viruses). Ocular diseases and alcoholic liver diseases are also related to HSPs through p-FAK and mt-dsRNA respectively. The figure was created with BioRender.com

As HSPs play complex and precise roles in regulating numerous client proteins, they are related to various types of diseases. Cancer, neurodegenerative and cardiovascular diseases are the most closely related to molecular chaperones, and even rare diseases caused by genetic mutations were also reported to be involved in molecular chaperones. Importantly, the regulatory roles of molecular chaperone members in diseases are complex and diverse (Fig. 5). The complex regulatory effects of HSPs on disease arise from extensive substrate regulation, and involve different pathological conditions of the HSPs themselves. When tissues are under special stress, HSPs are upregulated and activated to exercise the function of self-defense.^304^ However, in many diseases, HSPs not only act as molecular chaperones to protect tissues, but also participate directly in cell survival or death by intervening in apoptotic signaling pathways, playing either inhibitory or promotional roles in different pathological situations.^15,305^ In addition, many reported PTMs of HSPs change their roles and thereby influence a myriad of cellular processes. For example, *S*-nitrosylation of HSP90 may cause severe disease phenotypes.^306–309^ Therefore, the regulatory role of HSPs under pathological conditions is highly complex, which poses a great challenge in targeting HSPs for drug design. Fortunately, the pathogenic mechanisms of HSPs in many diseases are gradually being elucidated, which offers hope for the design of drugs targeting HSPs. Many studies have also found that HSPs can serve as biomarkers for early-stage diseases, assisting in disease screening and prevention. They may also help with disease prognosis and monitoring treatment effectiveness. Therefore, this section introduces the probable regulatory functions of HSPs in various human diseases.

### Cancers

In cancer cells, metabolism and the microenvironment undergo significant changes, such as signal transduction pathway dysregulation and the upregulation of oncogene expression. Owing to these changes, HSPs are highly expressed in cancer cells to fold oncoproteins during tumor development, thereby maintaining cell function and survival.^310^ HSP90 is implicated in a number of pathologies. In 2005, the Lindquist group described the importance and future directions of the use of HSP90 as a therapeutic target in cancer. Later, they determined the nature and adaptive value of HSP90-contingent traits.^311,312^ Protein kinases, including AKT, cyclin-CDK4 and RAF1 kinases, are the most common client proteins of the HSP90 chaperone system (Fig. 5). AKT is involved in cell cycle arrest and apoptosis. A study showed that the expression of HSP90 is essential to keeping AKT stability. The inhibition of HSP90 in MCF-7 and SKBr-3 cells results in the degradation of AKT,^313^ further downregulating the activity of the PI3K/AKT pathway, which controls cell functions and many aspects of cell physiology.^314^ Similarly, inhibiting HSP90 expression decreases abundance of newly synthesized CDK4,^315^ which is a critical mediator of transition of cells to S phase and helps cancer cells to grow and survive.^316^ In addition, RAF1 kinase is associated with HSP90, an important part of the mitogen-activated protein kinase (MAPK) pathway that governs several crucial cellular processes. HSP90 has been shown to stabilize RAF1 and prevent it from undergoing 26S proteasome-mediated degradation,^317^ which promotes the activation of the MAPK pathway and the overproliferation and differentiation of cancer cells.^318^ Numerous studies indicated that the decrease of HSP90 expression can kill cancer cells by synergistically interfering with multiple signaling pathways; therefore, the inhibition of HSP90 is an effective therapeutic strategy against a range of tumor types, including leukemia, colon cancer, melanoma, breast cancer, ovarian cancer, and prostate cancer.^319^ Other HSPs, including HSP70, HSP60 and HSP27, are also considered to be closely related to cancers. The overexpression of HSP70 is involved in a variety of biological processes, promoting cell survival and contributing to the progression of many cancers. In prostate cancer, HSP70 can bind to AR, a nuclear transcription factor, and regulate AR activity (Fig. 5), which activates the AR signaling pathway and promotes the growth of prostate cancer cells.^320^ In addition, HSP60 and HSP27 have been reported to be promising biomarkers for prostate cancer screening to evaluate and monitor disease progression or recurrence.^321^

### Neurodegenerative diseases

Neurodegenerative diseases, such as Alzheimer’s disease (AD), are characterized by cognitive impairment. The hallmark of AD is the accumulation of amyloid-beta (Aβ) plaques and the formation of tau tangles.^322^ Extensive research has revealed that HSPs and their co-chaperones play pivotal roles in the folding and degradation of the hallmark proteins associated with neurodegenerative diseases (Fig. 5). The increased HSP60 and HSP70 expression in AD inhibits Aβ amyloid aggregation, protecting neurons from the intracellular accumulation of Aβ.^323,324^ HSP90 was also found to colocalize with tau tangles and Aβ deposits, regulating their aggregation and degradation. Chen et al. suggested that the neurotoxicity induced by Aβ can be mitigated through the use of HSP90 inhibitors.^325^ In addition, hyperphosphorylated tau is associated with the cognitive decline in patients with AD and similar tauopathies, and the hyperactivation of tau kinases is thought to contribute to tau pathogenesis.^326^ HSP90 inhibition greatly reduces the activity of tau and causes a decrease in phosphorylated tau. Parkinson’s disease (PD) is the second most common neurodegenerative disorder after AD and is characterized by the accumulation of toxic α-synuclein aggregates. HSP90 expression levels were found to be increased in the brains of PD patients. Additionally, HSP90 expression colocalizes with that of α-synuclein and is correlated with increased expression of insoluble α-synuclein. An in vitro study suggested that HSP90 protects against α-synuclein oligomer accumulation via its rapid transformation into fibrils, further preventing the formation of toxic α-synuclein.^327^ Spinocerebellar ataxia type 3 (SCA3) is a type of inherited neurological disease resulting from the expansion of polyglutamine (polyQ) via pathological mechanisms and is clinically characterized by peripheral neuropathy and cognitive disturbances.^328,329^ The expanded polyQ may destabilize many proteins, causing them to misfold and aggregate. HSP27, an important small HSP, is expressed in various cell types and tissues, where it promotes the refolding of denatured proteins.^330^ In the early stage of SCA3, HSP27 has been reported to reduce the accumulation and expansion of polyQ by increasing cellular antioxidant defense. In addition, HSP27 was found to inhibit the mitochondrial death pathway by binding to cytochrome C (Cyt C) and further inhibiting the formation of apoptosomes, which protects against cell apoptosis.^331^ HSPs is essential for the development of neurodegenerative diseases.

### Cardiovascular diseases

Cardiovascular disease (CVD) is a collective term that includes heart and vascular diseases, including hypertension, atherosclerosis, and cardiac fibrosis.^332^ Among these factors, arterial hypertension is linked to dysfunction of nitric oxide (NO), which is one of the main factors in keeping normal blood pressure.^333^ Numerous client proteins of HSP90, such as endothelial nitric oxide synthase (eNOS) that produces NO and participates in the relaxation of vascular smooth muscle, have been reported in known pathways involved in heart disease. Therefore, HSP90 interacts with eNOS and determines its correct folding, thereby regulating the function of NO and arterial hypertension (Fig. 5). In addition, the expression of HSP90 is up-regulated in the serum of patients with carotid atherosclerosis and in human atherosclerotic plaques. Atherosclerosis is a cardiovascular disease that develops in the intima of large- and medium-sized arteries and is usually caused by the deposition of lipids, especially low-density lipoprotein (LDL). LDL receptor-related protein 1 (LRP1), a HSP90 receptor, can bind various ligands and regulate the metabolism of LDL. HSP90 has also been found to bind to LRP1 and is related to ligands of LRP1 (Fig. 5). Therefore, HSP90 can affect the development of atherosclerosis through its influence on LDL metabolism. Besides, HSP90 regulates many pathways, including the MAPK, PI3K/AKT/mTOR, and tumor necrosis factor-alpha (TNF-α) signaling pathways, which regulate cardiovascular diseases via different pathological processes.^334^ In addition to the effect of HSP90, the highly complicated effect of HSP70 on CVD development is under discussion. Some studies have reported that HSP70 promotes arterial lipid accumulation and atherosclerotic lesion formation,^335^ and the expression of HSP70 is increased in advanced lesions of atherosclerotic plaques, which may indicate a protective effect of HSP70 stimulation.^336^ HSP70 expression in plasma is involved in the risk of acute coronary syndrome, and increased HSP70 mitigates damage in acute CVD.^337,338^ The functions of other HSPs in CVD have also been studied, and HSP27 overexpression has been shown to be protective against atherosclerosis, whereas the overexpression of HSP60 is atherogenic.^336^

### Inflammatory responsive diseases

The inflammatory response of the human body is involved in the regulatory processes of multiple immunological cells and molecules, in which the activation of T lymphocytes is vital. In autoimmune diseases, the activation of T lymphocytes can cause tissue inflammation and the production of autoantibodies.^339^ HSP70 has been shown to suppress disease in experimental models of autoimmunity. One group reported that T cell recognize HSP70 of *Mycobacterium tuberculosis* and terminate excessive inflammatory responses in a mouse model.^340^ Dendritic cells (DCs) are the most powerful specialized antigen-presenting cells and control the activation of T lymphocytes. HSP70 can downregulate CD86 and major histocompatibility complex (MHC) class II expression in DCs and further inhibits TNF-α production, which is essential for suppressing the activation of T lymphocytes. In addition, myeloid-derived suppressor cells (MDSCs) and monocytes are precursors of DCs and regulates inflammatory responses by affecting the level of anti-inflammatory cytokine IL-10, which is the major anti-inflammatory and immunosuppressive cytokine (Fig. 5). HSP70 can bind to endocytic receptors in MDSCs and monocytes and be endocytosed, resulting in the secretion of IL-10 and immunosuppression. The inhibition of cytokines related to the inflammatory response downregulates T lymphocyte activity and further prevents the activation, migration, and adhesion of inflammatory cells.^341^ In addition to studies on HSP70, the effects of HSP90 on inflammatory signaling pathways were reported in various models. The upregulation of HSP90 expression can activate the JAK/STAT pathway, which is closely related to cytokines and promotes the production of inflammatory mediators, such as IL-6.^342^ Recently, HSP90 was found to regulate lipopolysaccharide (LPS)-induced inflammation, in which HSP90 isoform inhibitors reduced the LPS-induced production of inflammatory mediators.^343^ HSP60 also has an interesting function in the inflammatory response. HSP60 can stimulate the maturation of inflammatory cells. The interaction between HSP60 and macrophages can induce the production of proinflammatory molecules. Therefore, certain anti-inflammatory HSP60-derived peptides are capable of acting as HSP60 antagonists and are useful for treating inflammatory diseases. In addition to acting as a modulator, HSP60 has been reported to be a biomarker of inflammatory diseases.^344^ Overall, the regulatory effects of HSPs on inflammatory-responsive diseases are complex and involve many various signaling pathways.

### Metabolic diseases

Metabolic diseases, including obesity and diabetes, are diseases or disorders that disrupt the normal process of converting food to energy at the cellular level.^345^ Type 2 diabetes mellitus (T2DM) is a metabolic disease that is closely linked to the epidemic of obesity. Insulin resistance, the inability of insulin-sensitive tissues to respond appropriately to insulin, is one of the primary causes of T2DM.^346^ Some studies have shown the important role of HSP70 in insulin resistance, which further leads to T2DM. Under stressful conditions, intracellular HSP70 increasingly migrates to the extracellular space and then enters the circulation. It is complex for HSP70 regulating obesity. HSP70 activates MyD88 and TIRAP pathways, which further activates c-Jun N-terminal kinase (JNK) that is responsible for insulin resistance (Fig. 5). JNK activation results in insulin resistance through multiple pathways.^347^ Conversely, the AMP-activated protein kinase (AMPK)-sirtuin 1 (SIRT1)-peroxisome proliferator-activated receptor gamma coactivator 1 alpha (PGC-1α) pathway is also a regulator of cellular energy status, which is vital for oxidative metabolism and mitochondrial biogenesis. One study revealed that increased HSP70 expression can increase AMPK activity, lead to considerable upregulation of SIRT1, and eventually activate PGC-1α. Activated PGC-1α triggers mitochondria and induces fatty acid oxidative processes, which reduce the accumulation of free fatty acids and reduce insulin resistance.^348^ Overall, the complicated role of HSP70 in T2DM is closely associated with insulin resistance pathogenesis, suggesting that HSP70 has vital therapeutic potential in the management of T2DM. As co-chaperones of HSP70, HSP40 family also displays crucial function in metabolic diseases associated with insulin resistance. For example, increased expression of DNAJC15 was found in obese boys and was related to increased insulin resistance.^349^ Some studies have shown function of DNAJB3 in increasing insulin sensitivity and glucose uptake.^350^ Metabolic diseases require the involvement of various regulatory factors, such as adiponectin. Adiponectin is an adipokine, secreted by adipocytes. It regulates the progression of insulin resistance, diabetes, and diabetic complications. HSP60 was recently proved to interact with and stabilize the adiponectin receptor, mediating adiponectin signaling in vitro. Therefore, a reduction in HSP60 expression can inhibit adiponectin activity and protect against diet-induced obesity.^351,352^

### Infectious diseases

Infectious diseases can be defined as illness caused by a pathogen, including bacteria and viruses, or its toxic product. Viral infections lead to different diseases that are closely related to the abnormal regulation of HSPs. These proteins play crucial roles at multiple stages of the viral infection process, including virus entry, replication, assembly, and release. HSP90, HSP70, HSP60, HSP40, and HSP27 were found to be actively related to the entry, replication and uncoating phases of viral infection. Besides, HSP40 and HSP70 are essential for viral gene expression and viral assembly. HSP90 contributes to the import and export of viruses from the nucleus.^353^ For example, the RNA virus enterovirus A71 (EV-A71) depends on HSP90 for entry.^354^ In addition, HSC70 was found to regulate the activity of the internal ribosome entry site and serve as an antiviral target of EV-A71 infection.^355^ In recent studies, HSP27 was also demonstrated to regulate EV-A71 infection through nuclear translocation and viral replication.^356,357^ Recent studies have shown that the recognition and invasion of the RNA virus SARS-CoV-2 depend on GRP78.^358^ Infection with the DNA virus hepatitis B virus (HBV) induces reactive oxygen species production and may affect hepatocellular carcinoma. HSP90 was reported to interact with HBV core protein dimers and its reverse transcriptase, obstructing the replication and assembly of HBV.^359–361^ Additionally, HSP40, HSP60 and HSP70 are related to the replication and immune modulation of HBV infection.^362–366^ In addition to RNA and DNA virus, the infection of retrovirus also poses a great threat to human health. The entry, gene expression and genome integration of human immunodeficiency virus type 1 (HIV) need the involvement of HSP40, HSP70 and HSP60.^367–369^ Unlike the treatment of viral infections, which often involves targeting HSPs in host cells, the treatment of bacterial infections focuses on targeting HSPs within the bacteria themselves. Bacterial HSPs are highly conserved among pathogens.^370^ These proteins play critical roles in bacterial physiology by interacting with proteins of multiple signaling pathways, which is essential in the overall virulence of these organisms.^371^ For example, in *Candida species*, HSPs contribute to resistance against antifungal drugs by modulating signaling pathways. Additionally, HSPs are responsible for mediating stress responses, are related to morphogenesis, and regulate diverse cellular processes such as biofilm formation.^372^ Given these roles, targeting HSPs represents a promising strategy for developing new treatments for *C. albicans* infections.^373^ In conclusion, HSPs are promising targets for infectious diseases because of their crucial roles in bacterial and viral infections, although their exact mechanisms are not clear and urgently need to be determined.

### Other diseases

HSPs work as an integrated network that participates in cell signal transduction, the cell cycle, and apoptosis regulation. Apart from the seven categories of diseases listed above, HSPs are involved in regulating many other diseases, including ocular diseases, liver diseases and skin diseases.^15^ The retina is a complex, metabolically demanding and often stressed eye component.^374^ HSP90 is widely distributed throughout the retina and plays multiple roles in the folding, activation and assembly of many proteins.^375^ Uveal melanoma (UM) is the most frequent early intraocular malignancy in adults. Focal adhesion kinase (FAK), a cytoplasmic tyrosine kinase, promotes cellular migration, invasion and metastasis. As a client of HSP90, the stability and function of FAK is closely related to HSP90. HSP90 inhibition interferes with the phosphorylation of FAK, stimulating its proteasome-mediated degradation, which ultimately decreases the invasion of cancer across pathways mediated by FAK. In addition to FAK, overexpression of phosphorylated AKT (p-AKT) are related to an increased risk of metastatic disease in UM patients (Fig. 5). HSP90 inhibitors were reported to decrease p-AKT levels in UM cell lines, resulting in cell death. HSP90 has the ability to induce dual response in the retina, thereby improving visual function and photoreceptor survival.^376^ Liver diseases are caused by many factors, including viruses, ethanol, drugs, and autoimmune abnormalities. Nonalcoholic fatty liver disease (NAFLD) is a common liver disease that may lead to cirrhosis.^377^ The key factors in liver disease involve mitochondrial dysfunction and the release of mitochondrial damage-associated molecular patterns. dsRNA of mitochondrial origin (mt-dsRNA), an important mitochondrial damage-associated molecular pattern, can provoke a proinflammatory response in an alcoholic liver disease model (Fig. 5).^378^ HSP60 is found primarily in the mitochondrial matrix, and its folding activity can protect mitochondria from proteotoxicity under stress.^379^ The overexpression of HSP60 inhibits the release of mt-dsRNA, which further inhibits the activation of toll-like receptor 3 (TLR3), melanoma differentiation-associated gene 5 (MDA5), and phosphorylated interferon regulatory factor 3 (p-IRF3). Reducing the activity of the TLR3/MDA5/p-IRF3 signaling pathway may improve hepatocellular steatosis and liver inflammation. HSP60 is a promising drug target for improvement of NAFLD and may provide novel insight into its hepatoprotective effect in various liver diseases.^380^

## Targeting therapy of molecular chaperone

During the past two decades, over 30 inhibitors targeting molecular chaperones have been entered into clinical trials as reported by clinicaltrials.gov, which were mainly contributed by HSP90 ATPase inhibitors. The reasons for the majority of HSP90 inhibitors in clinical trials are related to their central roles in cell biology and their potential broad application in tumor therapy. Numerous clients rely on HSP90 for maturation, activation, and stabilization. HSP90 is overexpressed in various tumor cells, leading to the increased expression of oncogenic proteins and aberrant activation of signaling pathways, which often drive tumorigenesis and progression. Besides, HSP90 functions not only within cells but is also continuously secreted outside tumor cells. eHSP90 have been found to show a positive correlation with tumor malignancy.^381,382^ Therefore, in contrast to other HSPs, in-depth understanding of function of HSP90 facilitates the development of HSP90 inhibitors, making inhibiting HSP90 an attractive strategy for disease therapy. HSP90 inhibitors have shown potential in the treatment of many types of cancers, including breast, lung, and liver cancers. Moreover, the application of HSP90 inhibitors is not limited to cancer treatment but also includes other diseases such as neurodegenerative and immune system disorders, further increasing their research and development interest.^383,384^ In early clinical trials, many inhibitors were mainly used as single agent in various cancer types. However, limited clinical activity significantly restricted their therapeutic application. At high dosage concentrations, HSP90 inhibitors affected the stability of multiple intracellular proteins and induced HSR, leading to hepatotoxicity and ocular toxicity.^385,386^ However, at low dosage concentrations, HSP90 inhibitors have limited anticancer effects.^381,387^ Therefore, to overcome the drug-related toxicity and limited clinical effects, most inhibitors have been tested in combination with various anti-cancer therapies, including chemotherapy drugs, radiation, and immunotherapy.^388^ The combination drug therapies achieved synergistic effects, and low drug resistance, improved clinical outcomes and prognosis of patients were observed in many clinical trials. An HSP90 inhibitor, ganetespib, has been used in combination with crizotinib in clinical research, showing an enhanced inhibitory effect on anaplastic lymphoma kinase (ALK) positive lung cancers.^389,390^ However, the combination drug therapies of these inhibitors also carried potential risks, including uncertain drug interactions, increased toxicity, and complex treatment management.^391,392^ Therefore, currently, no drugs targeting molecule chaperones have received FDA approval.

Considering the challenges in clinical trials, there is a need for the development of novel strategies targeting molecule chaperones to discover drugs with enhanced activity and reduced toxicity. Research in preclinical trials mainly focused on developing novel HSP90s and HSP70s inhibitors, which could be classified into four development stages, classify them into four development stages: stage 1, which targets pan-isoforms of the HSP families (from 1990s); stage 2, which targets isoforms of the HSP families with high selectivity (from 2000s); stage 3, which targets PPIs between HSPs and co-chaperones (from 2010s); and stage 4, which involves the design of multi-specific molecules based on HSPs (from 2020 s). Gradually, the roles of more HSPs family members in disease regulation were elucidated, and they were also considered as potential targets for developing drugs, including HSP110, HSP100, HSP60 and sHSPs. Therefore, it is possible that more inhibitors will enter clinical trials by targeting molecular chaperone systems with diverse mechanisms and eventually become effective drugs for diseases.

### Inhibitors in clinical trials

**Fig. 6:**
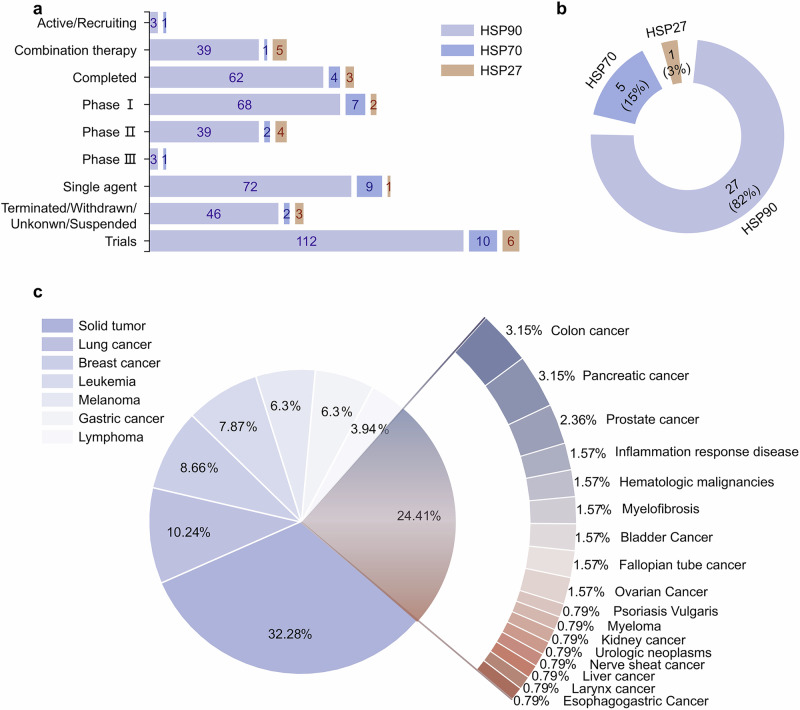
Analysis of inhibitors targeting HSPs in clinical trials. **a** Overview of HSPs inhibitors in clinical trials. The results are obtained by searching in clinicaltrials.gov. using different HSP members as keywords. **b** Analysis of regulators targeting different HSP members. A total of 33 HSPs regulators have been tested clinical trials. Among them, HSP90 inhibitors made up the majority, occupying 82% (27), and regulators targeting sHSPs and HSP70s respectively occupied 15% (5) and 3% (2). There are no regulators targeting other HSP members entered into clinical trials. **c** Analysis of conditions for all clinical trials. In addition to inflammation response disease (1.57%) and psoriasis pulgaris (0.79%), the conditions of these clinical trials most are cancers, approximately contributing 98%, including many types of cancers. The data visual analytics of this figure used Charticulator

Currently, a total of 33 HSPs regulators have been studied in 128 clinical trials, as reported by clinicaltrials.gov. 54% have been completed, and 40% have been suspended, terminated, withdrawn or unknown. Only 6% are currently active or on recruiting stage. There are, respectively, 77, 45 and 4 in Phase I, Phase II and III clinical trials (Phase I/II and Phase II/III respectively categorized as Phase II and Phase III), while none was approved by FDA due to issues of safety and efficacy (Fig. 6a). Whether used as single agents (65%) or combination therapy (35%), the conditions of these clinical trials most are cancers, approximately contributing 98%. In addition to applied to solid tumors (occupying 32.28%), these clinical trials involved in many cancers, including lung cancer (occupying 32.28%), breast cancer (occupying 8.66%), melanoma (occupying 6.3%), gastric cancer (occupying 6.3%), lymphoma (occupying 3.94%), pancreatic cancer (occupying 3.15%) and other types of cancers (Fig. 6c). Among all regulators entering clinical trials, HSP90 inhibitors made up the majority, occupying 82% (27), and regulators targeting sHSPs and HSP70s respectively occupied 15% (5) and 3% (2). In addition, there are no regulators targeting other HSP members entered into clinical trials (Fig. 6b). Therefore, this part mainly focuses on the limitations and challenges faced by HSP90 inhibitors (Table 2).

**Table 2:** Clinical trials of targeting HSP90 ATPase inhibitors

Name	Adjunctive agents	Conditions	Phase	Status	First Posted	NCT number
17-AAG	/	ZAP-70 Positive B-Cell Chronic Lymphocytic Leukemia	I	Terminated	2005	NCT00319930
	/	Relapsed or Refractory Anaplastic Large Cell Lymphoma, Mantle Cell Lymphoma, or Hodgkin’s Lymphoma	II	Completed	2005	NCT00117988
	/	Advanced Solid Tumors or Non-Hodgkin’s Lymphoma	I	Terminated	2003	NCT00019708
	/	Relapsed or Refractory Solid Tumors or Leukemia	I	Completed	2004	NCT00079404
	Sorafenib tosylate	Unresectable or Metastatic Solid Tumors	I	Completed	2005	NCT00121264
17-DMAG	/	HER2 Positive Breast Cancer	II	Terminated	2008	NCT00780000
	/	Chronic Lymphocytic Leukemia, Small Lymphocytic Lymphoma, B-Cell Prolymphocytic Leukemia	I	Terminated	2010	NCT01126502
	Trastuzumab and Paclitaxel	Breast Cancer or Solid Tumor	I	Completed	2008	NCT00803556
IPI-504	/	Lung Cancer, Stage IIIb Lung Cancer, Stage IV Lung Cancer,	II	Terminated	2010	NCT01228435
	Docetaxel	Non-small Cell Lung Cancer	II	Completed	2011	NCT01362400
IPI-493	/	Hematologic Malignancies	I	Terminated	2010	NCT01193491
	/	Advanced Malignancies	I	Terminated	2008	NCT00724425
AT13387	/	Solid Tumors, Breast Cancer	I	Completed	2010	NCT01246102
	/	Metastatic Solid Tumors	I	Completed	2009	NCT00878423
	Crizotinib	Non-Small Cell Lung Cancer	I/II	Completed	2012	NCT01712217
	Abiraterone Acetate	Prostate Cancer	I/II	Completed	2012	NCT01685268
STA-9090	/	Solid Tumors	I	Completed	2008	NCT00687934
	Docetaxel	Solid Tumor Malignancies	I	Completed	2010	NCT01183364
BIIB021	/	Gastrointestinal Stromal Tumors	II	Completed	2008	NCT00618319
	/	Advanced Solid Tumors	I	Completed	2008	NCT00618735
	Antacid	Advanced Solid Tumors	I	Completed	2009	NCT01017198
	Exemestane	Hormone Receptor Positive Metastatic Breast Cancer	II	Completed	2009	NCT01004081
BIIB028	/	Advanced Solid Tumors	I	Completed	2008	NCT00725933
PU-H71	/	Primary Myelofibrosis, Post-Polycythemia Vera Myelofibrosis, Post-Essential Thrombocythemia Myelofibrosis	I	Terminated	2019	NCT03935555
	/	Solid Tumors and Low-Grade Non-Hodgkin’s Lymphoma	I	Terminated	2012	NCT01581541
	/	PET Imaging of Cancer Patients	Early I	Active, not recruiting	2011	NCT01269593
MPC-3100	/	Refractory or Relapsed Cancer	I	Completed	2009	NCT00920205
Debio 0932	/	Cancer, Neoplasms, Solid Tumors, Lymphoma	I	Completed	2010	NCT01168752
	**/**	Psoriasis Vulgaris	I	Unknown	2018	NCT03675542
XL888	/	Solid Tumors	I	Terminated	2008	NCT00796484
	Vemurafenib	Melanoma	I	Completed	2012	NCT01657591
	Vemurafenib and Cobimetinib	Melanoma	I	Active, not recruiting	2016	NCT02721459
SNX-5422	/	Solid Tumor Malignancy, Lymphoid Malignancy, Leukemia, Lymphoma	I	Completed	2008	NCT00647764
	/	Solid Tumor Malignancies	I	Completed	2007	NCT00506805
	/	HER2 Positive Cancers.	I/II	Terminated	2013	NCT01848756
	Ibrutinib	Chronic lymphocytic leukemia	I	Withdraw	2016	NCT02914327
TAS-116	Palbociclib	Breast and Rb-null Cancer	I	Recruiting	2022	NCT05655598
	Imatinib and Sunitinib	Gastrointestinal Stromal Tumors	I	Recruiting	2022	NCT05245968
	/	Solid Tumors	I	Completed	2016	NCT02965885
HSP990	/	Advanced Solid Tumors	I	Terminated	2010	NCT01064089
	/	Advanced Solid Tumors	I	Completed	2009	NCT00879905

The results are obtained by searching in clinicaltrials.gov. using different HSP members as keywords. Data were last updated on July 25th, 2024

Initial efforts to develop HSP90 inhibitors focused on inhibition of the ATPase activity of HSP90 by competitively binding to the ATPase site of NTD. Based on the main scaffolds, HSP90 ATPase inhibitors include four representative classes: the first-generation ansamycin-based inhibitors, the second-generation resorcinol-based inhibitors, the third-generation purine-based inhibitors and the fourth-generation benzamide-based inhibitors (Table 2).

The first-generation HSP90 ATPase inhibitors derived from geldanamycin (GDA). In 1997, natural product GDA was confirmed as the first HSP90 ATPase inhibitor, belonging to ansamycin-based compounds.^381^ The affinity for HSP90 of GDA was 1215 nM, which led to the effective inhibition of HSP90 and demonstrated potential anticancer effects via targeting the N-terminal ATP binding pocket of HSP90 (Table 3).^393^ However, low solubility and high reactivity of 17-MeO group of GDA made GDA unsuitable for drug development. Skeleton-based structural modifications of GDA promoted the discovery of the first-generation ansamycin-based HSP90 ATPase inhibitors, including representative 17-AAG, 17-DMAG, IPI-504 and IPI-493 (Table 2). 17-AAG is the first HSP90 inhibitor entered into clinical studies. 17-AAG retained activity of GDA. Originally, 17-AAG was used as single agent therapy to treat relapsed or refractory solid tumors, lymphoma, and various types leukemia. However, most clinical trials were halted at Phase I or II due to poor therapeutic effects. Failing to work was related to several factors, such as suboptimal doses in order to avoid treatment-related toxicities. Subsequently, 17-AAG was explored to treat many unresectable or metastatic solid tumors by combination therapy strategies and showed antitumor effects. Among these, 17-AAG and cisplatin exhibited a synergistic inhibitory effect on the lung adenocarcinoma, and this combination therapy exhibits stronger inhibition at lower doses. And the combination of 17-AAG with sorafenib also showed clinical activity in patients with unresectable or metastatic solid tumors. However, the toxicity of 17-AAG in these trials was found to increase gradually with dose and schedule.^394^ In addition to diarrhea and fatigue, severe hepatotoxicity of 17-AAG posed a prominent and inevitable challenge. Besides, 17-AAG showed poor water solubility, short half-life, and uneven distribution in the body, greatly limiting its therapeutic efficacy and safety.^395–397^ To improve drawbacks of 17-AAG, 17-DMAG was obtained with improved solubility and bioavailability.^398^ 17-DMAG was also studied as a single agent or in combination against various cancer types in many clinical trials. Excitingly, 17-DMAG demonstrated with active results against refractory human epidermal growth factor receptor 2-positive (HER2^+^) metastatic breast cancer and myelogenous leukemia. However, the higher toxicity of 17-DMAG, in comparison to 17-AAG, led to the discontinuation of its clinical trials.^394,399^ Other soluble stabilized hydroquinone forms of 17-AAG, IPI-504 and its oral formulation IPI-493 were developed by Infinity Pharmaceuticals, Inc.^394^ Although clinical studies indicated that the combination therapy of IPI-504 and docetaxel delayed the progression of non-small cell lung cancer (NSCLC), IPI-504 showed higher than expected hepatic toxicity. Clinical trials of IPI-493 against malignancies were also terminated due to low drug exposure.^400^

**Table 3:** Preclinical studies of targeting HSP90

Compound	Structure	Activity	Mechanism (Sites)
Geldanamycin		K_d_ = 1215 nM	Pan-HSP90 inhibitor (NTD)
Radicicol		K_d_ = 19 nM	Pan-HSP90 inhibitor (NTD)
HP-4		^a^IC_50_ = 18 nM	Pan-HSP90 inhibitor (NTD)
PU-3		^b^IC_50_ = 15–20 μM	Pan-HSP90 inhibitor (NTD)
SNX-2112		^a^IC_50_ = 3.0 nM	Pan-HSP90 inhibitor (NTD)
Compound 30 f		^b^IC_50_ = 5.3 nM	Pan-HSP90 inhibitor (NTD)
XL888 derivative		^a^IC_50_ = 57 nM	Pan-HSP90 inhibitor (NTD)
Capsaicin		/	Pan-HSP90 inhibitor (NTD)
Novobiocin		^c^IC_50_ = 256 μM (MCF-7 cells)	Pan-HSP90 inhibitor (CTD)
Noviose sugar surrogates		^c^IC_50_ = 560 nM (SKBr3 cells)	Pan-HSP90 inhibitor (CTD)
TVS21 derivative		^c^IC_50_ = 3.1 μM (MCF-7 cells)	Pan-HSP90 inhibitor (CTD)
Compound 2n		^c^IC_50_ = 9.5 μM (SKBr3 cells)	Pan-HSP90 inhibitor (CTD)
Deguelin		^c^IC_50_ = 110 nM (H1299 cells)	Pan-HSP90 inhibitor (CTD)
HVH-2930		K_d_ = 97 µM	Pan-HSP90 inhibitor (CTD)
Penisuloxazin A		/	Pan-HSP90 inhibitor (CTD)
Enniatin A		/	Pan-HSP90 inhibitor (MD)
Diptoindonesin G		K_d_ = 0.13 μM	Pan-HSP90 inhibitor (MD)
DN401		TRAP-1: K_d_ = 79 nM; HSP90α/β: K_d_ = 698 nM	TRAP-1 selective inhibitor (NTD)
SMTIN-P01		/	TRAP-1 selective inhibitor (NTD)
NECA		GRP94: K_d_ = 0.53 μM; HSP90α: K_d_ = 46 μM	GRP94 selective inhibitor (NTD)
PU-H39		GRP94: ^a^IC_50_ = 2.4 μM; HSP90α/β: ^a^IC_50_ = > 500 μM	GRP94 selective inhibitor (NTD)
BnIm		GRP94: ^a^IC_50_ = 1.2 μM; HSSP90α: ^a^IC_50_ = 1.4 μM	GRP94 selective inhibitor (NTD)
KUNG65		GRP94: K_d_ = 0.54 μM; HSP90α: K_d_ = 39 μM	GRP94 selective inhibitor (NTD)
ACO1		GRP94: K_d_ = 0.44 μM; HSSP90α: K_d_ = > 100 μM	GRP94 selective inhibitor (NTD)
DDO-5813		GRP94: ^a^IC_50_ = 2.0 nM; HSP90α: ^a^IC_50_ = > 100 μM	GRP94 selective inhibitor (NTD)
EC144		HSP90: K_i_ = 0.20 nM; TRAP1: K_i_ = 255 nM; GRP94: K_i_ = 61 nM	HSP90α/β selective inhibitor (NTD)
Compound 31		HSSP90α/β: K_i_ = 5.0 nM; GRP94: K_i_ > 10 μM; TRAP1: K_i_ > 10 μM	HSP90α/β selective inhibitor (NTD)
Gambogic acid		HSP90β: K_d_ = 33 nM; HSSP90α: K_d_ = 195 nM	HSP90β selective inhibitor (MD)
Corylin		HSP90β: K_d_ = 24.7 nM	HSP90β selective inhibitor (MD)
KUNB31		HSP90β: K_d_ = 0.18 nM GRP94: K_d_ = 8.5 μM HSP90α: K_d_ = 9.6 μM	HSP90β selective inhibitor (NTD)
KUNA-111		HSP90α: ^a^IC_50_ = 0.25 μM; HSP90β: ^a^IC_50_ = 3.8 μM	HSP90α selective inhibitor (NTD)
KUNA-115		HSP90α: ^a^IC_50_ = 0.84 μM; HS90β: ^a^IC_50_ = >50 μM	HSP90α selective inhibitor (NTD)
NDNA4		eHSP90α: ^b^IC_50_ = 0.34 μM	eHSP90 selective inhibitor (NTD)
DMAG-N-oxide		/	eHSP90 selective inhibitor (NTD)
STA-12-7191		/	eHSP90 selective inhibitor (NTD)
Celastrol A		^c^IC_50_ = 3.0 μM (Panc-1 cells)	HSP90-CDC37 PPIs Inhibitor (NTD)
Pep-1	KHFGMLRRWDD	K_d_ = 7.1 μM	HSP90-CDC37 PPIs Inhibitor (NTD)
DDO-59120	EKYEKQIK	K_d_ = 6.9 μM	HSP90-CDC37 PPIs Inhibitor (NTD)
DDO-5936		HSP90: K_d_ = 7.4 μM	HSP90-CDC37 PPIs Inhibitor (NTD)
DCZ3112		^c^IC_50_ = 7.9 μM (SK-BR-3 cells); ^c^IC_50_ = 4.6 μM (BT-474 cells)	HSP90-CDC37 PPIs Inhibitor (/)
HAM-1		K_d_ = 24 μM	HSP90-Aha1PPIs Inhibitor (NTD)
CP9		/	HSP90-p23 PPIs Inhibitor (NTD)
Cucurbitacin D		^c^IC_50_ = 0.60 μM (MCF-7 cells)	HSP90-p23 PPIs Inhibitor (/)
Y-632		/	HSP90-HOP PPIs Inhibitor (/)
SM145		/	HSP90-FKBPs PPIs Inhibitor (NTD, MD)
RNK05028		/	Multi-specific molecules (NTD)
HEMTAC 26		CDK4/6: DC_50_ = 26/19 nM	Multi-specific molecules (NTD)
BP3		^c^IC_50_ = 0.60 μM (MCF-7 cells)	Multi-specific molecules (NTD)
X10g		HSP90β: DC_50_ = 0.3 μM	Multi-specific molecules (NTD)
lw13		/	Multi-specific molecules (NTD)

*K*_*d*_ target binding affinity, *K*_*i*_ target binding affinity, ^*a*^*IC*_*50*_ ATPase inhibitor activity, ^*b*^*IC*_*50*_ competitive binding activity, ^*c*^*IC*_*50*_ anti-proliferative activity, *DC*_*50*_ protein degradation activity, “/ “indicates no clear reports

Radicicol (RDC) is another natural product inhibitor that opened up the design of the second-generation resorcinol-based HSP90 inhibitors. The resorcinol portion of RDC formed a hydrogen-bonded network with residues of N terminal ATP binding pocket of HSP90, which primarily facilitated the high affinity with K_d_ values of 19 nM (Table 3).^393,401^ However, it shows no efficacy due to the unstability of allylic epoxide and unsaturated ketones, resulting in the rapid metabolism in vivo. To improve the stability and antiproliferative activity, significant effort has been dedicated and different series of derivatives have been obtained. Of these, compound AT-13387 and STA-9090 (Table 2) was pushed into clinical studies because of their increased affinity, appropriate aqueous solubility and other better drug-like properties.^402–404^ Notably, the damaging hepatotoxicity of the first-generation ansamycin-based HSP90 inhibitors was not observed in their clinical trials. However, AT-13387 was found to non-selectively degrade a large number of substrates, further inducing HSR that is characterized by the over-expression of other HSPs, such as HSP70. HSR can enable cancer cells to activate a protective mechanism, resulting in the ineffectiveness of HSP90 ATPase inhibitors. Therefore, HSR is a key cause for HSP90 ATPase inhibitors lacking therapeutic activity against cancers in many clinical trials. Two Phase I/II trials, AT-13387 in combination with abiraterone acetate against prostate cancer and AT-13387 in combination with crizotinib against NSCLC, showed insufficient clinical activity in patients.^405,406^ Based on that, further exploration of AT-13387 and related combinations was also not pursued. Though the clinical treatment outcome of STA-9090 showed modest enhancement compared to AT-13387, poorly tolerated and gastrointestinal toxicity was also observed.^407^ Besides, serious adverse reaction occurred frequently during the treatment period, which greatly limited the clinical application of the second-generation HSP90 inhibitors.^408^

The development of the third-generation purine-based inhibitors was enlightened by ATP. PU-3 is the first discovered inhibitor. Similar to GDA and RDC, the purine moiety of PU-3 forms the hydrogen interaction with the key residue of N terminal ATP binding pocket. Therefore, PU-3 can compete with GDA for binding to HSP90 with EC_50_ of 15-20 μM (Table 3).^409,410^ Among the types of inhibitors, many derivatives have entered clinical trials. The results suggested that these derivatives showed some desirable properties compared to the first-generation HSP90 inhibitors, including improved water solubility and lowered susceptibility.^411^ BIIB021 was the first purine-based HSP90 inhibitor to enter clinical trials (Table 2).^412^ Among them, a Phase II clinical study indicated that BIIB021 could alter the metabolic activity of gastrointestinal stromal tumors (GIST), without substantial hepatotoxicity observed. However, BIIB021 need high doses to exhibit antitumor efficacy. Subsequently, BIIB028 was obtained by structural optimization based on BIIB021, which was also confirmed to exhibit HSP90 inhibitory activity and objective responses in advanced solid tumors in a clinical trial.^413–415^ PU-H71 is a distinct purine-based inhibitor, with an endogenous iodine atom that has been conveniently replaced with the positron emission tomography (PET) radionuclide ^124^I, leading to ^124^I-PU-H71 as an imaging agent in tumors.^416^ Besides, PU-H71 has been used to treat various cancers, but most of its clinical trials remained in Phase I. The safety and efficacy of PU-H71 still need to be assessed (Table 2).^417^ MCP‑3100 and Debio0932, also entered into the clinical research stage, while failed to move beyond phase II (Table 2).^418^ In addition, Debio0932 has recently been found to exhibit anticancer activity in in neuroblastoma cell line, which shows more possibilities for Debio0932 in the treatment of neuroblastoma.^419^

The last representative class of HSP90 ATPase inhibitors is the benzamide-based class. In 2004, Gu XJ et al. identified novel HSP90 inhibitors and obtained two hits with HSP90 inhibitory activity.^420^ Structure modification based on the hits as starting points led to the discovery of XL888 (Table 2) that has improved potency and pharmacokinetic properties, and thus entered into clinical trials.^421^ In addition to evaluated in solid tumors as a single agent, XL888 also was used to treat unresectable melanoma in combination with vemurafenib and cobimetinib in phase I trials. The results showed therapeutic effect of XL888 against melanoma with controllable adverse reactions.^422^ However, the clinical development of XL888 was halted at the phase I, without significant progress. Subsequently, SNX-2112 was discovered, guided by computational chemistry and crystal structures, which showed excellent affinity for HSP90 with IC_50_ of 3.0 nM (Table 3).^423^ And its oral form (SNX-5422) also entered into clinical trials (Table 2), due to delaying growth excellent antiproliferative activity in an HT-29 mouse model.^424^ Currently, multiple phase I clinical trials related to SNX-5422 have been completed, involved in many tumors including HER2^+^ breast cancer, refractory solid tumor malignancies and lymphomas. Although initial activity of SNX-5422 has been confirmed, its ocular toxicity has been observed in patients, obstructing further investigation of SNX-5422 as a single agent.^425,426^ As a combination therapy strategy, SNX-5422 was used in combination with ibrutinib to reverse its resistance in patients with chronic lymphocytic leukemia.^427^ However, none clinical trials of SNX-5422 have advanced beyond the Phase II currently.

Currently, there are various HSP90 ATPase inhibitors in the clinical research stage, including the four classic structural analogs mentioned above and the novel skeleton inhibitors discovered in recent years. For example, HSP990, a novel HSP90 inhibitor with aminopyrimidine structure, exhibited effective anti-cancer actions and entered clinical trials for advanced solid tumors (Table 2).^166,428,429^ These inhibitors are designed to improve therapeutic efficacy and lower toxicity by leveraging diverse chemical structures and metabolic properties. However, there are still no FDA-approved drugs targeting HSPs. Recent research has also focused on the selectivity of HSP90 inhibitors for different isoforms. TAS-116 is the representative HSP90α/β selective inhibitor, derived from SNX2112,^430,431^ which realized the affinity for HSP90α/β (K_i_ = 21.3/34.7 nM), over 1400-folds selective compared to GRP94 and TRAP-1.^432,433^ Notably, TAS-116 (trade name Jeselhy) have been approved by Japan in 2022, for the treatment of chemotherapy-resistant GIST (Table 2).^434^ The first approval represents significant progress in the development of targeting molecular chaperones as therapeutic drugs.

### Inhibitors on preclinical trials targeting molecular chaperones

#### Targeting therapy of HSP90

##### Stage 1: targeting pan-HSP90 isoforms (from 1990s)

**Novel HSP90 NBD ATPase inhibitors**

**Fig. 7:**
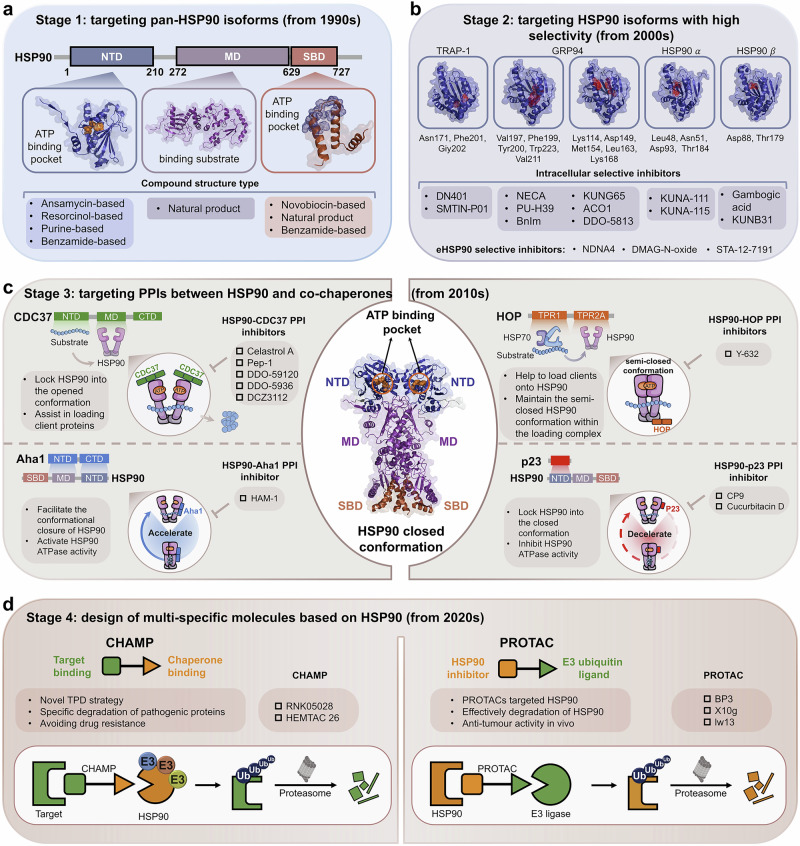
Targeting therapy of HSP90. **a** Stage 1: targeting pan-HSP90 isoforms (from 1990s), including HSP90 NBD ATPase inhibitors, MD inhibitors and CTD inhibitors. **b** Stage 2: targeting HSP90 isoforms with high selectivity (from 2000s). Selective binding sites of HSP90 isoforms have been respectively reported, leading to discovery of inhibitors with high selectivity, including TRAP-1 selective inhibitors, GRP94 selective inhibitors, HSP90β selective inhibitors and HSP90α selective inhibitors. Besides, eHSPs selective inhibitors have also been designed by introducing impermeable membrane moieties into HSP90 inhibitors. **c** Stage 3: targeting PPIs between HSP90 and co-chaperones (from 2010s), including HSP90-CDC37 PPI inhibitors, HSP90-HOP PPI Inhibitors, HSP90-Aha1 PPI Inhibitors and HSP90-P23 PPI inhibitors. **d** Stage 4: design of multi-specific molecules based on HSP90 (from 2020s), including CHAMPs and PROTACs

Due to limited clinical application of current HSP90 inhibitors, it is imperative to discover novel HSP90 ATPase inhibitors with increased potency and reduced toxicity (Fig. 7a, Table 3). In 2014, our group found novel compounds with HSP90 ATPase inhibition based on AT-13387. Several analogs were obtained with improved HSP90 inhibitory activity, positive antitumor activity and good physicochemical properties, following by comprehensive structure-activity relationship (SAR) study.^435,436^ Besides, HP-4 is the latest reported resorcinol-based HSP90 inhibitor, discovered in 2023. HP-4 exhibited the potent inhibitory effects against HSP90 with IC_50_ of 18 nM and effectively inhibited the growth of tumor. The discovery of these novel derivatives demonstrated that resorcinol-based inhibitors have large potential for the further investigation into the development of anti-tumor drugs.^437^ Recently, a series of HSP90 inhibitor containing the aryl-resorcinol scaffold were designed and investigated the SARs, elucidating the dissimilarity in ligand selectivity between the isoforms of HSP90.^438^ Compound 30 f, a novel benzamide derivative, was discovered in 2018 and showed high HSP90 binding affinity (IC_50_ = 5.3 nM). Importantly, compound 30 f exerted anticancer activity against non-small cell lung cancer.^439^ In additional of the four classic HSP90 ATPase inhibitors mentioned above, other structures with HSP90 inhibitory activity have been identified recently. Capsaicin, a natural product, was discovered to bind the N-terminus of HSP90, enhancing the anti-tumor effects of 17-AAG.^440^ Besides, novel tropane analogs derived from XL888 displayed significant HSP90 inhibitory activity with IC_50_ of 57 nM.^441^ Collectively, these findings support that novel chemical scaffolds might be used to design new HSP90 N-terminus inhibitors.

**HSP90 CTD inhibitors**

The CTD plays important roles in modulating the structure and function of HSP90 (Fig. 7a). In addition to a calmodulin-binding site and an HSP90 homodimerization motif, the HSP90 CTD also contains an ATP-binding site that allosterically modulates its ATPase activity.^442^ Many natural products and their derivatives have been reported as the potent HSP90 CTD inhibitors (CTIs). HSP90 CTIs can be classified into three categories, including novobiocin-based, deguelin-based and peptide-based CTIs (Table 3). Novobiocin was the first CTI with HSP90 inhibition and antiproliferative action on MCF-7 cells with IC_50_ of 256 μM. After novobiocin treatment, the level of clients of HSP90 decreased.^443,444^ Studies suggested that novobiocin binds to sequence of Glu537 to Asn686 of HSP90 CTD in open conformation, allosterically modulating HSP90 and preventing the ATP binding to HSP90 NTD.^445,446^ Due to the unsatisfactory antitumor activity of novobiocin, many novobiocin-based analogs have been prepared and some comprehensive SAR studies were elucidated, which led to improved antiproliferative activity of the CTIs. Of these, the best analog showed excellent inhibitory activity against SKBr3 cells with IC_50_ of 170 nM.^447^ Recently, a class of noviose sugar surrogates, as noviomimetics, were discovered as novel HSP90 CTIs, which performs potent anti-proliferative activities on SKBr3 cells with IC_50_ of 560 nM.^448^ Recently, new class of HSP90 CTIs were discovery by ligand-based pharmacophore screening or fragment hybrids, including TVS21 derivative and compound 2n (Table 3). They showed anti-tumor effect against triple-negative breast cancer (TNBC), highlighting the promise of HSP90 CTIs for TNBC therapy.^449–451^ Deguelin, a naturally occurring rotenoid, is another important class of CTIs. Deguelin seems to form crucial interactions with Ser677 and Lys615 of HSP90 CTD, disrupting HSP90 function of binding nucleotide.^452^ A study in vivo showed that deguelin significantly reduced cancer growth by decreasing the expression of HSP90 clients, with no observable toxicity.^453^ The latest discovered derivative, HVH-2930, fits into the CTD ATP-binding pocket interface cavity of the HSP90 homodimer, which stabilizes the open conformation of HSP90 and hinders ATP binding. Particularly, HVH-2930 exhibits potent efficacy against trastuzumab-resistant HER2^+^ breast cancer and reasonable pharmacokinetic and toxicity profiles, which would promote the clinical application of HSP90 CTIs.^454,455^ Among peptide-based CTIs, penisuloxazin A binds to oxidize cysteine residues (Cys572, Cys597 and Cys598) at a site of HSP90 CTD, distinct from ATP binding pocket. Penisuloxazin A showed antiproliferative activity against various cancer cells, indicating that the type inhibitor is worthy of being studied in the future.^456,457^ Compared with HSP90 NTD inhibitors, targeting HSP90 CTD does not induce the HSR. However, the structures of HSP90 CTD have been not obtained. The molecular mechanism of the binding mode and the efficacy of HSP90 CTIs against cancer cells remain unclear.^458^

**HSP90 MD inhibitors**

The HSP90 MD is the binding part for clients and many co-chaperones, and also involved in regulating the conformational changes of HSP90, playing a crucial role in its function (Fig. 7a). Targeting HSP90 MD is also a potential strategy to achieve the inhibition of HSP90 (Table 3). Recently, natural product enniatin A and diptoindonesin G were demonstrated to bind with HSP90 MD, which destabilized its client oncoproteins and promoted their degradation. Compared to HSP90 ATPase inhibitor, the MD inhibitors specifically affect the binding between HSP90 and substrates or co-chaperones, instead of inhibiting ATP hydrolysis and further completely blocking the function of HSP90. Therefore, the HSP90 MD inhibitors can prevent HSR, providing insights for developing HSP90 inhibitors without serious side effects.^459–461^ However, there is limited studies on HSP90 MD inhibitors, and further studies are necessary for the elucidation of the detailed binding mechanism.

##### Stage 2: targeting HSP90 isoforms with high selectivity (from 2000s)

Although four human HSP90 isoforms are highly conserved, they perform distinct functionalities in different cellular structures. HSP90α is the stress-inducible isoform, while HSP90β is constitutive. HSP90α/β are mainly expressed in the cytoplasm and are closely related to cancer-related signaling pathways.^462,463^ GRP94, an ER isoform, promotes cell migration and influences intracellular trafficking. And TRAP-1, a mitochondrial isoform, has anti-apoptotic and antioxidant properties, protecting mitochondrial integrity.^464,465^ With the gradual reveal of crystal structures for different isoforms, the development of HSP90 inhibitors entered into stage 2: targeting isoforms with high selectivity based on unique binding sites on these isoforms (Fig. 7b).

**TRAP-1 selective inhibitors**

TRAP-1 is mitochondria-specific HSP90 isoform, regulating mitochondrial integrity. TRAP-1 was found to be increased in tumor cells, including breast, colon and lung cancers.^466,467^ Compared to other three isoforms, the limited information available on crystal structure of TRAP-1 has posed challenges for discovering TRAP-1 selective inhibitors (Table 3). In 2015, Jeong et al. achieved a significant milestone by successfully determining the structure of TRAP-1 bound with PU-H71 that is a purine-based HSP90 inhibitor.^468,469^ Based on the TRAP-1-PU-H71 cocrystal structure, the sequence from Leu172 to Phe201 exhibits disorder in the binding site of TRAP-1 NTD, and two conserved residues, Asn171 and Gly202, adopt distinct conformations, which could provide chances for design novel TRAP-1-selective inhibitors. Based on that, DN401 was discovered by modifying the imidazole ring of PU-H71. This alteration facilitated extra π-π interactions with Phe201 and hydrogen bonds with Asn171 in TRAP-1, contributing to accumulation of DN401 in mitochondria and a 9-folds selectivity for TRAP-1 (TRAP-1: K_d_ = 79 nM; HSP90α/β: K_d_ = 698 nM).^470^ Due to the distinctive feature of mitochondrial localization, it is possible for TRAP-1 to discover selective inhibitors using mitochondrial delivery vehicle. SMTIN-P01 was design by introducing a mitochondria-targeting triphenylphosphine (TPP) moiety into PU-H71, which achieved accumulation of the molecule in mitochondria and selectively impaired the chaperone activity of TRAP-1.^471^ Besides, there are two TRAP-1 selective inhibitors containing TPP moiety, including gamitrinib-TPP and 6BrCaQ-TPP.^472^ Gamitrinib-TPP is another conjugate of 17-AAG and TPP moiety and entered into clinical trial of advanced cancers, which proved the feasibility of design TRAP-1 selective inhibitors using mitochondria-targeting moieties.^473,474^

**GRP94 selective inhibitors**

GRP94 is a master regulator of ER homeostasis, assisting stabilization of many clients that are responsible for cancer and inflammatory diseases.^475^ Therefore, elective inhibiting of GRP94 may be a point for the treatment of many diseases. Currently, GRP94 inhibitors are the most studied HSP90 isoform-selective inhibitors, which includes three classes: purine-based, benzamide-based and resorcinol-based (Table 3). NECA, containing the purine moiety, is the firstly reported GRP94 inhibitor in 2000s, later identified high selectivity for GRP94 inhibition over 89-folds compared to HSP90α (GRP94: K_d_ = 0.53 μM; HSP90α: K_d_ = 46 μM).^476,477^ Crystallographic studies revealed that the purine moiety interacts with adenine binding pocket of GRP94, and the ethyl group can form hydrophobic interactions with Val197 and Tyr200, leading to the achievement of high selectivity for GRP94.^34^ To further develop purine-based GRP94 inhibitors, SAR studies were performed and obtained many selective GRP94 inhibitors that opened the doors to design of this class inhibitors. Among them, PU-H39 was discovered in 2015, exhibiting the best selectivity for GRP94 inhibition with over 200-folds for GRP94 vs HSP90α/β (GRP94: IC_50_ = 2.4 μM; HSP90α/β: IC_50_ = > 500 μM). Compared with NECA, the alkyne of PU-H39 mediates π–π interactions with Phe195 in GRP94, greatly improving the selectivity for GRP94.^478,479^ At the same time, BnIm, a resorcinol-based analogous, was also reported to selectively inhibit GRP94 with 12-folds selective (GRP94: IC_50_ = 1.2 μM; HSP90α: IC_50_ = 1.4 μM).^478^ The phenyl ring in BnIm could form hydrophobic interactions with Met154, Leu163, Val211 and Trp223 of GRP94.^480^ To obtain higher selective GRP94 inhibitors based this structure, SAR studies on phenyl groups were developed and revealed that polar and large substituents were not necessary to improving selectivity. Introduction of a fluorine atom into the phenyl lead to the discovery of KUNG65 that raised to 73-folds selectivity for GRP94 inhibitor, though with weak binding with GRP94 (GRP94: K_d_ = 0.54 μM; HSP90α: K_d_ = 39 μM).^481^ Based on the structure of KUNG65, Brian S.J. Blagg et al. further studied SAR of benzamide substituents and found that larger saturated ring system manifested increased selectivity for GRP94 over HSP90α, providing inspiration for structural modifications.^482^ The representative inhibitor of the third class benzamide-based is ACO1, modified from a non-selective inhibitor, SNX-2112. Compared with resorcinol-based analogous, ACO1 showed over 200-folds selectivity (GRP94: K_d_ = 0.44 μM; HSP90α: K_d_ = > 100 μM). The pyrrole of ACO1 seems to form π-π interactions with Phe195, leading to improvement of selectivity for GRP94.^483^ Our group further optimized the type inhibitors though ligand-induced Phe199 shift mechanism, and promoted the obtainment of DDO-5813 that showed a selectivity of more than 1000-fold for GRP94 over HSP90α (GRP94: IC_50_ = 2.0 nM; HSP90α: IC_50_ = > 100 μM).^484,485^ In summary, the class of isoform selective inhibitors relies on classic hydrophobic cavities of GRP94 NTD that distinguish from the other three isoform of HSP90. These GRP94 selective inhibitors have been demonstrated to show potential therapeutic effects on various cancer models and inflammatory models in preclinical studies.^478,485^

**HSP90α/β selective inhibitors**

HSP90α/β, locating in the cytoplasm, regulates many signaling pathways closely related to cancers. It is vital to design inhibitors to selectively inhibit HSP90α/β for reduction of side effects. In addition to NVP-AUY922 and NVP-BEP800 that entered clinical trials,^403,486,487^ other HSP90α/β selective inhibitors in preclinical trials were reported (Table 3). EC144, a novel purine-based compound, was discovered in 2014. The structure of EC144 complexed with HSP90 NTD showed a hydrophobic interaction between hydroxyallyl substituent and the adjacent side chains of Met98 and Leu107, which resulted in > 300-folds selectivity for cytosolic HSP90 isoforms (HSP90: K_i_ = 0.20 nM; TRAP-1 K_i_ = 255 nM; GRP94: K_i_ = 61 nM). In addition, a novel benzolactam series of HSP90α/β inhibitors were obtained from structure-based drug design strategy. The most potent inhibitor among them is compound 31, which exhibits over 1000-folds selectivity against HSP90α/β and two other isoforms (HSP90α/β: K_i_ = 5.0 nM; GRP94: K_i_ = >10 μM; TRAP-1: K_i_ = >10 μM). And compound 31 showed orally availability and could cross the blood-brain barrier, which suggested its possibility of treating chronic neurodegenerative indications.^488^

**HSP90β selective inhibitors**

In addition to highly correlated with cancers, HSP90β was also proved to modulate muscle regeneration and μ-opioid receptor function. Due to the functional differences between HSP90α and HSP90β, achieving selective inhibition between them is crucial for realizing precise regulation.^489,490^ However, achieving selectivity for either HSP90β or HSP90α is challenging, as the high identity (95%) of two isoforms. Therefore, the discovery of KUNB31 in 2018, a resorcinol derivative, is the biggest breakthrough in the development of HSP90 inhibitors. In order to achieve selectivity, Khandelwal et al. analyzed the binding mechanism of radicicol. They predicted that the bulkiness of HSP90α, GRP94, and TRAP-1 in comparison to HSP90β could serve as a basis for designing inhibitors. Subsequent research revealed that incorporating hydrophilic substituents is not favorable for binding with HSP90α, ultimately resulting in the identification of KUNB31 (Table 3). Besides, KUNB31 specifically interacts with Asp88 and Thr179 of HSP90β and promotes the formation of hydrogen bonds, which results in the high affinity for HSP90β (K_d_ = 0.18 μM), approximately 50-fold more selective than other isoforms (GRP94: K_d_ = 8.5 μM; HSP90α: K_d_ = 9.6 μM).^114,491^ Specially, a natural product gambogic acid, containing a pharmacophore, was identified to bind uniquely to a site in HSP90β (Table 3). Various binding studies demonstrated that gambogic acid specifically recognizes the HSP90β MD, resulting in over 3-folds selectivity compared HSP90α.^492^ Besides, corylin was found to selectively inhibit HSP90β in 2019. Though binding to HSP90β MD, corylin promoted ubiquitination and proteasomal degradation of sterol regulatory element-binding proteins, which significantly reduced lipid content in both liver cell lines and human primary hepatocytes.^493^ More characterization of the novel isoform-selective ligands for HSP90β MD of were reported subsequently, which provides new sites for designing selective HSP90β inhibitors.^494^

**HSP90α selective inhibitors**

Recently, several high selective HSP90α inhibitors were also discovered based on AT13387 (Table 2). Among them, KUNA-115 showed the most the highest selectivity, over 60-folds for HSP90α compared with HSP90β (HSP90α: IC_50_ = 0.84 μM; HSP90β: IC_50_ = > 50 μM). SAR studies were subsequently reported and demonstrated that a 5-fluoroisoindoline derivative (KUNA-111) revealed a novel binding mode, though KUNA-111 only maintained 15-folds selective for HSP90α. The binding pattern of these derivatives with HSP90α revealed significant hydrogen bonding with Leu48, Asn51, Asp93, and Thr184, which are crucial for ensuring specificity in HSP90α.^495,496^ Interestingly, based on the high selective HSP90α inhibitor KUNA-115, the team also reported that the introduction of cell-permeable dimethylamine into the solvent-exposed portion of KUNA-115 significantly improved selectivity of the molecule for HSP90α, over 196-folds compared with HSP90β. Besides, the replacement of permanently charged moieties further enhanced its selectivity between the two isoforms, over 294-folds.^497^ These findings offer valuable insights for the advancement of HSP90α selective inhibitors.

**eHSP90 selective inhibitors**

As the regulatory functions of eHSPs are gradually revealed, targeting eHSP90 also becomes a potentially inhibitory strategy in cancer progression. A permanently positively charged quaternary ammonium was introduced into KUNA-115 that is a eHSP90α-selective NTD inhibitor, leading to discovery of the first cell-impermeable eHSP90α-selective inhibitor NDNA4 (Table 3). NDNA4 showed an affinity of 0.34 μM for eHSP90α. NDNA4 inhibited eHsp90α’s stabilization of cell-surface receptors, without inducing degradation of the receptors.^497^ Similarly, DMAG-N-oxide was obtained by introducing a permanent N-oxide N^+^O^−^ zwitterion into GDA (Table 3). As the transmembrane is blocked, DMAG-N-oxide acts on eHSP90 in cell surface and inhibits cell metastasis of T24 cells in vitro.^498^ Biotin is also a cell-impermeable fragment, which was linked to Ganetespib to obtain STA-12-7191 (Table 3). Compared with Ganetespib, STA-12-7191 showed better inhibition of cell migration at 10 nM against MDA-MB231 breast cancer cells.^499^ Additionally, several other inhibition strategies targeting eHSP90 have been also identified. The plasma membrane-associated heparan sulfate proteoglycans was identified to bind the eHSP90, further promoting the tumor development mediated by eHSP90. Subsequently, a conjugate of 2,5-dihydroxybenzoic acid with gelatin (2,5-DHBA-gelatin) was designed to inhibit eHSP90, which further suppressed the migration of A-172 and HT1080 cells.^500^ In addition to small molecules by applying cell-impermeable strategy, antibodies targeting eHSP90 were also found to inhibit eHSP90 functions. In 2004, monoclonal antibody 4C5 was found to inhibit cell migration from cerebellar and sciatic nerve, explants.^501^ Subsequently, 4C5 was identified as an anti-HSP90 cell-impermeable monoclonal antibody, binding to surface HSP90 and not affecting the function of intracellular HSP90. 4C5 inhibits cancer cell invasion in vitro and the metastatic deposit formation of MDA-MB-453 cells into the lungs of severe combined immunodeficiency disease mice in vivo.^502^ In addition to 4C5, 11C9 and 1G6-D7 were also found to inhibit eHSP90. 11C9 inhibited invasion and self-renewal abilities of hepatocellular carcinoma cell lines, while 1G6-D7 affected tumor immunity in non-small cell lung cancer.^503,504^ Recently, HH01, with novel complementarity-determining regions, was reported to exhibit high binding affinity toward eHSP90α. In mouse models, HH01 potently inhibited the tumor growth of pancreatic ductal adenocarcinoma cell xenografts.^505^ The development of antibody therapeutics to inhibit eHSP90 provides a potential method against cancer.^506^ Mechanically, the inhibition of eHSP90 can avoid inducing HSR and intracellular HSP90-dependent client degradation. The results demonstrate that targeting eHSP90 has less toxicity and side effects. Therefore, as an alternative strategy, development of eHSP90 selective inhibitors suggests the potential of cancer treatment.

##### Stage 3: targeting PPIs between HSP90 and co-chaperones (from 2010s)

HSP90 possesses various types of co-chaperones that fulfill distinct functions, including regulating its ATPase activity, affecting binding and release of substrates and assisting in the completion of HSP90 molecular chaperone cycle. Due to different functions and binding characteristics of co-chaperones, inhibiting their PPIs with HSP90 can specifically hinder the formation of functional molecular chaperone system of HSP90, further effectively impeding the maturation and release of pathogenic proteins (Fig. 7c). Therefore, targeting PPIs between HSP90 and different co-chaperones was considered as potential strategies, representing stage 3 development of HSP90 inhibitors (Table 3).

**Inhibitors targeting HSP90-CDC37 PPIs**

CDC37 can specifically identify approximately 90% of the unfolded kinase and recruit them into HSP90 cycle.^47^ The inhibition of HSP90-CDC37 PPIs was believed as a potential strategy to prevent the maturation and release of disease-activated kinases (Fig. 7c). Currently reported HSP90-CDC37 PPIs inhibitors can be classified into three types: natural products, peptides and small molecules (Table 3). Natural products are the most commonly reported inhibitors of the HSP90-CDC37 PPIs. Celastrol A is the first reported natural product with HSP90-CDC37 PPIs inhibitory activity. In 2008, Zhang et al. firstly reported a binding pocket on HSP90 that blocked some interactions between HSP90 and CDC37 through molecular dynamics simulations. Subsequently, they found that the natural product bound to HSP90 CTD, leading to disruption of HSP90-CDC37 interactions.^507,508^ The treatment of celastrol A resulted in the downregulation of kinase client AKT and CDK, and further exhibited anti-proliferative activity against pancreatic cancer cells (IC_50_ = 3.0 μM). Therefore, celastrol A was known as a starting point for discovery HSP90-CDC37 PPI inhibitors. Subsequent SAR studies of celastrol derivatives as HSP90-CDC37 disruptors were elucidated. The anti-tumor activity of this class of inhibitors has been raised to the nanomolar level, as well as exhibiting druglike properties.^509–511^ Besides, many natural products have been discovered to possess inhibitory activities for HSP90-CDC37 PPIs, including previously reported kongensin A,^512^ platycodin D,^513^ sulforaphane^514^ and FW-04-806,^515^ as well as recently reported ginsenoside Rg5,^516^ okicamelliaside^517^ and elaiophylin.^518^ Importantly, these natural products have demonstrated potent anti-cancer activities, suggesting that targeting the HSP90-CDC37 PPIs is a promising strategy for drug discovery, and natural products are an important source for develop PPIs inhibitors. Due to that endogenous peptides can locate and uncover the “hot‐spots” at the binding interface of PPIs, peptide mimics are also important for discovering PPIs modulators. Our group reported the first peptide (Pep-1) with disruption effect of HSP90-CDC37 PPIs, which contains eleven-residues and derives from CDC37 sequence. Pep-1 directly bound to the HSP90 NTD with a moderate binding affinity (K_d_ = 7.13 μM).^519^ Recently, we further identified that a shorter peptide derived from CDC37 sequence, named as DDO-59120, maintained the similar binding affinity for HSP90 NTD (K_d_ = 6.9 μM). DDO-59120 also potently disrupted the HSP90-CDC37 PPIs.^520^ In 2017, our group reported the first non-natural derived HSP90-CDC37 PPIs inhibitor with moderate binding capacity. Although the mechanism binding to targets was unclear, the molecule paved the way for discovery of non-natural small molecule inhibitors.^521^ Subsequently, based on the molecular dynamic simulations and mutagenesis studies, we discovered the key binding interactions between Glu47 and Gln133 on HSP90 and Arg167 on CDC37. Then, DDO-5936 was identified by high-throughput screening and further SAR studies, which exhibited disruption activity of HSP90-CDC37 PPIs via binding to HSP90 (K_d_ = 7.4 μM). DDO-5936 was also found to exhibit antiproliferative activity against numerous cancer cell lines by G0/G1 cell cycle arrest. DDO-5936 led to the degradation of kinases in HCT116 cells without inducing HSR,^522^ which brought a new breakthrough for HSP90-CDC37 PPIs inhibitors. Besides, DCZ3112 was also reported to inhibit the HSP90-CDC37 PPIs, leading to the down-regulation of HSP90 client proteins. In addition to antiproliferative effect on HER2^+^ breast cancer cells, DCZ3112 resolved its resistance to traditional HSP90 inhibitors, providing a therapeutic potential to develop anti-breast cancer drugs.^523^

**Inhibitors targeting HSP90-Aha1 PPIs**

As a co-chaperone, the NTD of Aha1 can be recruited to HSP90 MD, accelerating the closed conformation of HSP90 and enhancing HSP90’s inherently low ATPase activity (Fig. 7c). Therefore, the inhibition of HSP90-Aha1 effectively suppressed hydrolysis of ATP, further blocking HSP90 cycle. In 2017, two types of HSP90-Aha1 inhibitors were respectively reported, including derivatives of natural products and small molecules obtained from high-throughput screening.^524,525^ Among them, the biological mechanism and inhibitory effect of small molecule HAM-1 was clearly studied (Table 3). HAM-1 binds to the HSP90 NTD with an affinity of 24 μM. Further studies found that HAM-1 inhibited the catalytic function of Aha1 as an accelerator of the ATPase, while Aha1 is still bound to HSP90. The inhibition of HAM-1 selectively effected HSP90-Aha1 PPIs by suppressing hydrolysis of ATP, which preliminarily demonstrated the feasibility of targeting HSP90-Aha1 PPIs.^526^

**HSP90-p23 PPIs inhibitors**

The HSP90-p23 PPIs stabilize the interactions of HSP90 with the client substrates by inhibiting HSP90’s ATPase activity, which promotes the folding and maturation of the substrates (Fig. 7c). p23 was reported to be related to metastasis and advanced malignancy, making the inhibition of HSP90-p23 PPIs an appealing strategy (Table 1). CP9, a small molecule discovered by high throughput screening (HTS) in 2012, was firstly found to disrupt HSP90-p23 PPIs. CP9 competitively binds to the HSP90 NTD ATPase site, thereby interfering with its interaction with p23. The disruption led to the instability of substrates binding with HSP90, promoting degradation of AKT and Raf-1 in multiple cancer cell lines, and further displaying antiproliferative activity. Subsequently, SAR studies were performed to obtain more potent lead compounds, providing more inspirations for discovery of HSP90-p23 PPIs inhibitors.^527^ Natural products are another commonly reported class of HSP90-P23 PPIs inhibitors, such as cucurbitacin D that is isolated from the fruit of *Cucurbitaceae*. Cucurbitacin D was found to concurrently inhibit HSP90-CDC37/P23 PPIs. The inhibition resulted in the degradation of HSP90 clients in a dose-dependent manner without inducing HSR, which thereby showed the antiproliferative effect on MCF-7 breast cancer cells (IC_50_ = 0.60 μM).^528^ Apart from cucurbitacin D, other natural products were also reported to inhibit HSP90-P23 PPIs, including docosahexaenoic acid. However, the mechanism of action of these natural products is still unclear.^529^

**PPIs inhibitors of HSP90-co-chaperones containing TPR domain**

Many co-chaperones containing TPR domain exert significant regulatory effect on HSP90, such as PP5, HOP, Cyp40. Among them, HOP plays a crucial role in assisting the transfer of unfolded client proteins from HSP70 to HSP90 for further maturation (Fig. 7c). The disruption of HSP90-HOP PPIs can block the transformation of substrates to HSP90, thereby preventing the maturation and release of pathogenic substrate proteins. In 2008, some compounds containing heterocyclic core were identified to disrupt HSP90-HOP PPIs and downregulate the expression of clients of HSP90, which provides a novel strategy for inhibiting HSP90 cycle.^530^ Y‐632 is one of extensively studied HSP90-HOP inhibitors. Further mechanism studies identified that Y‐632 directly interacted with HSP90 and specifically disturbed HSP90-HOP PPIs, rather than inhibiting HSP90 ATPase activity (Table 3). Importantly, Y‐632 hinders imatinib‐resistant cell growth.^531^ Therefore, it is believed that disruption of HSP90-HOP PPIs is a promising strategy. Besides, immunophilins, such as FKBPs, are a class of co-chaperones containing-TPR domain, regulating the progression of the HSP90 protein folding cycle.^532,533^ In 2014, McAlpine et al. obtained the first inhibitor, SM145, could inhibit the interaction between HSP90 and multiple immunophilins via disrupting the binding between MEEVD and TPR domain (Table 3). SM145 binds at a new pocket at between the NTD and MD of HSP90, moderately reduced the levels of GR in HeLa cells without induction of HSR.^534^ Due to that this class of co-chaperones play complex roles in the HSP90 cycle, selectively inhibiting PPIs of HSP90 with them would achieve precise regulation of substrates.^535^

##### Stage 4: design of multi-specific molecules based on HSP90 (from 2020s)

Small molecule targeted protein degradation (TPD) has garnered significant attention in recent years. In addition to its role in protein folding and maturation, HSP90 can also promote the degradation of misfolded proteins via ubiquitin-proteasome system (UPS). Therefore, design of multi-specific molecules that recruit HSP90 to selective degradation of pathogenic proteins become an appealing strategy, which makes the development of HSP90 inhibitors entered into stage 4 (Fig. 7d). The first multi-specific molecule RNK05028 was reported in 2021 (Table 3) and termed as chaperone-mediated protein degraders (CHAMPs). RNK05028 consist of BET inhibitor JQ1, linker and HSP90 inhibitor STA-9090. RNK05028 chemically induced proximity between transcription factor BRD4 and HSP90, selectively promoting the degradation of BRD4. Notably, RNK05028 displays tumor-targeted pharmacokinetics and prolonged specifical degradation of BRD4 in vivo, which demonstrated the potential of the novel TPD strategy.^536^ In 2023, another multi-specific molecule recruiting HSP90, HEMTAC 26, was also designed for selective degradation of CDK4/6 with DC_50_ of 26/19 nM (Table 3). HEMTAC 26 is composed of HSP90 inhibitor BIIB021 and CDK4/6 inhibitor palbociclib. HEMTAC 26 dose-dependently decreased the levels of CDK4/6 and inhibited tumor growth in the B16F10 Xenograft Model.^537^ Undoubtedly, the discovery of multi-specific molecules based on HSP90 expands the scope of TPD strategies and may avoid drug resistance mediated by mutations of specific ubiquitin E3 ligases.

In addition to recruiting HSP90 as an effector to achieve degradation of target proteins, the multi-specific molecules that achieve HSP90 degradation by E3 ubiquitin ligand has also been design and investigated. In 2022, a series of PROTACs targeting HSP90 were designed for the first time, of which BP3 potently degraded HSP90 and effectively inhibited the growth of MCF-7 cells (IC_50_ = 0.6 μM). Importantly, BP3 led to effective tumor suppression in mice. To reduce toxic side effects, the same group designed a novel PROTAC X10g using pomalidomide and AT13387 derivative, which electively degraded HSP90α without affecting HSP90β (DC_50_ = 0.3 μM). X10g showed anti-tumor activity in vivo and had no significant effect on the body weights of mice.^538,539^ Besides, lw13, a novel HSP90-targeting PROTAC, recently was reported, effectively degrading HSP90 at a concentration of only 0.05 μM. Furthermore, lw13 showed a synergistic effect in combination with cisplatin against cervical cancer.^540^ Overall, these results demonstrate the potential of the HSP90-directed PROTAC strategy for cancer therapies.

#### Targeting therapy of HSP70

##### Stage 1: targeting pan-HSP70 isoforms (from 1990s)

**Fig. 8:**
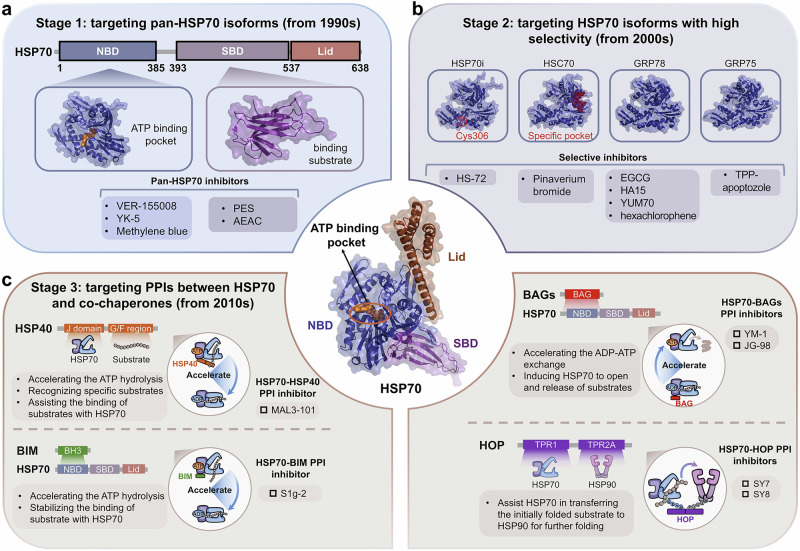
Targeting therapy of HSP70. **a** Stage 1: targeting pan-HSP70 isoforms (from 1990s), including HSP70 NBD ATPase inhibitors and SBD inhibitors. **b** Stage 2: targeting HSP70 isoforms with high selectivity (from 2000s). Selective binding sites of HSP70 isoforms have been respectively reported, leading to discovery of inhibitors with high selectivity, including HSP70i selective inhibitors, HSC selective inhibitors, GRP78 selective inhibitors and GRP75 selective inhibitors. **c** Stage 3: targeting PPIs between HSP70 and co-chaperones (from 2010s), including HSP70-HSP40 PPI inhibitors, HSP70-BAG PPI inhibitors, HSP70-BIM PPI inhibitor and HSP70-HOP PPI inhibitors

As one of most important molecule chaperons, HSP70 also is essential for protecting cells against stress and contributing to cell survival.^541^ In the development of HSP90 inhibitors, HSP70 was also concurrently investigated as a significant target associated with diseases (Fig. 8a). Due to the high identity between HSP70 family members, initial inhibitors were design to target pan-HSP70 isoforms (Stage 1, from 1990s). They can be classified into two types, including HSP70 NBD inhibitors that bind to ATPase site to inhibit ATP hydrolysis, and HSP70 SBD inhibitors that interact with substrate-recognition site to block binding of substrates (Table 4). In 2009, VER-155008, an adenosine-derivative, was identified as a HSP70 inhibitor binding to NBD ATPase site. VER-155008 can competitively bind to HSP70 compared with ATP (IC_50_ = 0.50 μM). VER-155008 showed antiproliferative effects and induced HSP90 client protein degradation in HCT116 cells. Although the pharmacokinetics in vivo of VER-155008 was limited, inhibiting HSP70 still offered an exciting anti-cancer therapeutic strategy.^542,543^ Apart from VER-155008, the dye methylene blue also found to inhibit HSP70 ATPase by HTS. Methylene blue showed manifolds biological activities, inhibiting tau protein in neurodegenerative cell models.^544^ In 2014, a novel 2,5’-thiodipyrimidine-derivative YK-5 was designed based on the computational analysis of ligand-HSP70 interactions. YK-5 was proved to bind to a novel allosteric pocket of the HSP70 NBD and covalently interacted with a cysteine residue of the domain. YK-5 degraded Her2 and Raf-1 in SkBr3 by inhibiting HSP70, further showed a high apoptosis active against SkBr3 cells with IC_50_ of 0.80 μM.^545^ However, HSP70 NBD inhibitors could completely block ATP binding and inhibit normal function of HSP70, which may lead to potential toxicity. A novel compound, 2-phenylethynesulfonamide (PES), was also discovered to inhibit HSP70 by screening a library of drug-like small molecules in 2009.^546^ PES interacts with the carboxyl-terminal portion in the HSP70 SBD. Studies showed that PES exhibited antiproliferative activity against acute leukemia, as well as decreased the interaction of HSP70 with some co-chaperones. As an important inhibitor of HSP70, it has been extensively used in preclinical studies.^547,548^ Another representative HSP70 SBD inhibitor, N-aminoethylaminocolchicine (AEAC) was obtained from HTS and identified to interact with HSP70 SBD. The high affinity of AECE with HSP70 (K_d_ = 149 nM) achieved the inhibition of refolding activities of HSP70. These HSP70 SBD inhibitors occupied the substrate-binding site, and further blocked the recognition of HSP70 for substrates. Compared to HSP70 NBD inhibitors, HSP70 SBD inhibitors specifically disrupt the binding of substrates, which may be an effective strategy for avoiding toxicity.^397,549^ However, the inhibitory activities and the specificity of currently reported HSP70 inhibitors need to be further improved, which greatly limits the development of HSP70 inhibitors.

**Table 4:** Preclinical studies of targeting HSP70

Compound	Structure	Activity	Mechanism (Sites)
VER-155008		^b^IC_50_ = 0.50 μM	Pan-HSP70 inhibitor (NBD)
methylene blue		/	Pan-HSP70 inhibitor (NBD)
YK-5		^c^IC_50_ = 0.80 μM (SkBr3 cells)	Pan-HSP70 inhibitor (NBD)
PES		/	Pan-HSP70 inhibitor (SBD)
AEAC		K_d_ = 149 nM	Pan-HSP70 inhibitor (SBD)
Apoptozole		HSP70i: K_d_ = 0.14 μM; HSC70: K_d_ = 0.21 μM	HSP70i/HSC70 selective inhibitor (NBD)
HS-72		/	HSP70i selective inhibitor (NBD)
Pinaverium bromide		^c^IC_50_ = 10 μM (A2058 cells)	HSC70 selective inhibitor (NBD)
EGCG		K_d_ = 0.70 μM	GRP78 selective inhibitor (NBD)
HA15		^a^IC_50_ = 25–50 μM	GRP78 selective inhibitor (NBD)
YUM70		^a^IC_50_ = 1.5 μM	GRP78 selective inhibitor (SBD)
Hexachlorophene		^d^IC_50_ = 9.1 μM	GRP78 selective inhibitor (SBD)
TPP-apoptozole		/	GRP75 selective inhibitor (SBD)
MAL3-101		/	HSP70-HSP40 PPI inhibitor (NBD)
S1g-2		^e^IC_50_ = 0.40 μM; K_d_ = 0.84 μM	HSP70-BIM PPI inhibitor (NBD)
YM-1		^e^IC_50_ = 4.8 μM; K_d_ = 5.8 μM	HSP70-BAGs PPI inhibitor (NBD)
JG-98		^e^IC_50_ = 1.6 μM; K_d_ = 86 nM	HSP70-BAGs PPI inhibitor (NBD)
SY7		/	HSP70-HOP PPI inhibitor (SBD)
SY8		/	HSP70-HOP PPI inhibitor (SBD)

*K*_*d*_ target binding affinity; *K*_*i*_ target binding affinity; ^*a*^*IC*_*50*_ ATPase inhibitor activity; ^*b*^*IC*_*50*_ competitive binding activity; ^*c*^*IC*_*50*_ anti-proliferative activity; ^*d*^*IC*_*50*_ substrate competitive inhibitory activity; ^*e*^*IC*_*50*_ PPIs inhibitory activity; “/” indicates no clear reports

##### Stage 2: targeting HSP70 isoforms with high selectivity (from 2000s)

The four main isoforms of HSP70 including inducible HSP70 (HSP70i), constitutive HSC70, GRP78 and GRP75. Like the distribution of HSP90, HSP70i and HSC70 are mainly located at cellular cytoplasm, while GRP78 and GRP75 are located in the ER and mitochondria, respectively.^550,551^ HSP70 isoforms were reported to respectively overexpress in a wide spectrum of human tumors.^552^ Therefore, selective inhibitors of HSP70 are vital for treatment of different diseases (Fig. 8b). However, HSP70 family members are highly related with 50-80% identity and HSP70i and HSC70 shared 90% sequence identity, contributing to vast difficulties for design selective inhibitors.^553^ Nevertheless, some selective inhibitors targeting different isoforms from HSP70 family members have been successfully developed, through analysis of the specific pockets of and their differences in binding sites for various substrates.^554^

**HSP70i/HSC70 selective inhibitors**

HSP70 family has multiple functions in suppressing apoptotic pathways, particularly HSP70i and HSC70.^553^ Apoptozole, was designed as a pro-apoptotic inhibitor of HSP70i (K_d_ = 0.14 μM) and HSC70 (K_d_ = 0.21 μM). Apoptozole was assessed to induce the death of some cancer cells, such as SK-OV-3, HCT-15, and A549.^555^ Subsequently, the compound was identified to bind to NBD of HSP70i and HSC70 and inhibit ATPase activity, without binding to other HSPs.^556^ The studies in vivo showed that apoptozole led to 61% volume reductions of the A549 xenografts, compared with the vehicle-treated group.^557^ While apoptozole has demonstrated antitumor efficacy in animal models, its potential toxicity and long-term safety in normal tissues remain incompletely elucidated.

HS-72 is the first selective inhibitor for HSP70i. Mechanism research indicated HS-72 interacted with C306 that is a possible allosteric site within the NBD of HSP70i. The allosteric interaction of HS-72 induced conformational change of HSP70i, leading to reduction of affinity between HSP70i and ATP, without directly inhibiting ATP hydrolysis. C306 is not conserved amongst other members, which achieved the high selectivity of HS-72 for HSP70i. Importantly, HS-72 not only showed potent anti-proliferative activity against the tumorigenic breast lines, but also reduced tumor growth in vivo.^558^ Besides, subsequently studies exhibited that HS-72 could inhibit dengue virus infection, highlighting the potential of HSP70i as an antiviral target.^559^ Due to constitutive expression of HSC70 within the cell, depletion of HSC70 significantly reduces proliferation, migration and invasion, and promotes apoptosis of cancer cells.^560^ Inhibiting HSC70 was also believed as an effective strategy of anti-cancers. 15-deoxyspergualin and its derivatives were initially identified as HSC70 inhibitors. They inhibited the endogenous and co-chaperones stimulated ATPase activity of HSC70 respectively, and blocked the HSC70-mediated translocation, while their action of mechanism is unclear.^561,562^ In 2021, pinaverium bromide, a locally acting spasmolytic agent of the digestive tract, was also identified as an inhibitor of HSC70 through drug repurposing strategy.^563^ Docking and molecular dynamics predicted that pinaverium bromide could bind with the NBD and linker domains of HSC70 by specifical hydrophobic interactions. Importantly, pinaverium bromide inhibited cell proliferation of A2058 melanoma cells and induced apoptosis (IC_50_ = 10 μM), identifying HSP70 as a new therapeutic target for melanoma.^563^ However, HSP70i/HSC70 selective inhibitors are still in the positive early stage. It is necessary to further improve their activity and clarify their biological mechanism.

**GRP78 selective inhibitors**

GRP78, primarily localized in the ER, is translocated to the cell surface in stressed cell, which is involved in the maintenance and progression of many diseases. And the overexpression of GRP78 is found in many cancers and is implicated in tumorigenesis, invasion and drug resistance.^564^ Currently, various small molecular inhibitors targeting GRP78 have been discovered, including natural products and synthetic small molecule. In 2006, catechin (−)-epigallocatechin-3-gallate (EGCG), isolated from green tea, was reported to bind to the ATPase domain of GRP78 (K_d_ = 0.70 μM) and inhibit GRP78 ATPase activity. Importantly, EGCG has been shown to increase the sensitivity to a number of anti-cancer drugs. Therefore, it was suggested that EGCG could be used as an adjuvant drug, eliminating chemotherapy resistance to traditional anticancer drugs.^565–567^ Later, various natural products have been reported to inhibit GRP78 activity, including betulinic acid, honokiol and cucurbitacin B that directly bind to GRP78 with different affinity.^568–570^ HA15 is a representative synthetic small molecule inhibitor selectively targeting GRP78, derived from thiazolidinedione derivatives in 2016. The antitumor activity of HA15 was 1–2.5 μM in A375 cells and exhibited efficacious cytotoxicity to drug-resistant cell lines. Especially, HA15 significantly inhibits BRAF inhibitor-resistant melanoma in vivo. HA15 can directly bind to the ATPase domain of GRP78 and inhibit its ATPase activity, while its inhibitory activity is weak (IC_50_ = 25–50 μM).^571^ Therefore, GRP78 inhibitors with novel skeleton structure need to be developed. Among them, YUM70, a hydroxyquinoline derivative, was found by screening over 40,000 drug-like compounds. Subsequent studies showed that YUM70 inhibited GRP78 ATPase activity (IC_50_ = 1.5 μM) through binding to C-terminal SBD of GRP78 and the quinoline ring of YUM70 forms hydrogen bonds and stable hydrophobic interactions with some key residues. Xenograft model studies in vivo showed that YUM70 inhibited tumor growth and caused weight loss in treated mice.^572^ Hexachlorophene is another GRP78 inhibitor that could bind to the GRP78 SBD and obtained by screening compounds library. Hexachlorophene was identified as substrate competitive inhibitor (IC_50_ = 9.1 μM) and exhibited anticancer activity against HCT116 cells. However, antitumor efficacy of hexachlorophene is not sufficient and further SAR studies is necessary.^573^

**GRP75 selective inhibitors**

GRP75 is the mitochondrial isoform of HSP70, with an N-terminal mitochondrial localization motif. The level of GRP75 was observed to elevate in multiple cancerous tissues and tumor-derived cell lines, emphasizing its key role in oncogenesis.^574^ As described earlier, TPP moiety is a mitochondria-targeting device. Therefore, reported HSP70i/HSC70 inhibitor apoptozole was conjugated with TPP to obtain TPP-apoptozole that preferred to located in mitochondria and selectively targeted GRP75. Compared with apoptozole, TPP-apoptozole exhibited higher antiproliferative activity against different cancer cells. And mechanistic studies revealed that TPP-apoptozole inhibits the GRP75-p53 interaction and induces mitochondrial outer membrane permeabilization, consequently leading to mitochondria-mediated apoptosis.^575^ Recently, a chlorpromazine derivative was also reported to exhibit inhibitory activity against endometrial Cancer through directly targeting GRP75.^576^ Although the initial findings highlight the potential of GRP75 as direct therapeutic target, more studies still are necessary to design inhibitors with high activity and selectivity.

**eHSP70 selective inhibitors**

Extracellular HSP70 is seen in several malignant tumors, such as GRP78 on cell surface in prostat.^577^ Current inhibition strategies against eHSP70 are focused on the development of antibody therapeutics. In 2009, some antibodies were found to directly bind eGRP78 COOH-terminal domain, further inhibiting cellular proliferation and promoting apoptosis of 1-LN and DU145 prostate cancer cell lines.^578^ Besides, HSP70 in the membrane of exosomes can interact with the toll-like receptor 2 (TLR2), involved in tumor development. A8 peptide aptamer was reported to interact with the extracellular domain of membrane HSP70, which blocked HSP70/TLR2 association and further suppressed development of cancer. In addition, A8 enhanced tumor cell sensitivity to chemotherapy drugs, such as cisplatin.^579^ Based on the fact, targeting eHSP70 to discovery selective inhibitors may provide a potential strategy against cancer.

##### Stage 3: targeting PPIs between HSP70 and co-chaperones (from 2010s)

**HSP70-HSP40 PPIs inhibitors**

HSP40, as an important class of co-chaperone, activates the ATPase activity of HSP70, and functions on the HSP70 network.^580^ Therefore, inhibition of HSP70-HSP40 PPIs was also known as an effective strategy for suppressing HSP70 cycle (Fig. 8c). NSC-630668-R/1 was the first reported small molecule that inhibits endogenous HSP40-induced ATPase activity of HSP70 by blocking the HSP70-HSP40 PPIs.^561^ Subsequently, a number of derivatives were reported, including MAL3-101 that specifically prevents a HSP40-dependent stimulation of HSP70 ATPase activity, without affecting endogenous HSP70 activity. The HSP70 ATPase activity was reduced 4-folds by MAL3-101 at 300 μM and the inhibitory effect of MAL3-101 was concentration-dependent. MAL3-101 was showed to compromise the HSP70/HSP40-mediated post-translational translocation of a secreted pre-protein in vitro, which indicated the treatment potential for specific inhibitors of HSP70-HSP40 PPIs in diseases.^74^

**HSP70-BIM PPIs inhibitors**

BIM is another co-chaperone, stimulating the ATPase activity of HSP70 and stabilizing the binding client proteins with HSP70 (Fig. 8c). In most recent years, HSP70-BIM PPIs were identified to stabilize oncogenic proteins and prevent apoptosis.^273,581^ The first-in-class HSP70-BIM PPIs inhibitor, S1g-2, was discovered by screening the a Bcl-2 inhibitor library. S1g-2 specifically inhibited the HSP70-BIM PPIs with IC_50_ of 0.40 μM by directly binding to HSP70, while its detailed binding mechanism was unclear at that time. Importantly, S1g-2 exhibited high apoptosis-inducing activity in chronic myeloid leukemia (CML) cells, making it a completely new class of HSP70 inhibitory strategy.^581^ Subsequently, S1g-10 was yielded by optimizing S1g-2, exhibiting a 10-folds increase in HSP70-BIM PPIs suppressing potency.^582^ Besides, the binding mechanism of the class inhibitors was further explored based on another S1g-2 derivative, S1g-6. The results of MD simulation identified that Tyr149, Thr222, Ala223, and Gly224 of HSP70 are the “hot-spot” in the HSP70-BIM PPIs interface, contributing to the formation of HSP70-S1g-6 complex. Overall, HSP70-BIM PPIs have been confirmed as a potential target for CML therapy.^583^

**HSP70-NEFs PPIs inhibitors**

NEFs family, such as BAGs (BAG1, BAG2 and BAG3), is a vital class of co-chaperones, stimulating disassociation of ADP and substrate from HSP70.^584^ Therefore, HSP70-NEFs PPIs were identified as a potential therapeutic target in cancer. YM-1, an allosteric small inhibitor of HSP70, was reported to effectively disrupt HSP70-BAG3 PPIs with IC_50_ value of 4.8 μM in 2014 (Fig. 8c). YM-1 could interact with NBD of HSP70 in an ADP-bound form and promote its structural changes, which thus weakened the affinity of HSP70 for BAG3. Further studies found that YM-1 inhibited interaction between HSP70 and BAG3.^585,586^ Subsequently, several YM-1 analogs were also reported, including JG-48 and JG-98. JG-48 was found to effectively reduces client release from HSP70 and inhibit endogenous tau in SHSY-5Y neuroblastoma cells. However, apart from BAGs, JG-48 also disrupted the PPIs of HSP70 for other co-chaperons, suggesting that this inhibitory activity is not specific.^587^ JG-98, showed tighter binding of HSP70 than YM-1, with over 60-folds affinity (JG-98/YM-1: Kd = 86/5800 nM). Therefore, JG-98 showed more potent destabilization for the HSP70-BAGs interaction in vitro and in cells (IC_50_ = 1.6 μM). Further studies found that JG-98 exhibited anti-proliferative activity against MCF7 and HeLa cells and possessed ideal pharmacokinetic properties. Importantly, benzothiazole substitution analogs of JG-98 were reported to penetrate blood brain barrier and reduce phosphorylated tau accumulation, which suggested that the HSP70-BAG3 interaction may be a promising target for further exploration.^588,589^ In 2018, a novel HSP70-BAG3 PPIs inhibitor, IT2-144, was discovered by HTS campaign using the human HSP70-BAG2 system. Mechanistic studies revealed that IT2-144 also bound to allosteric sites on HSP70, without affecting inherent ATPase activity of HSP70, providing insights for design new inhibitors targeting HSP70 system.^590^

**PPIs inhibitors of HSP70-co-chaperones containing TPR domain**

Although many co-chaperones containing TPR domain, such as HOP, CHIP and PP5,^286,591,592^ have been reported to interact with HSP70, this class of PPIs inhibitors were rarely discovered (Fig. 8c). In 2019, Shelli R. McAlpine reported the first class of HSP70-HOP PPIs inhibitors. They designed a series of peptides analogs based on the helixes from TPR1 domain that bind to HSP70. Binding assay in vitro found that C1 stabilizes HSP70-HOP interaction, blocking the release of free HSP70. Based on peptide C1, two analogs, SY7, and SY8, were produced and identified to inhibit the HSP70-HOP PPIs with rate of inhibition of approximately 50% at 50 μM. Subsequently, SY7 and SY8 were proved to bind to HSP70 SBD, which disrupted the PPIs of HSP70 and TPR2B domain of HOP and further inhibited the protein folding cycle of HSP70.^593^ SY7 and SY8 provides some insights for designing PPIs inhibitors of HSP70-co-chaperones containing TPR domain. However, due to many proteins containing TPR domain, specificity will be a great challenge in developing the class of inhibitors.

#### Targeting HSP110

**Fig. 9:**
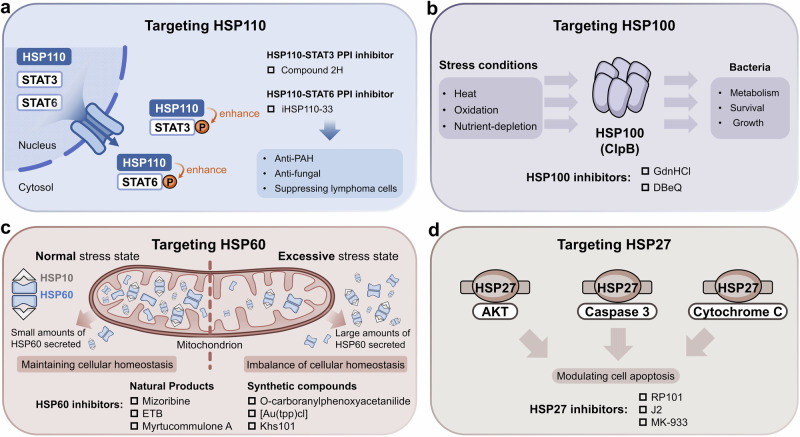
Targeting therapy of other HSP members. **a** Inhibitors targeting HSP110. **b** Inhibitors targeting HSP100. **c** Inhibitors targeting HSP60. **d** Inhibitors targeting HSP27

Compound 2H was found to directly bind to HSP110, preventing its interaction with STAT3 (Fig. 9a). This inhibition suppresses the downstream expression of p-STAT3 and c-Myc, ultimately inhibiting the growth of human pulmonary arterial endothelial cells (HPAECs). Further studies indicated that 2H has good potential to treat pulmonary arterial hypertension (PAH).^594^ Meanwhile, it was the first inhibitor targeting candida albicans HSP110 (Msi3), significantly inhibits its chaperone activity with IC_50_ of 5.0 μM (Table 5). This inhibition disrupts overall protein homeostasis, thereby inhibiting fungal growth and biofilm formation. Additionally, due to its ability to retain NEF activity, 2H serves as an important chemical tool for studying HSP110 function and is a promising candidate for antifungal drug development.^595^ Another synthetic compound, iHSP110-33, can directly bind to the NTD of HSP110 with an IC_50_ of 58 μM (Table 5). Though interfering with the interaction between HSP110 and its client protein STAT3, iHSP110-33 prevents the phosphorylation of STAT3. Consequently, it effectively reduces tumor growth in mouse models by inducing apoptosis in tumor cells.^596^ Recently, researchers discovered that iHSP110-33 can inhibit the phosphorylation of STAT6 by interfering with the HSP110-STAT6 interaction. This inhibition subsequently suppresses the growth and survival of lymphoma cells.^597^ Further studies also confirmed that iHSP110-33 can reduce the phosphorylation levels of B cell receptor (BCR) signaling kinases, inhibiting the growth and survival of cancer cells.^598^

**Table 5:** Preclinical studies of targeting other molecular chaperones

Compound	Structure	Activity	Mechanism (Sites)
Compound 2H		^c^IC_50_ = 5.0 µM	HSP110 Inhibitor (/)
iHSP110-33		^b^IC_50_ = 58 μM	HSP110 Inhibitor (NBD)
GdnHCl		K_d_ ≈ 600 μM	ClpB Inhibitor (NTD)
DBeQ		^a^IC_50_ ≈ 5.0 μM	ClpB Inhibitor (NTD)
Mizoribine		/	HSP60 inhibitor (NBD)
ETB		^c^IC_50_ = 1.1 μM	HSP60 inhibitor (Cys442 covalent site)
MC		/	HSP60 inhibitor (/)
O-carboranyl-phenoxyacetanilide		^c^IC_50_ = 0.74 μM	HSP60 inhibitor (NBD)
[Au(TPP)Cl]		^c^IC_50_ = 1.1 μM	HSP60 inhibitor (/)
KHS101		^c^IC_50_ = 14 μM	HSP60 inhibitor (/)
RP101		/	HSP27 inhibitor (Phe29 and Phe33)
J2		^c^IC_50_ = 99 μM	HSP27 inhibitor (Cysteine-thiol group)
MK-933		/	HSP27 inhibitor (NTD)

*K*_*d*_ target binding affinity, ^*a*^*IC*_*50*_ ATPase inhibitor activity, ^*b*^*IC*_*50*_ competitive binding activity, ^*c*^*IC*_*50*_ inhibitory activity on molecular chaperone functions, “/” indicates no clear reports

#### Targeting HSP100

While exploring small molecule inhibitors targeting HSP100, researchers primarily focused on inhibitors of ClpB, a homolog of HSP100 from bacteria. ClpB mainly mediates tolerance to stressful conditions, including heat, oxidation, nutrient-depletion, contributing to metabolism, survival and growth of bacterial during infection (Fig. 9b).^599^ Guandine chloride (GdnHCl) is an organic salt, which efficiently inhibits the ATPase activity of ClpB in vitro by binding the NTD of ClpB.^600,601^ Despite its low binding affinity with a K_d_ value of approximately 600 μM, GdnHCl can serve as a useful tool for exploring the biological function of ClpB in pathogens (Table 5). A quinazoline derivative, N^2^, N^4^-dibenzylquinazoline-2,4-diamine (DBeQ), has been found to target ClpB of *E. coli* under heat stress conditions. It binds to ClpB in a competitive manner with ATPase, exhibiting an IC_50_ value of approximately 5.0 μM (Table 5).^602^

#### Targeting HSP60

HSP60 is mainly observed in the mitochondria, where HSP60-HSP10 complex facilitates mitochondrial protein folding. In the excessive stress state, large amounts of HSP60 are secreted, resulting in imbalance of mitochondrial and cellular homeostasis (Fig. 9c).^86^ Small molecule regulators targeting HSP60 mainly are natural productions, including mizoribine, epolactaene’s tert-butyl ester^603^ and myrtucommulone A (MC),^604^ and the synthetic compounds O-carboranylphenoxyacetanilide,^605^ Gold (III) porphyrin complexes [Au (TPP) Cl],^606^ KHS101^607^ and KIRA6 (Table 5).^608^ Mizoribine is a natural imidazole riboside antibiotic, binding to the ATP pocket of HSP60 at high concentrations. Though highly selective inhibiting the formation of HSP60-HSP10 complex, the antibiotic disrupts folding functions of HSP60 for client proteins. Studies also found mizoribine with strong immunosuppressive activity, which proved the potential of HSP60 as a drug target.^609–611^ Epolactaene and its tert-butyl ester ETB have close activity in inhibiting HSP60.^603^ Mass spectrometry and competitive binding experiments demonstrated that ETB selectively binds HSP60 rather than other chaperone proteins such as HSP70 and HSP90.^612^ ETB covalently bound to Cys442 site of HSP60 and promoted its alkylation to allosterically inhibit its folding activity at the IC_50_ level of 1.1 μM, without inhibiting ATPase activity of HSP60.^603,613^ In addition, the allosteric regulation of ETB further also inhibited the formation of HSP60-HSP10 complex.^86^ Myrtucommulone A (MC) is a non-prenylated acylphloroglucinol. MC inhibits the activity of HSP60-HSP10 complex by targeting HSP60, thereby affecting their mitochondrial function,^604^ including inhibiting ATP synthesis and canceling the aggregation protection of mitochondrial proteins.^614^ Therefore, MC can induce apoptosis of tumor cells, without toxicity to normal cells.^615^ Among synthetic inhibitors, researchers found that the boron-containing molecule o-carboranylphenoxyacetanilide exhibits HIF-1α inhibitory activity, with an IC_50_ value of 0.74 μM.^605^ Experimental validation using designed probes confirmed that this mechanism is achieved by specifically targeting HSP60 ATP-binding pocket, inhibiting the activity of HSP60-HSP10 complex.^613^ [Au (TPP) Cl] is a gold (III) porphyrin complex, which is more stable under physiological conditions due to its strong donor ligand.^616^ Using a chemoproteomic strategy, researchers have identified that the compound directly targets HSP60. It interacts with HSP60 both in vivo and in vitro, and inhibits the function of the HSP60-HSP10 complex. The electrophilic action of gold (III) ions and the hydrophobic interactions of the porphyrin ligand are critical for binding with HSP60.^606^ KHS101 directly inhibits mitochondrial HSP60 with an IC_50_ of 14 μM. This small molecule HSP60 inhibitor interferes with cancer cell energy metabolism by affecting protein folding activity without impacting HSP60 expression levels or normal cell proliferation.^607,617^

#### Targeting sHSPs

Current research on inhibitors targeting sHSPs primarily focuses on HSP27 (Table 5). RP101, a thymidine analog, binds to the Phe29 and Phe33 sites of HSP27, inhibiting its interaction with proteins such as AKT1, caspase 3, and cytochrome C.^618^ This inhibition influences apoptosis and enhances the efficacy of other anticancer drugs (Fig. 9d).^619^ J2 is a synthetic dye compound that covalently binds to the cysteine-thiol group of HSP27. Researchers have found that J2 inhibits HSP27 with an IC_50_ of 99 μM, thereby suppressing HSP27-mediated cancer resistance.^620^ MK-933 interacts with the NTD of the HSP27 dimer. This binding prevents HSP27 phosphorylation and subsequent depolymerization, thereby inhibiting its chaperone activity and regulation of client proteins^66^ (Fig. 9d).

## Conclusions and perspectives

Molecular chaperones play significant roles in cellular stress response, protein quality control, and cell signaling transduction processes, which has always been a captivating topic in the scientific research field. There have been several notable reviews describing their biological mechanisms and development of inhibitors.^4,15,621–623^ In this review, we attempt to take a broader perspective on molecular chaperones by giving details of their biological structure, regulatory mechanism and targeted strategies in various development stages.

Currently, there has been a deeper understanding of the structures and functions of HSPs, as well as their collaboration as molecular chaperones in regulating proteostasis and stress management. In addition to the biological structures of various HSP molecular chaperone members were gradually elucidated, the structures of protein complexes within the HSP molecular chaperone systems were also progressively revealed. We describe the biological characteristics of these structures and summarized their development, including the binary complexes, ternary complexes, tetrameric complexes, which provides crucial evidence for understanding the regulatory mechanisms of HSP molecular chaperone systems. However, there are still many unclear details about the structures and mechanisms of HSPs. HSPs work as complex molecular chaperone network in maintaining protein homeostasis, in which many transient and dynamic complex structures would be formed between HSPs and co-chaperones or client proteins. The instability of these transient and dynamic complex structures is difficult to resolve, resulting in that the details of structure and mechanism of HSPs in protein folding cycle are not completely understood.

**Fig. 10:**
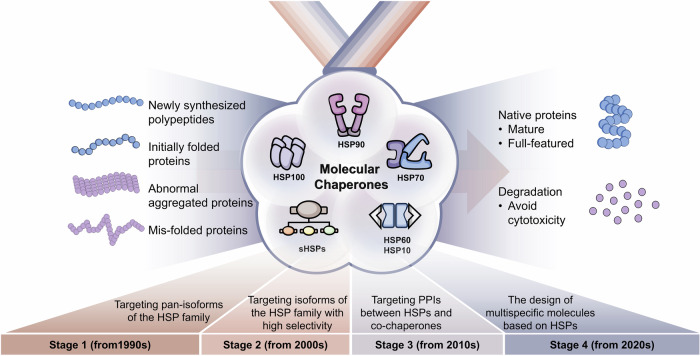
Summary of biological function and targeting strategies of molecular chaperones. HSPs, as main molecular chaperone, assist in the folding of newly synthesized polypeptides and initially folded proteins into their native form, while also promoting the degradation of mis-folded proteins and abnormal aggregated proteins. Among HSPs, HSP100, HSP90, HSP70, HSP60-HSP10, and sHSPs act as molecular chaperones to regulate protein homeostasis, and HSP40 and large HSPs, as co-chaperones, assist them in playing the role of molecular chaperones. As disease targets, HSP90 and HSP70 have been the focus of extensive research, while other members of HSP families have received less attention. Overall, their targeting strategies can be classified into four development stages: stage 1, targeting pan-isoforms of the HSP families (from1990s); stage 2, targeting isoforms of the HSP families with high selectivity (from 2000s); stage 3, targeting PPIs between HSPs and co-chaperones (from 2010s); and stage 4, the design of multi-specific molecules based on HSPs (from 2020s)

In fact, various HSP members exhibit distinct biological mechanisms. Small HSPs play their roles in an ATP-independent way. Small HSPs can form oligomers through interactions with themselves or other small HSP members, working as holdase to prevent aggregation and sequestrate misfolded proteins. Most HSP members are ATP-dependent, including HSP100, HSP90, HSP70, and HSP60, which usually are involved in more complex biological mechanisms. HSP100 chaperone typically forms homohexamer rings containing substrate interaction sites to refold misfolded-proteins or disassemble irreversible protein aggregates. HSP90 functions as a flexible dimer, involving an opening-closing dynamic cycle that allows HSP90 to interact with client proteins and promote their correct folding and release. At the same time, HSP90 need many co-chaperones to regulate its activities and assist in the completion of the molecular chaperone cycle. Unlike HSP90, HSP70 performs functions through a two-domain monomer, with the help of many co-chaperones. HSP60 can form an isolation chamber for the substrate protein through interacting with HSP10 to assist folding of proteins. These ATP-dependent HSPs have distinct mode of ATP binding, which helps HSPs play their respective roles in different cellular states. Although the mechanism of HSPs has been studied extensively, there are still many unanswered questions. HSPs regulate the folding and release of multiple proteins, including kinases, transcription factors, E3 ubiquitin ligases, nuclear hormone receptors, cytoskeletal proteins, signal-transduction proteins, epigenetic regulatory proteins, cyclin proteins, and telomerase. However, the specific mechanism how HSPs recognize various types of substrate proteins is still ambiguous. Apart from regulating proteins folding and release, HSPs also have a key function in regulation of various PTMs of clients (Fig. 10). Recently, Pearl and Agard identified that PP5, as a co-chaperone, assists in phosphorylation of proteins in HSP90 molecular chaperone network,^46,50^ which provides some insight for the biological mechanisms related to phosphorylation of substrate proteins. However, the types of PTMs of proteins are diverse, such as ubiquitination, acetylation, butyrylation and glycosylation. Hence, there is considerable research opportunity to investigate how SHPs employ diverse co-chaperones for various PTMs.

HSPs have been reported to closely related to the occurrence and development of various diseases. We generalized the connections between HSPs and various types of diseases, which includes cancers, neurodegenerative diseases, cardiovascular diseases, inflammatory diseases, metabolic diseases, infectious diseases, ocular diseases, skin diseases. HSPs are highly upregulated in most diseases, providing advantages for diseases progress by releasing large amounts of pathogenic proteins. Although the functions of HSPs in disease have been widely studied, the comprehensive understanding of HSPs regulation mechanism in diseases remains limited. First, HSP members have different isoforms located in various intracellular and extracellular structures, which may play distinct functions in the different disease. Besides, HSPs interact with other co-chaperones and proteins to form a complex molecular chaperone networks that plays different roles in various cellular environments and disease. Therefore, the specific regulatory mechanisms and pathogenesis of HSPs in different types of diseases still need to invest a lot of research. Nevertheless, HSPs, especially HSP90, have been found to not only help maintain the activity and stability of a variety of oncogenic proteins (e.g., kinases, transcription factors, etc.) in tumor cells, but also participate in the regulation of tumor cell resistance to chemotherapeutic agents. HSPs also affect tumor microenvironment, where it may be involved in regulating the interactions between the tumor cells and their surrounding environment, which are critical for tumor growth and metastasis.^4^ Therefore, HSPs may become attractive targets for cancer therapy. HSPs is differentially expressed in different tumor tissues, suggesting that therapeutic strategies to inhibit HSPs may be more effective for certain types of cancers, especially those in which HSPs are overexpressed. For example, HSP90 is highly expressed in a variety of tumor tissues, including gastric, lung, liver, and breast cancers.^624^ The expression of HSP70 in cervical cancer was significantly higher than that in normal tissues.^625^ Therefore, inhibitors targeting HSPs may be more effective on inhibiting various cancers.

Finally, we analyze the clinical research progress targeting HSPs, of which most indications are various solid tumors. However, many HSPs inhibitors in clinical trials were suspended or terminated because of either toxicity or insufficient efficacy. Besides, most completed clinical trials also remain stalled at the clinical stage without significant advancement. Therefore, developing drugs targeting HSPs still faces great challenges and limitation. Currently, reported inhibitors targeting HSPs in preclinical trials mainly can be divided into three classes, including natural product, peptide and synthetic small molecule derivative. In order to avoid the disadvantages of natural products and peptide drugs, synthetic derivatives were extensively obtained by different drug discovery methods, of which some inhibitors showed more potent activity and higher selectivity targeting HSPs. Based on the fact, the process of inhibitors targeting HSPs in preclinical can be classified into four development stages, including stage 1: targeting pan-isoforms of the HSP families (from 1990s), stage 2: targeting isoforms of the HSP families with high selectivity (from 2000s), stage 3: targeting PPIs between HSPs and co-chaperones (from 2010s), and stage 4: designing multi-specific molecules based on HSPs (from 2020 s). In the stage 1, in addition to inhibitors entered into clinical trials, many inhibitors were also found to bind with ATP pocket of HSPs without selectivity. Although they demonstrated improved biological activity, their poor druggability greatly limited their further clinical applications. The selective inhibitors of the HSPs isoforms in stage 2 were subsequently designed to interact with specific binding sites. Although overcoming the side effect result from poor isoform selectivity, they degraded the substrate proteins indiscriminately due to complete inhibition of the chaperone activity of the HSPs. Gradually, researchers began investigating how to achieve selective modulation of substrate proteins, which facilitated the development of inhibitors of HSPs into stage 3. Since co-chaperones mediate substrate protein with selectivity, targeting PPIs between HSPs and co-chaperones in stage 3 achieved fine regulation for substrate protein, providing novel mechanism targeting HSPs. As the design of multi-specific molecules is gradually expanding, the discovery of CHAMPs and PROTACs based on HSP90 provides a novel strategy for targeting HSPs. As a novel CHAMP, RNK05047 was design to selectively degrade BRD4 in tumors. RNK05047 entered clinical studies in 2024, which brings new hopes and opportunities for future HSPs inhibitory strategies (Fig. 10).

Although effective drugs targeting HSPs have not yet been approved by the FDA, HSPs has been conclusively linked to tumor survival, proliferation, and drug resistance in cancer therapy. HSPs inhibitory strategies have unique biological mechanisms. On the one hand, HSPs inhibitors are able to specifically interfere with their molecular chaperone functions, leading to the disruption of protein homeostasis in cancer cells. On the other hand, targeting HSPs may induce cancer cell death by affecting the survival environment of tumor cells. Compared with tyrosine kinase inhibitors (TKIs),^626^ HSPs inhibition may be able to address their acquired resistance. In addition, compared with immunotherapy,^627^ inhibitors of HSPs might be able to avoid immune-related adverse effects and suppress tumors that lack T-cell infiltration. Although still in the relatively early stages, targeting HSPs are expected to provide new treatment options for cancer patients as research progresses and clinical trials are conducted in the future. Besides, HSPs inhibitors have shown therapeutic potential in other diseases, which holds opportunities for the development of more potent HSPs inhibitors.
