# Correction: Advances in the structures, mechanisms and targeting of molecular chaperones

**DOI:** 10.1038/s41392-025-02215-w

**Published:** 2025-04-02

**Authors:** Jinying Gu, Yanyi He, Chenxi He, Qiuyue Zhang, Qifei Huang, Shangjun Bai, Ruoning Wang, Qidong You, Lei Wang

**Affiliations:** 1 State Key Laboratory of Natural Medicines and Jiangsu Key Laboratory of Drug Design and Optimization, China Pharmaceutical University, Nanjing, China; 2 Department of Medicinal Chemistry, School of Pharmacy, China Pharmaceutical University, Nanjing, China; 3 School of Pharmacy, Nanjing University of Chinese Medicine, Nanjing, China; 4 Jiangsu Provincial TCM Engineering Technology Research Center of Highly Efficient Drug Delivery Systems (DDSs), Nanjing, China

Correction to: *Signal Transduction and Targeted Therapy* 10.1038/s41392-025-02166-2, published online 12 March 2025

Following the online publication of article,^1^ an error was identified in Figures 6, 7, and 8, which were inadvertently swapped during the proof correction. This has been rectified in the corrected version of the article.

Additionally, author contributions statement was not submitted and published in the original version. The missing contribution statement is: “Contributed as co-first authors: Jinying Gu, Yanyi He, Chenxi He, Qiuyue Zhang.”

The original incorrect figures and corrected figures are provided below.

Incorrect Figure 6
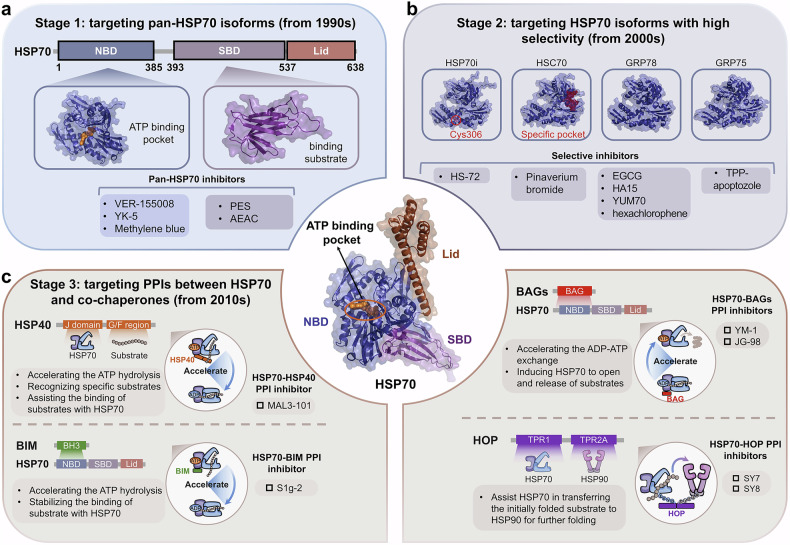


Correct Figure 6
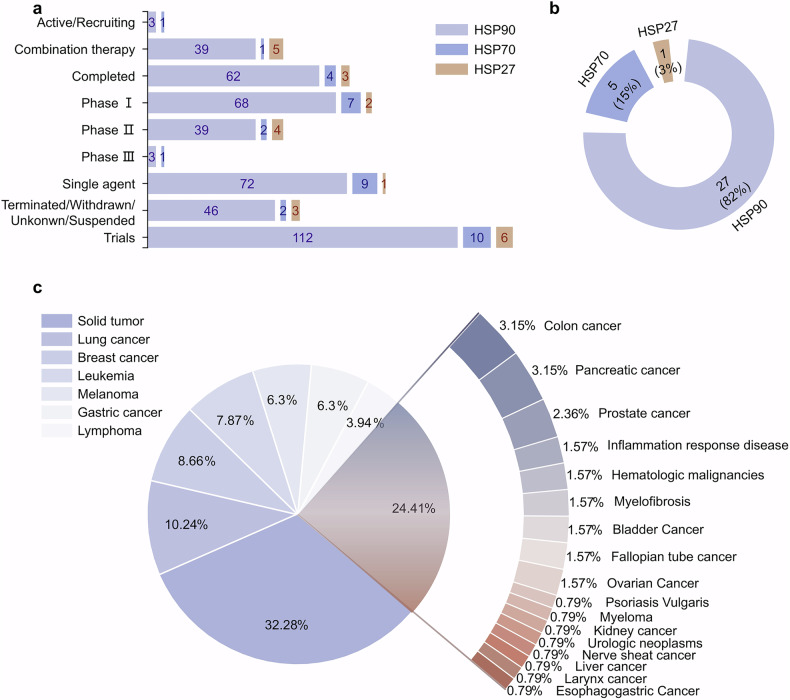


Incorrect Figure 7
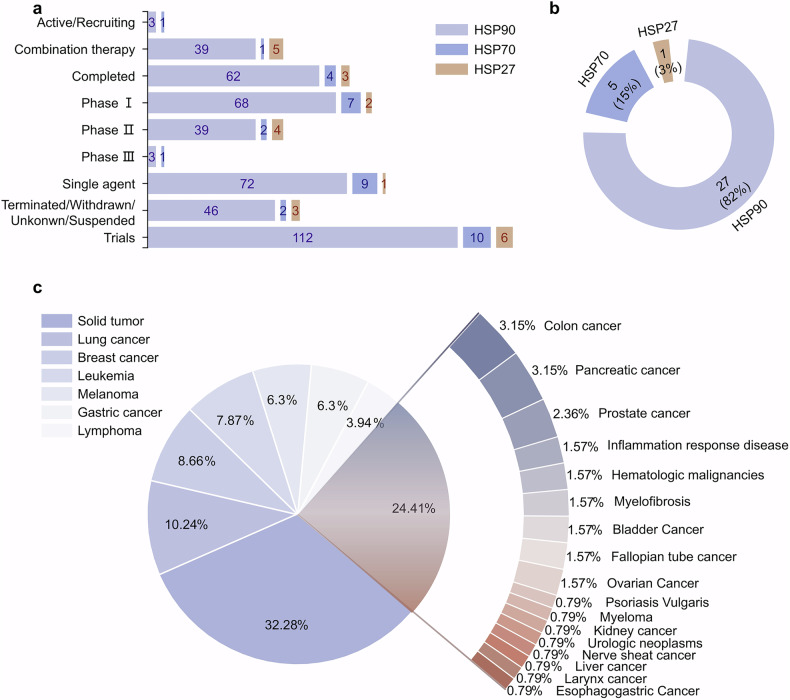


Correct Figure 7
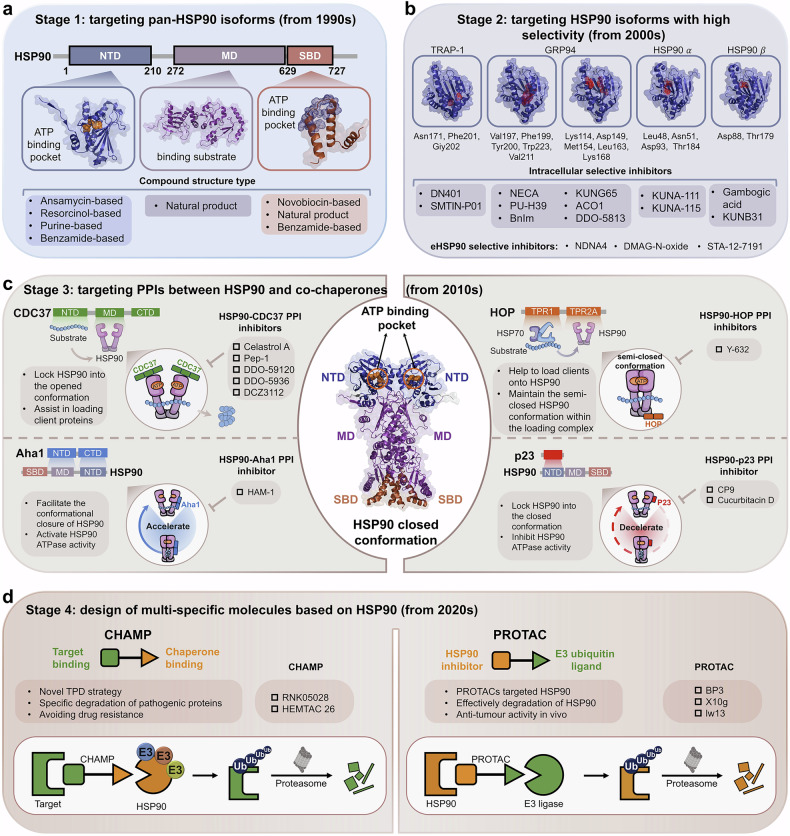


Incorrect Figure 8
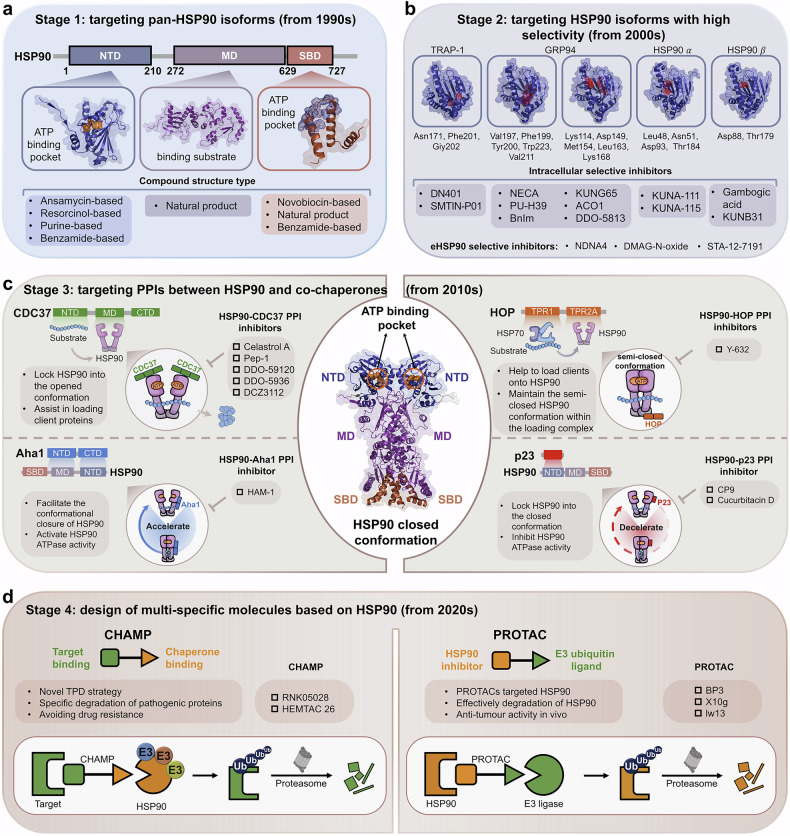


Correct Figure 8
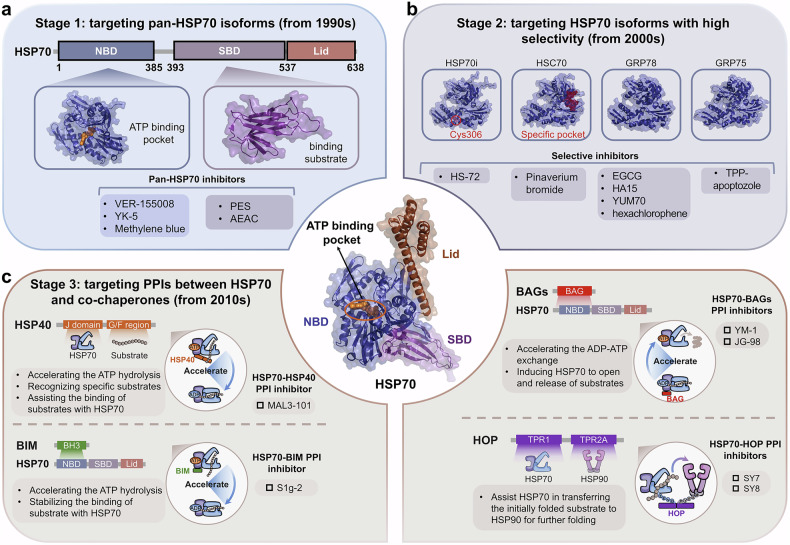


The original article has been corrected.
